# Characterization of the pigmented shell-forming proteome of the common grove snail *Cepaea nemoralis*

**DOI:** 10.1186/1471-2164-15-249

**Published:** 2014-03-31

**Authors:** Karlheinz Mann, Daniel John Jackson

**Affiliations:** 1 Max Planck Institute for Biochemistry, Department of Proteomics and Signal Transduction, Am Klopferspitz 18, D-82152 Martinsried, Munich, Germany; 2 Courant Research Centre Geobiology, Georg-August University of Göttingen, Goldschmidtstrasse 3, 37077 Göttingen, Germany

**Keywords:** Biomineralization, Calcification, Mollusc, Pulmonate, Pigment, Shell, Protein, Evolution, *Cepaea nemoralis*

## Abstract

**Background:**

With a diversity of pigmented shell morphotypes governed by Mendelian patterns of inheritance, the common grove snail, *Cepaea nemoralis,* has served as a model for evolutionary biologists and population geneticists for decades. Surprisingly, the molecular mechanisms by which *C. nemoralis* generates this pigmented shelled diversity, and the degree of evolutionary conservation present between molluscan shell-forming proteomes, remain unknown.

**Results:**

Here, using next generation sequencing and high throughput proteomics, we identify and characterize the major proteinaceous components of the *C. nemoralis* shell, the first shell-proteome for a pulmonate mollusc. The recent availability of several marine molluscan shell-proteomes, and the dataset we report here, allow us to identify 59 evolutionarily conserved and novel shell-forming proteins. While the *C. nemoralis* dataset is dominated by proteins that share little to no similarity with proteins in public databases, almost half of it shares similarity with proteins present in other molluscan shells. In addition, we could not find any indication that a protein (or class of proteins) is directly associated with shell pigmentation in *C. nemoralis*. This is in contrast to the only other partially characterized molluscan-shell pigmentation mechanism employed by the tropical abalone *Haliotis asinina*.

**Conclusions:**

The unique pulmonate shell-forming proteome that we report here reveals an abundance of both mollusc-specific and pulmonate-specific proteins, suggesting that novel coding sequences, and/or the extensive divergence of these sequences from ancestral sequences, supported the innovation of new shell types within the Conchifera. In addition, we report here the first evidence that molluscs use independently evolved mechanisms to pigment their shells. This proteome provides a solid foundation from which further studies aimed at the functional characterization of these shell-forming proteins can be conducted.

## Background

The evolutionary origins, mode of construction, patterning, and physical properties of the molluscan shell have held the attention of scientists for centuries. However, the molecular mechanisms by which these structures are constructed are only beginning to be elucidated [1-3]. The molluscan shell is assembled extracellularly and is an ensemble of CaCO_3_ and organic macromolecules (proteins, pigments, glycoproteins, lipids and polysaccharides) which are secreted by an organ known as the mantle. The anterior edge of the mantle underlies the lip of the shell and directs the ordered biomineralization of the different structural layers of the shell and also controls the deposition of pigment features. With advances in nucleic acid sequencing technologies and proteomic methods, the close to complete shell-forming proteomes of several molluscs have now been reported [4-7]. Several proteins from these collections have been more fully characterized [8-11]. However, the vast majority of these previous studies are focused on marine species. While Pavat et al. [12] recently reported the biochemical properties of the shell forming proteome of the pulmonate *Helix aspersa maxima*, the lack of any transcriptome or genome data for this species limited their proteomic analyses. They were able to characterize nine distinct 2D spots, of which seven returned a total of 14 peptides ranging in length from 4 – 11 residues. A full proteome-scale dataset from a pulmonate gastropod would efficiently highlight the conserved and lineage specific molecular mechanisms of molluscan shell formation, and would provide deep insight into how these proteomes evolve. This is because marine and terrestrial shell-forming molluscs have adapted to significantly different environments that would fundamentally affect both the process of shell formation, and the stability of the secreted composite biomineral; e.g. the abundance and biological availability of calcium, environmental pH, temperature, UV radiation, humidity and so on. While proteins involved in the process of shell formation in different molluscan lineages could be expected to have evolved in response to these different selective pressures, the signature of an ancestral shell-forming program may still be recognizable.

**Figure 1:**
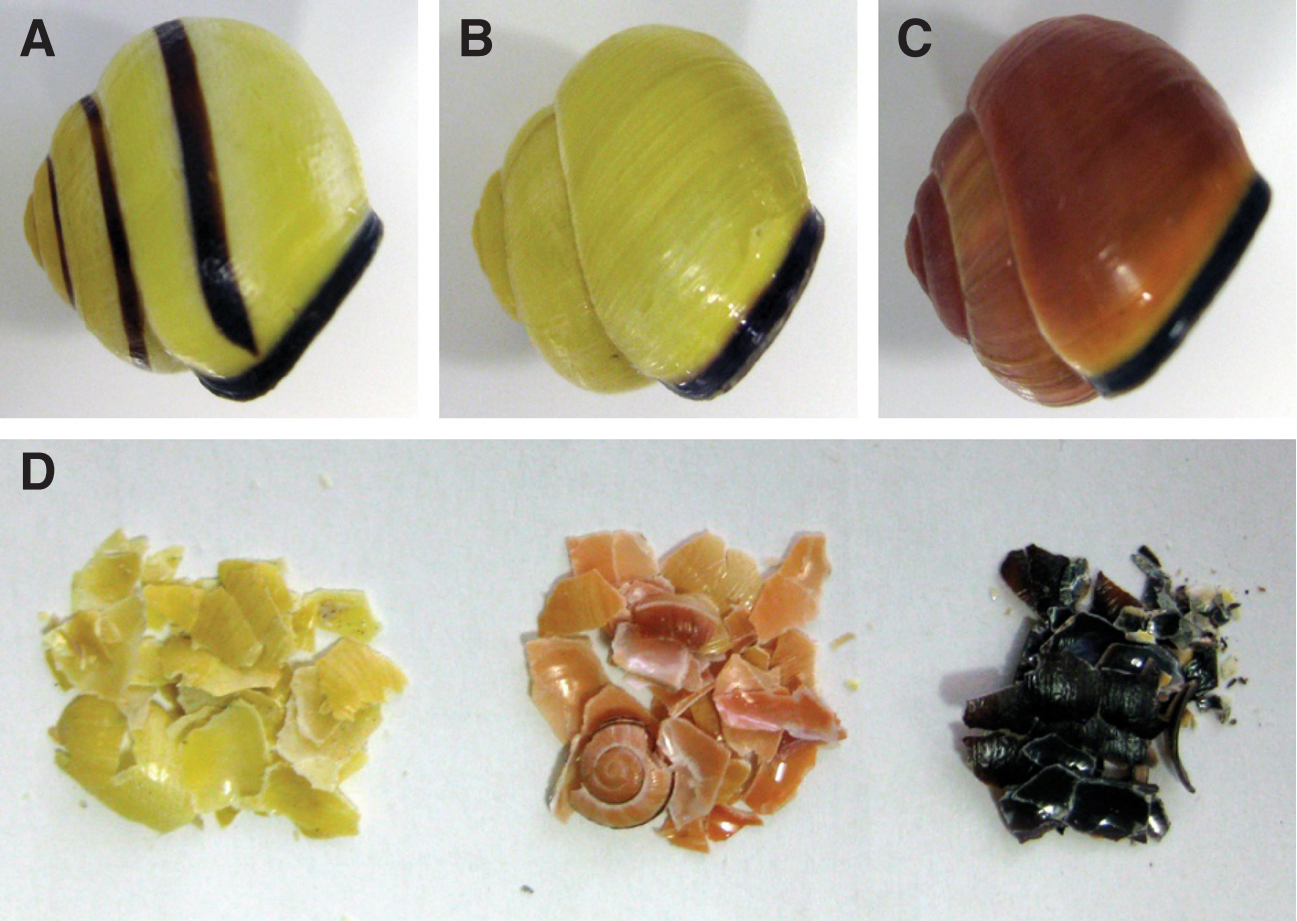
**Representative polymorphic shells of *C. nemoralis *surveyed for their protein contents. A–C**. Examples of the three main shell types we surveyed for both shell-forming proteins and protein-associated pigments **D**. In order to identify pigment-associated proteins *C. nemoralis* shells were crushed and divided into one of three pigmented fractions for subsequent proteomic analyses. With this approach, differentially localized proteins would be visible on LDS-PAGE gels.

The common grove snail, *Cepaea nemoralis* (Figure 1), has long been studied by ecologists and population geneticists [13-15]. Key insights were gained during the 1950’s and 1960’s when it was demonstrated that variation in pigmented shell traits of *Cepaea* are inherited in a Mendelian fashion [16,17]. Furthermore, the frequencies of these morphotypes have been suggested to be influenced by two agents of natural selection: predation by birds [18,19], and climatic conditions [20]. Despite this long history of research concerning the variable pigmentation of the *Cepaea* shell, there is only one study that has aimed to specifically identify the genes that control this morphological variety [21]. Using RAD-Seq (Restriction Site Associated-Sequencing) Richards et al. recently identified 44 anonymous markers putatively linked to loci that control the shell ground color and the presence or absence of dark brown bands on the *Cepaea* shell. Yet despite the association of carotenoids, porphyrins, carbohydrates and polyenes with some molluscan shell pigments [22-25], there exists no example of a complete molecular understanding of any shell pigmentation mechanism in any mollusc. In one case, the molecular basis of a molluscan shell pigment been partially elucidated. One of us previously demonstrated that the protein Sometsuke is directly associated with the blue and red pigmentation of the juvenile shell of the tropical abalone *Haliotis asinina*[4]. This previous finding motivated us to search for proteins associated with the various pigments within the *C. nemoralis* shell using high throughput transcriptomic and proteomic methods. This effort has allowed us to assemble a dataset that is likely to represent the majority of the shell forming proteome of *C. nemoralis.* This in-depth proteome is the first to be reported from a pulmonate gastropod, and also allows us to conduct comparisons between it and several others recently reported shell-forming proteomes from marine species.

## Results and discussion

### *General character of the* C. nemoralis *shell-forming proteome*

**Figure 2:**
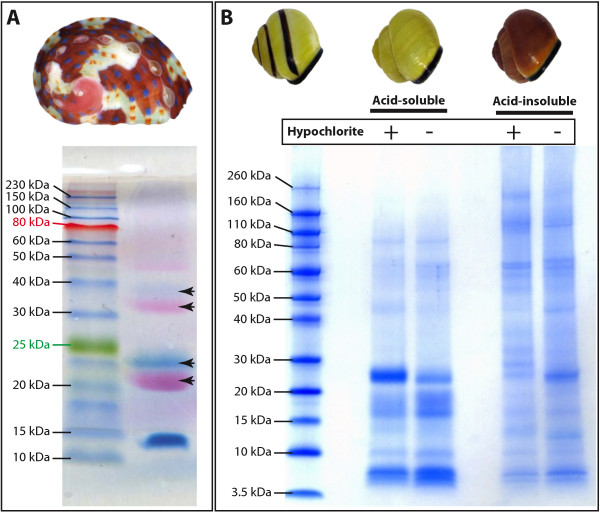
**A comparison of the protein-associated pigments and PAGE profiles of proteins isolated from the shells of *H. asinina *and *C. nemoralis*. A**. A representative SDS-PAGE gel of proteins isolated from shells of juvenile *H. asinina*. This gel is unstained (the protein marker is pre-stained) allowing the red and blue pigment-associated proteins to be visualized (arrows). **B**. A representative LDS-PAGE analysis of *C. nemoralis* acid-soluble and –insoluble proteins extracted from shell fragments either treated with or without a sodium hypochlorite-plus-sonication pre-treatment. Protein extractions which displayed such electrophoretic patterns were subjected to FASP (Filter-Aided Sample Preparation) sample preparation and proteomic analysis.

We were able to retrieve a total of 553 proteins/protein groups from the shell proteome of *C. nemoralis* using two different preparative techniques (including or excluding a sodium hypochlorite-plus-sonication pretreatment step). Shell material that had not been washed yielded 418 proteins, while shell material washed with sodium hypochlorite and sonication yielded 525 proteins. 382 proteins were present in both of these datasets. A list of accepted identifications is provided in Additional file 1. The MaxQuant output files containing relevant parameters, such as scores, sequence coverage and number of peptides are provided for proteins/protein groups in Additional file 2 (matrix extracted without hypochlorite pre-treatment) and Additional file 3 (with hypochlorite pre-treatment). Additional file 4 (without hypochlorite treatment) and Additional file 5 (with hypochlorite treatment) contain the MaxQuant output files for peptides. All MaxQuant output files also contain proteins rejected after manual validation of the results, and are therefore not included in the list of accepted identifications in Additional file 1. An iBAQ estimate of protein abundance suggests that 59 proteins/protein groups constitute more than 93% of the total *Cepaea* shell proteome (Table 1). This is a conservative collection of proteins and peptides that met or exceeded stringent bioinformatic and statistical criteria (see Methods). With further work (for example more complete *Cepaea* transcriptomic and/or genomic datasets against which to query the MS data) this estimate will likely increase. It has been reported for corals that in order to reduce the presence of contaminating non-biomineralizing proteins, an extensive sodium hypochlorite cleaning step must be performed on the finely powdered coral biomineral [26]. If this is not done then abundant cytoskeletal proteins such as actins, tubulins and myosins (which are unlikely to be directly involved in biomineralization) will carry through into the final biomineralization dataset [27]. While we observed subtle differences in the LDS-PAGE profiles of proteins derived from *C. nemoralis* shell material prepared with and without a hypochlorite pre-treatment (Figure 2), the proteomic data generated by the two methods were not fundamentally different.

**Table 1:** **The major proteins and peptides of the *C. nemoralis *shell: 59 proteins and peptides (with an iBAQ percentage of more than 0.1) constitute 93**% **of the identifiable *C. nemoralis *shell proteome**

**Isotig**	**Similarity to (best match)**	**E-value**	**Identity**	**Protein features**^**1**^	**Fraction**	**Unique + razor peptides**	**% of total (iBAQ)**
**123**	None	-	-	(12% G, 18% P)	S > I	15	26.22
**1152**	None	-	-	(16% G, 15% M, 10% S); SP	S > I	3	15.55
**7508**	None	-	-	SP	S < I	15	6.44
**1265**	None	-	-	(12% G)	S > I	9	4.40
**2668**	Q4LDE5	2.0e-8	32%	Domains: Sushi/SCR/CCP; (10% A, 13% G, 10% S); SP	S > I	13	3.87
	*Homo sapiens*						
**21112**	None	-	-	(18% P, 10% N)	S > I	8	3.41
**14344**	None	-	-	(17% Q, 10% L, 17% P)	S < I	11	2.96
**821**	J7Q5J6	8.7e-11	29.9%	Similar to BSMP; Domains: CBM_14/ CHIT_BIND_II	S < I	15	2.45
	*Patella vulgata*						
**4164**	None	-	-	Similarity to UP2_HALAI (e-value 0.19; 28.1% identity)	S > I	3	2.07
**58150**	K1QZ49	1.2e-40	35.1%	Similar to adipocyte plasma membrane-associated protein; Domains: TolB_like/ strictosidine synthase; see also contig_221 and contig_16710; SP	S < I	16	1.98
	*Crassostrea gigas*						
**5087**	None	-	-	TM	S < I	39	1.60
**35852**	None	-	-	(11% A, 11% P)	S > I	14	1.46
**12941**	None	-	-	(18% D, 14% L)	S < I	12	1.37
**10584**	None	-	-	(16% D, 10% E)	S < I	14	1.35
**2108**	None	-	-	(10% A, 10% Q)	S < I	8	1.32
**1850**	None	-	-	(22% P); SP	S > I	15	1.31
**201**	None	-	-	(11% N, 10% Q); SP	S < I	11	1.20
**1504**	None	-	-	(16% G, 12% L, 21% M, 10% S in 58AA); SP	S > I	1	1.15
**16878**	None	-	-	(29% Q, 16% P)	S > I	3	1.08
**2744**	None	-	-	(10% K, 10% S)	S < I	14	0.90
**551**	None	-	-	(Fragment of 67AA containing 18% G, 22% M, 12% P)	S > I	3	0.85
**7809**	None	-	-	(21% D, 16% L)	S < I	17	0.83
**25891**	J7QJT8	6.3e-25	34.7%	Domains: αCA; SP	S < I	42	0.59
	*Patella vulgata*						
**3938**	None	-	-	SP	S > I	2	0.58
**1188 + 4282**	K1P9P0	1.2e-27	47.0%	Mesenchyme-specific cell surface glycoprotein; Domains:WD40/YVTN repeat-like	S < I	15	0.53
	*Crassostrea gigas*	4.4e-21	50.9%				
**7563**	None	-	-	-	S,I	6	0.51
**6176**	None	-	-	(11% A, 11% G, 12% M)	S > I	7	0.49
**1604**	None	-	-	(31% Q, 23% P)	S > I	2	0.48
**269**	None	-	-	(18% G)	S < I	8	0.44
**14003**	K1PRD3	3.6e-22	30.1%	Similar to IgGFc-binding protein; Domains: CBM_14/CHIT_BIND_II; shares peptide with 101824	S,I	18	0.38
	*Crassostrea gigas*						
**1647**	None	-	-	Domains:Sushi/SCR/CCP; (12% P, 11% S, 11% T)	S > I	9	0.38
**450**	68CYM6	9.9e-67	74.2%	G-type lysozyme;SP	S < I	5	0.36
	*Physella acuta*						
**1323**	A7T0W4	1.3e-26	40.5%	Domains: polysaccharide deacetylase/chitinase	S < I	7	0.32
	*Nematostella vectensis*						
**101824**	K1QJK2	2.1e-22	29.4%	Domains: CBM_14/ CHIT_BIND_II	S < I	29	0.31
	*Crassostrea gigas*						
**84589**	K1QIK2	5.4e-41	29.2%	Domains: CBM_14/ CHIT_BIND_II; also see contig_101824	S < I	29	0.30
	*Crassostrea gigas*						
**132**	None	-	-	(12% Q)	S < I	21	0.29
28994*	K1QPM9	1.5e-22	45.6%	Similar to fatty acid-binding protein, brain	S,I	7	0.24
	*Crassostrea gigas*						
**32297**	None	-	-	(14% G, 10% P); SP	S > I	2	0.23
**74063**	None	-	-	(10% G, 13% L, 20% P); SP	S,I	1	0.22
**263**	None	-	-	-	S < I	1	0.21
**63304**	K1Q365	9.6e-55	29.8%	AA 127–818 similar to lactadherin; Domains: CBM_14/CHIT_BIND_II	S < I	26	0.21
	*Crassostrea gigas*						
**227**	D3BGG3	8.1e-5	23.9%	Similar to Zipper-like Domains-containing protein	S < I	14	0.20
	*Polysphondylium pallidum*						
**691**	A5Z1D6	1.4e-89	43.3%	Similar to epiphragmin; Domains: Fibr_C; SP	S < I	31	0.20
	*Cernuella virgata*						
**2357**	None	-	-	AA 395–528 similar to Domains: LDL_recept_a; (15% T); SP	S,I	12	0.19
572*	IFEA	1.7e-118	96.9%	Non-neuronal cytoplasmic intermediate filament protein	S < I	32	0.19
	*Helix aspersa*						
**7323**	A7RQD5	6.2e-41	32.2%	SP	S < I	25	0.19
	*Nematostella vectensis*						
**3883**	K1Q9V3	5.9e-190	86.2%	V-type proton ATPase catalytic subunit A	S < I	27	0.17
	*Crassostrea gigas*						
943*	G0ZGZ8	5.2e-38	97.8%	Actin	S < I	3	0.16
	*Metaphire posthuma*						
**169764**	R7VB66	3.0e-31	54.5%	SP	S < I	6	0.16
	*Capitella telata*						
**104312**	R7TB34	2.4e-5	45.5%	Similar to α-carbonic anhydrase; Domains: α-CA_2	S,I	4	0.14
	*Capitella telata*						
**196388**	None	-	-	(12% A, 11% G, 11% P, 14% S);	S < I	1	0.14
**2858**	H2ZUY5	5.5e-41	44.6%	Similar to adipocyte plasma membrane-associated protein; Domains: TolB_like/strictosidine:synthase_related	S < I	11	0.14
	*Latimeria chalumnae*						
**248122**	C3Z1I6	6.1e-15	38.8%	Similar to chitinase; Domains: glyco_hydro_18/chitinase	S < I	4	0.13
	*Branchiostoma floridae*						
**7807**	None	-	-	Domains: WAP; (12% A, 11% Q); SP	S < I	15	0.12
**1237**	K1QI28	1.9e-191	89.8%	V-type proton ATPase subunit B	S < I	18	0.11
	*Crassostrea gigas*						
**20308**	Q2LZN0	1.6e-9	27.0%	AA 49–467 similar to Dpse/GA10422/alkaline phosphatase; Domains: alkaline phosphatase; SP	S < I	14	0.11
	*Drosophila pseudoobscura*						
**20360**	H9K6W1	3.9e-37	37.5%	Similar to cadherin; Domains: cadherin; see also contig_75801	S < I	8	0.11
	*Apis mellifera*						
**31170**	*K7S1Q8*	3.2e-37	35.0%	Domains: CMB_14/CHIT_BIND_II; SP	S < I	17	0.11
	*Propionibacterium acidipropionici*						

All proteins listed here were found in all pigment fractions (yellow, orange and dark brown) with the exception of isotig_169764 which was only found in yellow and orange fractions. Proteins and peptides are listed in order of abundance expressed as a percentage of the shell total proteome that we can identify.

^1^The percentages of particularly abundant amino acid residues are given in brackets. “SP” indicates the presence of a signal peptide, “TM” indicates a likely trans-membrane protein.

The overall composition of the *C. nemoralis* shell proteome is dominated by uncharacterized and/or novel proteins (Table 1). Indeed the four most abundant proteins of the identifiable *C. nemoralis* proteome (constituting >52% of the total shell proteome) did not share significant similarity with any entries in UniProt (Table 1). Furthermore, 31 out of the 59 distinct proteins (52.5% of all proteins which, by abundance, account for 80% of the identifiable proteome) did not return hits against UniProt. This largely unique proteome reflects the situation for the majority of previously reported molluscan shell forming proteomes, and highlights the need for the development of reliable and repeatable *in vivo* functional assays in these systems. The most abundant protein in the *C. nemoralis* shell, accounting for more than 25% of the total identifiable proteinaceous material, was isotig_123 (Table 1). This Gly- and Pro-rich sequence did not share any BLASTp similarity with proteins in UniProt or Refseq, and no domains could be identified by Pfam or HMM searches. The two most abundant *C. nemoralis* shell proteins to share similarity with other proteins were isotig_2668 and isotig_821 (Table 1). *C. nemoralis* isotig_2668 (accounting for 3.87% of the shell proteome) shares significant similarity with a human poly-domain protein named SEL-OB/SVEP1. This human protein is present in osteogenic tissues and plays roles in cell adhesion [28]. Isotig_821 possesses a chitin-binding peritrophin-A domain, and shares similarity with several other molluscan proteins including BSMP (Blue Mussel Shell Protein) from *P. vulgata* and a large multi-domain containing protein from *C. gigas* (Sushi, von Willebrand, EGF and chitin-binding domains). Conspicuous in their abundance were several other *C. nemoralis* proteins that also possessed other recognizable chitin-interacting domains (14003, 1323, 101824, 84589, 63304, 248122 and 31170), suggesting that chitin is an important organic component of the *C. nemoralis* shell.

Many of the proteins identified in our proteomic analysis possess unusually high proportions of certain amino acids. These types of proteins are often found in molluscan biominerals [5,29,30]. For example, Lustrin (a protein isolated from the nacreous layer of the Californian red abalone *Haliotis rufescens*) contains a 272-residue domain rich in Gly (31%) and Ser (61%) residues. This domain has been suggested to act as an extensor molecule and to impart fracture resistance to the abalone shell [10]. However, it must pointed out that this function is yet to be experimentally verified. While we cannot assign functions to any of the various *C. nemoralis* proteins that contain domains rich in amino acids such as Gln, Asp, Pro, Gly and Met, their abundance and diversity in this dataset hints at the important role they must play in shell formation. Interestingly, one of these proteins (isotig_7807), which consists of 13% Ala and 11% Gln, also contains a Whey Acidic Protein (WAP domain), which is also present in the abalone Lustrin proteins [10,31]. WAP domains are thought to possess protease inhibitor activity due to the presence of 4-disulphide core (4-DSC) residues, which serine protease inhibitors also possess [32]. A diversity of protease inhibitor domain-containing proteins has been observed in other molluscan shell forming proteomes [6,29,33]. The presence of protease inhibitors in an external structure such as the molluscan shell may provide the shell with an ability to resist the digestive enzymes secreted by fouling organisms and predators that would dissolve and bore through the shell, for example natacid gastropods [34] polydorid annelids [35] and sponges [36].

We also detected a neurofilament protein (isotig_572) present in the *C. nemoralis* shell. While this may at first be considered a non-biomineral associated contaminant, a similar protein was reported from the shells of *Helix aspersa*[12], and we also note the presence of such a protein in the shell proteome of *L. gigantea* (see below). The presence of such presumably intra-cellular proteins in extra-cellular structures such as the molluscan shell are difficult to reconcile with our current understanding of how such biominerals are formed, and serve to highlight how far we are from a complete understanding of these processes. While a conventional model of shell formation would account for the presence of such proteins through the non-specific occlusion of cells and cellular debris into a growing face of a biomineral, there are alternative models that should perhaps be considered. The biophysical properties of filament proteins have been well studied, and they are known to be able to reversibly deform to several times their own length [37-39]. The fracture resistance properties of the molluscan shell, which exceeds that of pure CaCO_3_ by several orders of magnitude, is imparted to the biomineral by the organic components of the shell. A non-canonical secretory pathway for filament proteins, or the specific integration of filament-rich cells into the growing shell may be a mechanism by which the shell acquires such biomechanical properties. However, such hypotheses require further experimental investigation.

A previously described molluscan shell matrix protein, dermatopontin, was suggested to be the major proteinaceous component of the shell of the freshwater snail *Biomphalaria glabrata*[40,41]. Dermatopontin was also reported from the shells of other gastropods [42], and bivalves, where it is thought to play a role in nacre formation [43], and can also be found in taxa ranging from bacteria to humans. Interestingly, we did not detect Dermatopontin in the shell of *C. nemoralis*. To investigate this further we constructed an HMM profile of molluscan Dermatopontin proteins and used a local installation of HMMsearch [44] to query this against our translated *C. nemoralis* transcriptome. This search returned two significant hits (contig_46837 e-value 6.8e-27; contig_162693 e-value 5e-06). These contigs contain clear Dermatopontin domains, with contig_46837 also possessing a signal sequence. This discrepancy between the presence of Dermatopontin transcripts in the *C. nemoralis* mantle transcriptome, and the absence of Dermatopontin proteins in the shell-proteome may be a technical artifact, or a biological reality. While the diverse technical challenges of working with samples such as biominerals make the first possibility likely, the second scenario should also be considered in light of the apparent evolvability of molluscan shell forming secretomes [4,29,33]. Shell-forming genes under recent negative selection pressures could conceivably still be transcribed, but not translated or actively involved in shell formation. However, such a scenario requires additional investigation.

While a high proportion of the 59 proteins identified in the shell of *C. nemoralis* did not share any BLAST similarity with proteins in UniProt, some of them did contain domains that could be recognized by HMM searches. These are indicated in column 5 of Table 1. Many of these were hits were against “uncharacterized” domains or proteins of unknown function. In some cases trans-membrane (TM) regions could be identified. Such a finding is interesting as it leads to the question of how could such membrane-embedded proteins be located within the mature biomineral. Such a finding was also recently reported in a dataset of coral biomineralizing proteins [45] and several other studies [46,47]*.* In the coral study of Ramos-Silva et al. the majority of the MS peptides identified in the biomineral could only be matched against the extra-cellular regions of trans-membrane proteins, suggesting that these extra-cellular domains are specifically cleaved from the trans-membrane portions of TM proteins. We observe a similar phenomenon in our *C. nemoralis* data. Seventy-two peptides were observed in the MS data for isotig_5087, of these 70 were located in the putative extra-cellular domain of the protein (Additional file 6). The role that trans-membrane proteins play in molluscan shell-formation has thus far received little attention.

### Pigmentation of the C. nemoralis shell is not directly associated with a proteinaceous component

Previously, one of us described the Sometsuke protein from the shell of *Haliotis asinina*[4]. This protein is most likely coupled to a chromophore, which is involved in imparting both the red, and blue colors to the juvenile abalone shell (Figure 2A), and is perhaps the currently best understood molluscan shell-pigmentation mechanism at the genetic level. One of our primary motivations for the current work was to determine whether *C. nemoralis* also uses a protein-associated pigmentation mechanism to pattern its shell, and if so, to identify those proteins. Multiple protein extractions from a variety of *C. nemoralis* shells, including protocols without the potentially destructive washing with hypochlorite, suggested that this was not the case. Pigmented LDS-PAGE bands (as *per* Sometsuke from *H. asinina*) were never observed (Figure 2B). A dark brown material, which consistently accompanied extractions from dark brown shell fractions, remained predominantly in the PAGE sample buffer-insoluble material that was removed by centrifugation before electrophoresis in order to obtain clear and comparable LDS-PAGE electropherograms. While it could be argued that an insoluble *C. nemoralis* pigment-associated protein would need to be rendered soluble in order for it to be visualized on a gel, we point out that the denaturation treatments applied to the *C. nemoralis* samples (70°C for 10 minutes in detergent-containing loading buffer with mercaptoethanol) were more than adequate to solubilize the water-insoluble Sometsuke protein (Figure 2A).

Unfortunately we were unable to relatively quantify the proteins associated with each of the three pigment classes using a MaxQuant-implementation of label-free quantitation (LFQ) due to the significantly different solubility behavior of the protein extracts (see below). However, a second line of evidence suggests that *C. nemoralis* shell pigments are not associated with proteins. A qualitative assessment of the 59 proteins extracted from the shell (derived from three different pigment fractions) reveals that 58 were present in all three pigment fractions. These 58 proteins (which passed our stringent quality filters) account for >93% of the identifiable proteome. If we assume that a *C. nemoralis* pigment-associated protein would be at least moderately differentially abundant between the three pigment fractions (see Figure 1D, and as is certainly the case for Sometsuke in *H. asinina*, see [4], such a protein should either be easily observable on SDS/LDS-PAGE gels, or qualitatively differentially distributed across the three pigment fractions. The only protein to be qualitatively differentially distributed across the three pigment fractions was isotig_169764. This sequence was only detected in yellow and orange fractions, and is apparently highly conserved as it shares significant similarity with proteins in organisms ranging from bacteria (10e-23) and green algae (2e-05) to hemichordates (7e-34) and segmented worms (6e-46). Despite this conservation, there are no recognizable functional domains in the *C. nemoralis* orthologue of this protein. It was also relatively rare at just 0.16% of the total identifiable proteome, suggesting that it is unlikely to be directly involved in pigmenting the shell. Considering all of these points, our favored interpretation is that *C. nemoralis* shell pigments are not associated with proteins, and most likely have no homology with the Sometsuke pigmentation mechanism employed by *H. asinina*. If correct, this indicates that molluscan shell pigmentation mechanisms may have multiple evolutionary origins. However this scenario requires further investigation and experimental evidence.

**Figure 3:**
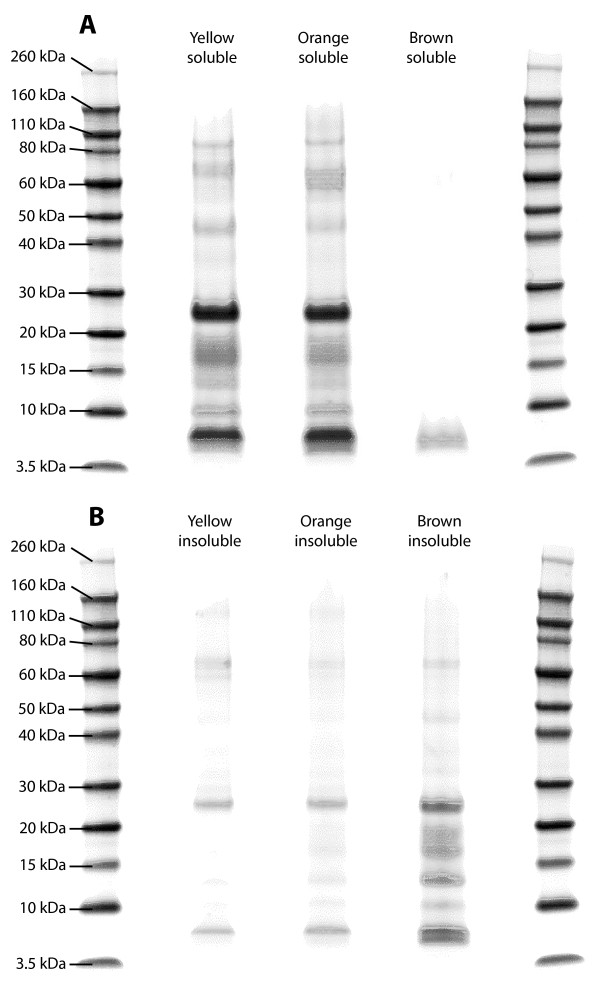
**Representative LDS-PAGE analyses of soluble and insoluble proteins derived from three different *C. nemoralis *shell-pigment fractions (yellow, orange and brown). A**. Soluble proteins. **B**. Insoluble proteins. The most striking difference between these fractions was the abundance of the proteins present in the yellow-soluble and orange-soluble fractions relative to the brown-soluble fraction. This pattern is reversed in the acid-insoluble fractions.

The LDS-PAGE profiles of acid-soluble vs. acid-insoluble proteins isolated from the three different *C. nemoralis* shell-pigment fractions differed significantly (Figure 3). Essentially, the profiles of soluble proteins derived from Yellow and Orange factions were similar to each other, and were in general more abundant and heterogeneous than the soluble proteins isolated from the Dark Brown fraction. In contrast, the acid-insoluble proteins isolated from the Dark Brown fraction were more abundant than those isolated from the Yellow or Orange fractions. Despite this difference, the prominent bands present in yellow-soluble, orange-soluble and brown-insoluble appear to be largely similar (Figure 3). While we cannot explain this observation, it is clear that the biochemical properties of the proteins present in the brown fraction are qualitatively different from those in the yellow and orange fractions. This may be the result of post-translational modifications that affect the solubility of each protein fraction, such as different degrees of cross-linking. However, this idea requires further investigation.

### Comparisons of molluscan shell forming proteomes

With the *C. nemoralis* shell proteome in hand we were able to conduct broad level comparisons against five other molluscan shell forming proteomes. Importantly, all six of these datasets are not transcriptome (RNA)-based datasets but are primarily composed of proteins that have been isolated from the shells of the respective species (mapped back to either RNASeq scale mantle transcriptomes or genome assemblies), and therefore are likely to be somehow directly involved in shell formation. To our knowledge, this is the first time such a proteome level comparison of molluscan shell forming proteins has been made.

**Figure 4:**
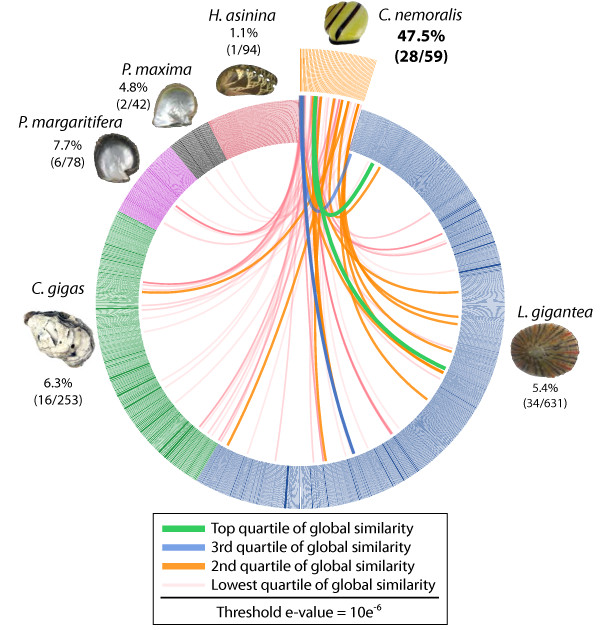
**BLASTp comparisons of the *C. nemoralis *shell proteome against the shell proteomes derived from 3 bivalves and 2 gastropods.** Individual lines spanning the ideogram connect proteins that share significant similarity (e values < 10e-6). Transparent red lines connect proteins with the lowest quartile of similarity (with a threshold of 10e-6) and green lines with the highest quartile of similarity. The percentage of each shell proteome that shared similarity with the *C. nemoralis* proteome is provided. Shell proteome datasets were derived from the following publications: *P. maxima* from [30]; *P. margaritifera* from [30]; *H. asinina* from [6] and [4]; *L. gigantea* from [33] and [5]; *C. gigas* from [7].

Of the 59 proteins we isolated from the *C. nemoralis* shell, 28 (47.5%) shared similarity (at an e-value threshold of 10e^−6^) with one or more proteins derived from the five other molluscan shell proteomes we investigated here (Figure 4). Interestingly only one *C. nemoralis* protein shared similarity with any of the 94*H. asinina* shell forming proteins. This single protein shares no significant similarity with any proteins in public databases and contains no identifiable conserved domains. It was previously reported that the shell forming proteome of *H. asinina* is highly divergent from other such molluscan proteomes, and that this could be interpreted as evidence of a rapidly evolving shell-forming secretome [4]. Given that *C. nemoralis* and *H. asinina* share more recent common ancestry than *C. nemoralis* does with any of the three bivalves investigated here (all three of which include more proteins with similarity to the *C. nemoralis* proteome) the result we report here appears to support that hypothesis.

Several *C. nemoralis* shell proteins displayed extremely high similarity with other molluscan shell forming proteins. The four *C. nemoralis* proteins to share the highest similarity with any other species were all shared with *L. gigantea*. In order of similarity these were: isotig_572 (a filament protein – see discussion above) at an e-value of 4e-143 (green link in Figure 4); isotig_84589 (a chitin-binding domain-containing protein – see below) at an e-value of 3e-93 (green link in Figure 4); isotig_63304 (another chitin-binding domain- containing protein) at an e-value of 2e-90 (blue link in Figure 4); and isotig_25891 (carbonic anhydrase) at an e-value of 6e-74 (blue link in Figure 4). In order to identify deeply conserved molluscan shell forming proteins, we ordered all *C. nemoralis* proteins that were found in any other shell proteome according to the number of databases they were found in (Additional file 7). This revealed two proteins that were found in four of the five molluscan shell proteomes: Cnem821 and Cnem248122. Cnem821 possesses a chitin-binding Periotrophin-A domain (Carbohydrate Binding Module 14: Pfam PF01607). The second protein found in four of the five molluscan shell proteomes (Cnem248122) shared significant similarity with chitinase proteins in Swissprot. These results emphasize the prominent role that chitin is likely to play in the construction of disparate molluscan shells and indeed in many metazoan biominerals [48-52].

Other conserved *C. nemoralis* shell forming proteins of interest include two carbonic anhydrase domain-containing proteins (Cnem25891 and Cnem104312) and two V-ATPase subunits (Cnem3883 and Cnem1237). V-ATPases have not previously been shown to play a role in molluscan shell formation (beyond their presence in mantle EST or RNASeq datasets), however it could be expected that their ability to transport H^+^ across membranes would afford them a central role in the regulation of shell formation. Indeed such proton pumps are known to play roles in the calcification of a variety of metazoan biominerals [53-55]. While one of the carbonic anhydrase domain-containing isotigs (Cnem104312) is clearly incomplete, the other (Cnem25891) is potentially complete and encodes a protein of 1,028 amino acids (the corresponding isotig contains 3,859 bp). This protein contains a signal sequence, a carbonic anhydrase domain with phylogenetic affinity to the secreted and membrane bound α-CAs (see Additional file 8 for a phylogenetic analysis) and a carboxyl region of relatively low complexity (Additional file 9). The shell-forming Nacrein proteins also contain carbonic anhydrase domains and have been previously described from gastropod and bivalve shells [56,57]. Interestingly the CA-domain in the *C. nemoralis* protein is not interrupted by the low-complexity region as it is in the Nacreins, and the *C. nemoralis* low complexity domain is composed of Gln residues rather than Gly and Asn residues (Additional file 9). The hydropathy profile of all of these proteins display similar characteristics (Additional file 10), and suggests that the low complexity domains interact with the water-soluble phases of the biomineralization processes. Miyamoto et al. [58] reported that a recombinant Nacrein protein inhibited the precipitation of CaCO_3_ in *in vitro* calcification assays, and that removal of the repetitive low-complexity domain attenuated this inhibitory activity. While the results of such *in vitro* calcification assays should always be interpreted with caution, this result indicates that the low complexity domains of molluscan shell forming α-CAs have a significant impact on the activity of the enzyme to which they are fused or inserted. The fact that the α-CA we have identified here has a significantly different domain of low-complexity from previously described shell-associated α-CAs suggests that this gene may have a different evolutionary history.

## Conclusions

The 59 shell-associated proteins we report here represent the largest collection of proteins from a pulmonate shell to date. The abundant proteins in this dataset either display no similarity to known proteins, or similarity to uncharacterized proteins. Comparisons of this dataset with other molluscan shell-forming proteomes indicate that almost half of the *C. nemoralis* shell-forming proteome we describe here shares similarity with the shell-forming components of other molluscs. Two lines of experimental evidence failed to identify the presence of pigment-associated proteins in the *C. nemoralis* shell. Considering the clear association between a protein and a pigment in the juvenile *Haliotis* shell, this finding indicates that molluscan shell pigmentation mechanisms may have diverse evolutionary origins. This dataset will serve as an important platform from which further studies aimed at the characterization of pulmonate shell forming and pigmenting genes can be performed.

## Methods

### *Generation of a reference* C. nemoralis mantle *transcriptome for proteomic surveys*

Seven total RNA extractions derived from the distal-most edge (i.e. the shell forming region) of the mantle tissue of 4 juvenile *C. nemoralis* individuals (two extractions from each of three individuals for Illumina sequencing and one extraction from a fourth individual for 454 sequencing) was extracted using Trizol according to the manufacturer’s instructions. These total RNA extractions were sequenced on the Illumina HiSeq2000 and Roche 454 platforms and assembled using the CLC Genomics workbench (version 5.5.2). This assembly generated a total of 676,358 contigs >100 bp, summing to a total of 193,892,905 bp (max contig size = 14,765 bp, median contig size = 180 bp). In order to reduce the redundancy of this assembly for proteomic interrogation (see below) the following steps were applied. First, contigs shorter than 500 bp were removed. All remaining contigs were then clustered into isotigs using “usearch” [59]. The longest possible open reading frame (which was required to be >50 amino acids) was then extracted from each isotig using standard Perl scripts. These translated ORFs were then clustered again using “usearch” to produce a total of 55,623 putative coding translated fragments. This dataset was used to conduct the proteomic surveys (see below). All assembled nucleic acid sequences discussed in this work are available in Additional file 11.

### Preparation of shell matrix and peptides

Shells from approximately 100 freshly collected snails were crushed into approximately 5 mm^2^ fragments and carefully sorted into three populations: dark brown (20.5 g derived from shells with a yellow background); yellow (23.7 g); and orange (12.3 g; see Figure 1). The shell pieces were either briefly washed with deionized water, or with sodium hypochlorite (6-14% active chlorine; Merck, Darmstadt, Germany) for 2 h at room temperature with 5 min sonication and change of hypochlorite solution every 30 min. Hypochlorite-treated shells were then washed with deionized water and dried. Washed shell pieces were demineralized in 50% acetic acid (20 mL/g of shell) for 16 h at 4°C. The resulting suspensions were dialyzed successively against 3 × 10vol 10% acetic acid and 2 × 10vol 5% acetic acid at 4-6°C (Spectra/Por 6 dialysis membrane, mw cut-off 2000; Spectrum Europe, Breda, The Netherlands). The dialyzed suspensions were centrifuged for 1 h at 4°C and 12000g_av._ Pellets and supernatants were lyophilized and analyzed separately.

LDS-PAGE was performed with pre-cast 4-12% gradient Novex Bis-Tris gels in MES buffer using reagents and protocols supplied by the manufacturer (Invitrogen, Carlsbad, CA). The sample buffer was complemented with β-mercaptoethanol to a final concentration of 1% and samples were heated to 70°C for 10 min. Sample buffer-insoluble material was removed by centrifugation at 16000 g for 5 min. Gels were loaded with the soluble fraction at 200 μg matrix/lane.

Reduction, carbamidomethylation and enzymatic cleavage of matrix proteins were performed using a modification of the FASP (Filter-aided sample preparation) method [60]. Aliquots of 200 μg of acid-soluble or acid-insoluble shell matrix were suspended in 200 μl of 0.1 M Tris, pH8, containing 6 M guanidine hydrochloride and 0.01 M dithiothreitol (DTT). This mixture was heated to 56°C for 60 min, cooled to room temperature, and centrifuged at 13000 rpm in an Eppendorf bench top centrifuge 5415D for 15 min. The supernatant was loaded into an Amicon Ultra 0.5 ml 30 K filter device (Millipore; Tullagreen, Ireland). DTT was removed by centrifugation at 13000 rpm for 15 min and washing with 2 × 1vol of the same buffer. Carbamidomethylation was done in the device using 0.1 M Tris buffer, pH8, containing 6 M-guanidine hydrochloride and 0.05 mM iodoacetamide and incubation for 45 min in the dark. Carbamidomethylated proteins were washed with 0.05 M ammonium hydrogen carbonate buffer, pH8, containing 6 M urea, and centrifugation as before. Each sample was then incubated with 2 μg of Lysyl endopeptidase (WAKO Chemicals GmbH, Neuss, Germany) in 40 μl of 0.05 mM ammonium hydrogen carbonate buffer containing 6 M urea for 6 h at 37°C. This was followed by addition of 4 μg of trypsin (Sequencing grade, modified; Promega, Madison, USA) in 80 μl of 0.05 M ammonium hydrogen carbonate buffer and further incubation at 37°C for 16 h. Peptides were collected by centrifugation and the filters were washed twice with 40 μl of 0.05 M ammonium hydrogen carbonate buffer. The peptide solutions were acidified to pH ~ 1 with trifluoroacetic acid and desalted for mass spectrometric analysis with C18 Stage Tips [61].

### LC-MS analysis

Peptide mixtures were analyzed by on-line nanoflow liquid chromatography using the EASY-nLC 1000 system (Proxeon Biosystems, Odense, Denmark, now part of Thermo Fisher Scientific) with 20 cm capillary columns of an internal diameter of 75 μm filled with 1.8 μm Reprosil-Pur C18-AQ resin (Dr. Maisch GmbH, Ammerbuch-Entringen, Germany). Peptides were eluted with a linear gradient from 5-30% buffer B (80% acetonitrile in 0.1% formic acid) for 100 min, 30-60% B for 12 min and 80-95% B for 8 min at a flow rate of 250 nl/min. The eluate was electro-sprayed into an Orbitrap Elite (Thermo Fisher Scientific, Bremen, Germany) using a Proxeon nanoelectrospray ion source. The Orbitrap Elite was operated essentially as previously described [62] in a HCD top 10 mode with dynamic selection of the 10 most intense peaks of each survey scan (300-1750Th) for fragmentation. The resolution was 120,000 for full scans and 15,000 for fragments (both specified at m/z 400). Ion target values were 1e6 and 5e4ms, respectively. Dynamic exclusion time was 30 sec.

### Analysis of proteomic data

Raw files were processed using the Andromeda search engine-based version 1.3.9.21 of MaxQuant (http://www.maxquant.org/) with “second peptide” enabled, iBAQ (intensity-based absolute quantitation; [63] as implemented in recent versions of MaxQuant, and “match between runs” options (match time window 0.5 min; alignment time window 20 min) [64-66]. For protein identification the *C. nemoralis* mantle transcriptome database (see above) was converted into a FASTA-formatted protein sequence database, downloaded into MaxQuant, and automatically combined with the reversed sequences and sequences of common contaminants, such as human keratins. Carbamidomethylation was set as fixed modification. Variable modifications were oxidation (M), N-acetyl (protein), pyro-Glu/Gln (N-term) and phospho (STY). The initial mass tolerance for full scans was 7 ppm and 20 ppm for MS/MS. Two missed cleavages were allowed and the minimal length required for a peptide was seven amino acids. The peptide and protein false discovery rates (FDR) were set to 0.01. The maximal posterior error probability (PEP), which is the individual probability of each peptide to be a false hit considering identification score and peptide length, was set to 0.01. The minimal peptide score was 50. Two sequence-unique peptides were required to occur at least three times in two different replicates for high-confidence protein identifications. In exceptional cases single-sequence-unique identifications were accepted if the peptide occurred in at least three different replicates and was identified in both hypochlorite washed samples and water washed samples.

Identifications with one or two sequence-unique peptides were routinely validated using the MaxQuant Expert System software [67] considering the assignment of major peaks, occurrence of uninterrupted y- or b-ion series of at least four consecutive amino acids, preferred cleavages N-terminal to proline bonds, the possible presence of a2/b2 ion pairs and immonium ions, mass accuracy and score. Based on the sum of peak intensities, the iBAQ [63] option of MaxQuant was used to calculate the approximate proportion of each protein in the total identifiable proteome.

Sequence similarity searches were performed with FASTA (http://www.ebi.ac.uk/Tools/sss/fasta/) [68] against current releases of the Uniprot Knowledgebase (UniProtKB). Other bioinformatics tools used were Clustal Omega for sequence alignments (http://www.ebi.ac.uk/Tools/msa/clustalo/) [69], InterProScan (http://www.ebi.ac.uk/Tools/pfa/iprscan/) [70] for domain predictions, and SignalP 4.1 (http://www.cbs.dtu.dk/services/SignalP/) [71] for signal sequence prediction.

### Comparisons of molluscan shell forming proteomes

BLASTp based comparisons of the *C. nemoralis* shell proteome were made against five previously published molluscan shell proteomes. These included 42 *Pinctada maxima* proteins reported by Marie et al. [30], 78 *Pinctada margaritifera* proteins reported by Marie et al. [30], 253 *Crassostrea gigas* proteins reported by Zhang et al. [7], a combined set of 94 *Haliotis asinina* proteins reported by Marie et al. [6] and Jackson et al. [4], and a combined set of 631 *Lottia gigantea* proteins reported by Marie et al. [33] and Mann et al. [5]. The e-value threshold was set to 1e-06. These comparisons were made using a modified version of Circoletto [72] which uses an implementation of the legacy BLAST package. The following command was issued to the circoletto.pl script: circoletto.pl --query XX --database XX --untangling_off --e_value 10e-6 --best_hit_type local --score2colour eval. The *.conf files generated by circoletto.pl were modified using custom Perl scripts and then passed to Circos [73] in order to generate an ideogram. The Circoletto *.blasted file which details all of the BLASTp results is provided as Additional file 12.

## Competing interests

The authors declare that they have no competing interests.

## Authors’ contributions

DJJ carried out the RNA extractions for RNA-Seq and collected and sorted *C. nemoralis* shell material for proteome work. KM carried out the protein extractions and conducted the LC-MS analyses. Both authors conducted the bioinformatic analyses, and drafted the manuscript. Both authors read and approved the final manuscript.

## Supplementary Material

Additional file 1 A list of all accepted identifications after manual validation of all protein/protein group identifications provided by MaxQuant. Click here for file

Additional file 2 **A complete list of protein identifications in matrices extracted from shells not treated with hypochlorite plus ultra-sonication.** Click here for file

Additional file 3 **A complete list of protein identifications in matrices extracted from shells treated with hypochlorite plus ultra-sonication before demineralization in acid.** Click here for file

Additional file 4 **The peptide data complementing protein data of Additional file****2****.** Click here for file

Additional file 5 **The peptide data complementing protein data of Additional file****3****.** Click here for file

Additional file 6 **A schematic representation of a *C. nemoralis *** **putative trans-membrane protein (derived from isotig_5087), onto which the spatial distribution of the 72 LC-MS peptides are mapped.** Click here for file

Additional file 7 **Top BLASTp hits returned against *C. nemoralis *** **queries from five molluscan shell proteomes and Swissprot.** Click here for file

Additional file 8 A 50% majority rule consensus tree generated by Bayesian methods representing the phylogenetic relationships of metazoan CAs. Click here for file

Additional file 9 The Nucleotide and derived protein sequence of Cnem25891. Click here for file

Additional file 10 **Hydropathy profiles of Cnem25891 and two previously reported molluscan shell-forming proteins which also posses CA domains.** Click here for file

Additional file 11 Contains the 59 isotigs (nucleic acid sequences) that were recovered by MaxQuant. Click here for file

Additional file 12 The Circoletto generated “*.blasted” file used to generate the Circos figure (Figure 4**).** Click here for file
