# Supplementary material for: Characterization of the pigmented shell-forming proteome of the common grove snail Cepaea nemoralis
Source: BMC Genomics. 2014 Mar 31;15:249. doi: 10.1186/1471-2164-15-249 (PMC4023409; doi:10.1186/1471-2164-15-249)
Supplement: Additional file 1 — A list of all accepted identifications after manual validation of all protein/protein group identifications provided by MaxQuant. [file 1471-2164-15-249-S1.pdf]

Additional File 1

The *Cepaea nemoralis* shell proteome - accepted identifications.

Contig	Similar to (best match)	FASTA E-value	Iden-tity	Protein		Shell color & Fraction	Unique + razor peptides	% of total (iBAQ)
**10054**				Uncharacterized; 16% Q, 10% S	**-H**	**Y,O**; S<I	2	0.0003
					**+H**	**Y,O**; S,I	3	0.0014
**100974**				Uncharacterized; 10% R, 10% N, 13% Q; signal peptide	**-H**	**Y,O,B**; S<I	2	0.0088
					**+H**	**Y,O,B**; S<I	2	0.0287
**10133**	GRP78_APLCA (aa86-544)	8.2e-160	90.8%	78kDa glucose-regulated protein; domains: HSP70; shares peptide with contig_695; Lotgi1|216416 (0e0)	**-H**	**Y,O,B**; S<I	7	0.0102
					**+H**	**Y,O,B**; S<I	11	0.0184
**10182**	A6N8F4_HALAI (aa1-474)	1.2e-101	85.7%	HSP90A; domains: HSP90A Lotgi1|161608 (0e0)	**-H**	**Y,O,B**; S<I	9	0.0098
					**+H**	**Y,O,B**; S<I	12	0.0134
**101824**	K1QJK2_CRAGI (aa1063-1449)	2.1e-22	29.4%	Uncharacterized; domains: CBM_14/ CHIT_BIND_II; shares peptide with contig_14003 Lotgi1|231869 (2.3e-70); see also contig_84589!	**-H**	**Y,O,B**; S<I	19	0.0573
					**+H**	**Y,O,B**; S<I	29	0.3066
**1021**				Uncharacterized; signal peptide	**-H**	**Y,O,B**; S>I	2	0.0082
					**+H**	**-**	-	-
**102484**	K1RCY7_CRAGI	5.7e-104	63.9%	Eukaryotic peptide chain release factor GTP-binding subunit ERF3B; Lotgi1|180287 (0e0)	**-H**	**-**	-	-
					**+H**	**Y**; I	2	0.0001
**102677**	Q9NL16_BRABE (aa147-453)	3.3e-22	29,1%	Similar to AmPTP10 protein (Fragment); domain: protein_tyrosine_phosphatase; Lotgi1|181300 (7.6e-26)	**-H**	**-**	-	-
					**+H**	**Y,O**; I	2	0.0008

	G3FPE5_HELLU			Hemocyanin β-subunit				
**11358**	(aa1-431)	1.0e-186	92.1%		**-H**	**Y,O,B**; I	7	0.0137
**1031**	(aa611-1371)	0e0	93.3%			**Y,O,B**; S<I	17	0.0159
**2283**	(aa1365-2093)	0e0	94.9%			**Y,O,B**; S<I	10	0.0105
					**+H**	**Y,O,B**; S<I	11	0.0167
						**Y,O,B**; I	16	0.0133
						**Y,O,B**; I	10	0.0054
**104312**	R7TB34_9ANNE (aa221-273)	2.4e-5	45.5%	Similar to α-carbonic anhydrase; domain: α-CA_2	**-H**	**Y,O,B**; S<I	3	0.0379
					**+H**	**Y,O,B**; S,I	4	0.1382
	K1R401_CRAGI			Spectrin α-chain; Lotgi1|202396 (1.1e-167)	**-H**	**-**	-	-
**10507**	(aa785-1076)	2.6e-88	86.2%		**+H**	**Y,B**; I	2	0.0004
**112929**	(aa171-720)	4.2e-136	74.4%			**Y,O**; I	4	0.0007
**105370**	J7HZQ3_POMCA (aa83-275)	2.7e-39	60.6%	Apoptosis-inducing factor 3-like protein; Lotgi1|214061 (1.7e-63)	**-H**	**-**	-	-
					**+H**	**Y,O**; I	6	0.0125
**10584**				Uncharacterized protein; 16% D, 10% E	**-H**	**Y,O,B**; S<I	12	0.4573
					**+H**	**Y,O,B**; S<I	14	1.3578
**10656**	R7TYY9_9ANNE (aa1-1757)	0e0	64.7%	Uncharacterized/similar to NADPH-dependent glutamate synthase; Lotgi1|133427 (0e0)	**-H**	**-**	-	-
					**+H**	**Y**; I	2	<0.0001
	A6YM37_9EUPU			Similar to matrilin-like 40kDa;				
**10722**	(aa194-388)	5.7e-34	48.7%	domains: VWA; Lotgi1|234764 (3.8e-16);	**-H**	**Y,O,B**; S<I	7	0.0709
**2338**	(aa17-147)	1.5e-13	31.3%	EGF, signal peptide; Lotgi1|133178 (1.9e-8)		**Y,O,B**; I	3	0.0278
**619**	(aa201-348)	1.6e-17	40.5%	Lotgi1|122049 (1.4e-18), several proteins?		**Y,O,B**; I	5	0.0130
					**+H**	**Y,O,B**; S<I	6	0.0829
						**Y,O,B**; S<I	3	0.0302
						**Y,O**; I	4	0.0051
**107621**				Uncharacterized; 11% P, 18% S, 19% T; Lotgi1|163383 (1.2e-25)	**-H**	**Y,O,B**; S	2	0.0027
					**+H**	**B**; S	2	0.0005

**108124**	G7YE09_CLOSI (aa157-271)	0.0019	27.6%	aa157-272 similar to fasciclin-1; domains: FAS1, signal pept; Lotgi1|237218 (1.4e-21)	**-H**	**Y,O,B**; S<I	2	0.0092
					**+H**	**Y,B**; S<I	5	0.0278
**1089**	R7VGL7_9ANNE<br>Q2HZD8_PINFU	2.0e-155<br>2.1e-153	81.7%<br>81.1%	ATP synthase subunit β; Lotgi1|201878 (0e0)	**-H**	**Y,O,B**; S<I	19	0.0459
					**+H**	**Y,O,B**; S<I	20	0.0679
**109239**	KCY_BOVIN	3.2e-45	63.6%	UMP-CMP kinase; Lotgi1|149165 (1.3e-83)	**-H**	**-**	-	-
					**+H**	**Y,O**; I	3	0.0028
**222793**<br>**109421**<br>**110180**	GNAI_LYMST<br>GNAO_LYMST<br>GNAS_LYMST	1.5e-146<br>1.5e-146<br>5.5e-156	97.7%<br>89.5%<br>94.1%	Guanine nucleotide-binding protein G(i) subunit α<br>Guanine nucleotide-binding protein G(o) su α<br>Guanine nucleotide-binding protein G(s) subunit α; Lotgi1|226384 (0e0)	**-H**	**Y,O**; I	1	0.0003
					**+H**	**Y,O**; I	3	0.0018
**10986**	K1PPK3_CRAGI (aa339-543)	4.9e-24	37.6%	Fibropellin-1; domains: CHIT_BIND_II; Lotgi1|234405 (1.3e-31)	**-H**	**Y,B**; S<I	5	0.0143
					**+H**	**Y,B**; S,I	2	0.0031
**110158**				Uncharacterized; 20% P, 18% S	**-H**	**Y,O,B**; S>I	3	0.0128
					**+H**	**Y,O,B**; S	2	0.0107
**110175**	K1QLH8_CRAGI (aa600-767)	2.5e-28	43.8%	Peroxidasin; Lotgi1|163527 (1.2e-34)	**-H**	**O,B**; I	2	0.0015
					**+H**	-	-	-
**110398**	E0W375_PEDHC	6.5e-55	30.4%	Uncharacterized/similar to cadherin-23; domains: cadherin, signal pept; Lotgi1|229248 (4.1e-116)	**-H**	**Y,O,B**; I	3	0.0012
					**+H**	**Y,O,B**; I	7	0.0047
**1108**	K1QX01_CRAGI	0.005	29.5%	Uncharacterized; domain: EFh; 13% D; Lotgi1|238177 (1.7e-10)	**-H**	**B**; I	4	0.0099
					**+H**	**B**; I	2	0.0024
**11108**	Q45RT7_BIOGL (aa209-455)	1.6e-61	79.8%	Intermediate filament protein; domain: IF; Lotgi1|204921 (7.5e-71)	**-H**	**Y,O,B**; I	2	0.0024
					**+H**	**Y,O,B**; I	4	0.0679
**11180**	B7Q2Q7_IXOSC (aa5-764)	1.4e-110	39.7%	Receptor tyrosine phosphatase type r2a, putative; domains: IG, FN3; Lotgi1|128794 (0e0)	**-H**	**-**	-	-
					**+H**	**Y,O,B**; I	7	0.0039
**111860**	YCF2E_CAEEL (aa139-381)	1.1e-55	51.0%	Uncharacterized peptidase C1-like protein F26E4.3/similar to cathepsin B; domain: Peptidase_C1A; Lotgi1|118077 (3.0e-102)	**-H**	**-**	-	-
					**+H**	**Y,O**; I	2	0.0012

<table>
<tr><td>112077</td><td>Q9NL47_CIOIN
(aa216-388)</td><td>1.4e-10</td><td>38.2%</td><td>Cadherin; domains: cadherin;
Lotgi1|229248 (2.5e-13)</td><td>-H</td><td>Y,O,B; I</td><td>1</td><td>0.0009</td></tr>
<tr><td></td><td></td><td></td><td></td><td></td><td>+H</td><td>Y,O,B; S< I</td><td>1</td><td>0.0022</td></tr>
<tr><td>11304</td><td>G3PTN0_GASAC
(aa132-412)</td><td>2.8e-82</td><td>63.2%</td><td>β-hexosaminidase; shares 2 peptides with contig_35849; Lotgi1|120422 (3.3e-127)</td><td>-H</td><td>Y,O,B; I</td><td>1</td><td>0.0011</td></tr>
<tr><td></td><td></td><td></td><td></td><td></td><td>+H</td><td>Y,O;I</td><td>6</td><td>0.0080</td></tr>
<tr><td></td><td>Q3V653_9EUPU</td><td></td><td></td><td>Thioester-containing protein; domains: terpenoid cyclase/protein prenyltransferase, A2M/A2M_N_2; AeM_N, signal peptide
Lotgi1|209261 (3.3e-87)/162872 (1.2e-27)/233231 89.8e-31)</td><td></td><td></td><td></td><td></td></tr>
<tr><td>11338</td><td>(aa872-1245)</td><td>1.2e-120</td><td>73.8%</td><td></td><td>-H</td><td>Y,O; I</td><td>5</td><td>0.0060</td></tr>
<tr><td>21999</td><td>(aa264-567)</td><td>1.5e-45</td><td>66.5%</td><td></td><td></td><td>Y,O,B; I</td><td>9</td><td>0.0116</td></tr>
<tr><td>92485</td><td>(aa4-270)</td><td>1.2e-61</td><td>60.7%</td><td></td><td></td><td>Y,O,B; I</td><td>3</td><td>0.0045</td></tr>
<tr><td></td><td></td><td></td><td></td><td></td><td>+H</td><td>Y,O; I</td><td>12</td><td>0.0298</td></tr>
<tr><td></td><td></td><td></td><td></td><td></td><td></td><td>Y,O,B; S< I</td><td>10</td><td>0.0277</td></tr>
<tr><td></td><td></td><td></td><td></td><td></td><td></td><td>Y,O,B; I</td><td>6</td><td>0.0209</td></tr>
<tr><td>1152</td><td></td><td></td><td></td><td>Uncharacterized; signal peptide; 16% G, 15% M, 10% S</td><td>-H</td><td>Y,O,B; S>I</td><td>3</td><td>18.0689</td></tr>
<tr><td></td><td></td><td></td><td></td><td></td><td>+H</td><td>Y,O,B; S>I</td><td>3</td><td>15.5491</td></tr>
<tr><td>116159</td><td>Q5MGE8_LONON
(aa172-333)</td><td>5.9e-44</td><td>61.6%</td><td>Cysteine peptidase 2 cathepsin-B-like; domain: peptidase_C1A; Lotgi1|197490 (1.0e-58)</td><td>-H</td><td>Y,O,B; S<I</td><td>1</td><td>0.0064</td></tr>
<tr><td></td><td></td><td></td><td></td><td></td><td>+H</td><td>Y,O,B; S<I</td><td>2</td><td>0.0252</td></tr>
<tr><td>116171</td><td>HBB_HUMAN</td><td>2.6e-65</td><td>100%</td><td>Hemoglobin β-subunit; shares 1 of 9 peptides with CON</td><td>-H</td><td>Y,O,B; S<I</td><td>9</td><td>0.0491</td></tr>
<tr><td></td><td></td><td></td><td></td><td></td><td>+H</td><td>-</td><td>-</td><td>-</td></tr>
<tr><td></td><td>K1QB61_CRAGI</td><td></td><td></td><td>Protocadherin Fat 4; domains: cadherin;
Lotgi1|154324 (3.3e-37/0e0)</td><td></td><td></td><td></td><td></td></tr>
<tr><td>116744</td><td>(aa4522-4789)</td><td>1.2e-29</td><td>38.1%</td><td></td><td>-H</td><td>Y,O,B; I</td><td>2</td><td>0.0061</td></tr>
<tr><td>25750</td><td>(aa2270-4179)</td><td>1.7e-143</td><td>33.4%</td><td></td><td></td><td>Y,O,B; I</td><td>22</td><td>0.0046</td></tr>
<tr><td></td><td></td><td></td><td></td><td></td><td>+H</td><td>Y,O,B; S<I</td><td>3</td><td>0.0252</td></tr>
<tr><td></td><td></td><td></td><td></td><td></td><td></td><td>Y,O,B; S<I</td><td>29</td><td>0.0001</td></tr>
<tr><td>11813</td><td>G2XK75_9BIVA</td><td>1.8e-33</td><td>56.3%</td><td>Glutathione-S transferase 1; domains: GST_N_3, GST_C; Lotgi1|191561 (4.4e-43)</td><td>-H</td><td>Y, I</td><td>1</td><td>0.0005</td></tr>
<tr><td></td><td></td><td></td><td></td><td></td><td>+H</td><td>Y; I</td><td>1</td><td>0.0007</td></tr>
</table>

**1188**	K1P9P0_CRAGI (aa131-296)	1.2e-27	47.0%	Mesenchyme-specific cell surface glycoprotein; domain:WD40/YVTN repeat-like;	**-H**	**Y,O,B**; S<I	8	0.2618
**4282**	(aa442-548)	4.4e-21	50.9%	Lotgi1|190352 (1.3e-38)/162322 (1.6e-26)		**Y,O,B**; S<I	6	0.3013
					**+H**	**Y,O,B**; S<I	9	0.5092
						**Y,O,B**; S<I	6	0.5577
**11883**	K1QSU9_CRAGI	1.6e-75	36.3%	Eosinophil peroxidase; domains: haem peroxidase, signal pept; Lotgi1|233510 (7.4e-85)	**-H**	**B**; I	5	0.0004
					**+H**	-	-	-
**11910**	G0WX16_TEGGR (aa217-366)	2.3e-47	77.3%	Glutamine synthetase; Lotgi1|230064 (8.0e-76)	**-H**	**-**	-	-
					**+H**	**B,Y**; I	2	0.0028
**11930**	B9M8J1_GEOSF	6.4e-13	31.5%	Uncharacterized; signal peptide; 11% G; Lotgi1|234762 (2.6e-22)	**-H**	**Y,O,B**; S,I	2	0.0318
					**+H**	**Y,B**; S,I	1	0.0060
**12039**	K1RW67_CRAGI (aa1978-2657)	1.7e-106	44.4%	Uncharacterized; domains: GPS, GPCR family, transmembrane; Lotgi1|164559 (1.6e-176)	**-H**	**Y,O**; I	6	0.0007
					**+H**	**Y,O**; S<I	9	0.0046
**120497**	I1G5E0_AMPQE	6.9e-27	51.5%	Uncharacterized; domains: EFh; 15% A, 12% G; Lotgi1|199248 (6.1e-70)	**-H**	**Y,O,B**; I	1	0.0179
					**+H**	**Y**; I	1	0.0021
**1208**	B2YHX3_9BILA	9.2e-54	87.7%	Similar to ribosomal protein S16; Lotgi1|98402 (2.7e-69)	**-H**	**Y,B**; I	2	0.0009
					**+H**	**Y,B**; I	2	0.0007
**1219**	B6RB63_HALDI	6.0e-117	62.0%	Protein disulfide isomerase; Lotgi1|184255 (3.1e-97)	**-H**	**Y,B,O**; I	12	0.0062
					**+H**	**Y,O,B**; S<I	17	0.0475
**12284**	B7Q505_IXOSC	8.3e-67	60.7%	Elongation factor Tu; Lotgi1|178160 (0e0)	**-H**	**-**	-	-
					**+H**	**Y; I**	2	0.0003
**12292**	A8UF56_ARENA	4.7e-40	66.7%	Ribosomal protein L14; Lotgi1|229862 (3.9e-45)	**-H**	**Y,O,B**; S<I	1	0.0023
					**+H**	**Y,O,B**; I	2	0.0021
**123**				Uncharacterized; 12% G, 18% P	**-H**	**Y,O,B**; S>I	13	41.2668
					**+H**	**Y,O,B**; S>I	15	26.2241

**123124**	K1R7R4_CRAGI	1.2e-57	38.8%	Multiple inositol polyphosphate phosphatase 1; signal peptide; Lotgi1|230275 (2.1e-128)	**-H**	**Y,O**; I	2	0.0008
					**+H**	**Y,O**; S<I	8	0.0062
**1237**	K1QI28_CRAGI	1.9e-191	89.8%	V-type proton ATPase subunit B; Lotgi1|178589 (0e0)	**-H**	**Y,O,B**; S<I	12	0.0167
					**+H**	**Y,O,B**; S<I	18	0.1087
**124132**	K1Q3Y3_CRAGI (aa35-318)	1.6e-32	34.9%	Blastula protease 10; domains: peptidase M12A/astacin, signal peptide; shares peptide with contig_7075; Lotgi1|109273 (2.2e-34)	**-H**	**Y,O,B**; S<I	3	0.0037
					**+H**	**Y,O**; S,I	4	0.0152
**12587**	Q9GQB4_NUCLP (aa105-292)	9.9e-58	80.3%	Malate dehydrogenase; Lotgi1|225558 (8.1e-84)	**-H**	**Y,O,B**; I	2	0.0062
					**+H**	**Y,O,B**; I	2	0.0025
**1265**				Uncharacterized; 12% G	**-H**	**Y,O,B**; S>I	12	3.8779
					**+H**	**Y,O,B**; S>I	9	4.3957
**126964**	K1P8K1_CRAGI	7.9e-34	33.2%	Meteorin-like protein; domain: meteorin_like, signal peptide; Lotgi1|156516 (5.2e-44); see also 159000	**-H**	**Y,O**; I	3	0.0008
					**+H**	**Y,O**; S<I	5	0.0039
**127437**				Uncharacterized; signal peptide	**-H**	**Y,O**; I	1	0.0004
					**+H**	**O**; S<I	3	0.0007
**1281**				Uncharacterized; signal peptide	**-H**	**-**	-	-
					**+H**	**O,B**; I	4	0.0021
	K1QSW2_CRAGI			Protein-glutamine gamma-glutamyltransferase K; domain: transglutaminase; Lotgi1|122446 (2.8e-85)/233545 (3.3e-76)	**-H**	**-**	-	-
**12872**	(aa219-389)	3.7e-55	63.2%		**+H**	**Y**; I	1	0.0016
**80303**	(aa2-209)	1.1e-46	49.5%			**Y,O**; I	8	0.0046
**12941**				Uncharacterized; 18% D, 14% L	**-H**	**Y,O,B**; S<I	9	0.4282
					**+H**	**Y,O,B**; S<I	12	1.3727

**130482**	K1Q4S5_CRAGI (aa1242-1494)	3.3e-27	38.5%	Similar to cadherin-87A; domains: cadherin Lotgi1|229249 (1.3e-43/3.1e-59)	**-H**	**Y,O**; I	1	0.0005
**70072**	(aa276-693)	7.3e-47	41.4%			**Y,O,B**; I	3	0.0024
					**+H**	**Y,O**; I	1	0.0011
						**Y,O,B**; I	3	0.0053
**130771**	Q8I9P8_9BIVA (aa7-550)	2.2e-153	51.6%	α-amylase; domain: α-amylase, signal peptide;	**-H**	**Y,O**; I	4	0.0022
**363559**	(aa432-698)	1.3e-55	52.9%	Lotgi1|82332 (1.2e-71)		**Y,O**; I	2	0.0013
					**+H**	**Y,O**; S<I	10	0.0117
						**Y,O**; S<I	4	0.0027
**131464**				Uncharacterized; signal peptide	**-H**	**Y,B**; I	2	0.0011
					**+H**	**Y,O,B**; I	3	0.0021
**132**				Uncharacterized; 12% Q	**-H**	**Y,O,B**; S<I	22	0.3538
					**+H**	**Y,O,B**; S<I	21	0.2887
**1323**	A7T0W4_NEMVE (aa119-414)	1.3e-26	40.5%	Uncharacterized; domain: polysaccharide deacetylase/chitinase; Lotgi1|153749 (3.5e-39)	**-H**	**Y,O,B**; S<I	4	0.1272
					**+H**	**Y,O,B**; S<I	7	0.3197
**13233**	L1JQJ7_GUITH	1.1e-31	57.3%	Uncharacterized; domain: thioredoxin_like_fold	**-H**	**Y,B**; S<I	1	0.0031
					**+H**	**Y,B**; S,I	1	0.0011
**13347**	R7UUB1_9ANNE (aa45-687)	5.5e-190	83.5%	Uncharacterized; domains: DnaK/HSP70; Lotgi1|200867 (0e0)	**-H**	-	-	-
					**+H**	**Y,O,B**; I	2	0.0003
**133605**	R7UA33_9ANNE (aa16-268)	2.4e-21	30.3%	Uncharacterized; domains: transmembrane, peptidase_M13; Lotgi1|193572 (9.5e-26/3.1e-124)	**-H**	-	-	-
**46072**	(aa352-766)	2.2e-76	40.6%					
					**+H**	**Y**; I	1	0.0008
						**Y**; I	3	0.0018
**134534**	K1P339_CRAGI (aa1-665)	1.1e-173	73.1%	Vinculin; Lotgi1|102378 (0e0)	**-H**	-	-	-
					**+H**	**Y,O**; I	6	0.0003

**13504**	K1RXH3_CRAGI	4.7e-20	41.2%	Similar to Universal stress protein A-like protein; domain: UspA; Lotgi1|149221 (1.5e-31)	**-H**	**Y,O,B**; I	5	0.0150
					**+H**	**Y,O,B**; S<I	5	0.0399
**13525**	C1K7M3_ICTPU	5.8e-15	51.3%	Annexin (A4); domains: annexin; Lotgi1|219490 (7.9e-112)	**-H**	**Y,O,B**; I	6	0.0047
					**+H**	**Y,O,B**; S<I	8	0.0112
**1365**	C3Y8Z2-BRAFL (aa20-292)	5.9e-48	51.8%	Similar to periostin; domains: FAS1, signal peptide; Lotgi1|200505 (2.8e-68)	**-H**	**Y,O**; S<I	5	0.0043
					**+H**	**Y,O,B**; S<I	10	0.0251
	K1S4H5_CRAGI			Adenosylhomocysteinase; Lotgi1|105848 (9.9e-89)				
**136589**	(aa35-307)	1.4e-106	89.7%		**-H**	**Y,O,B**; I	3	0.0044
**29551**	(aa265-404)	1.5e-56	92.8%			**Y,O,B**; I	2	0.0010
					**+H**	**Y,O**; S<I	3	0.0091
						**Y,O**; I	3	0.0063
**13700**	E7D158_LATHE	2.0e-5	28.1%	Profilin fragment; domain: profilin	**-H**	**Y,B**; I	2	0.0014
					**+H**	**Y,O**; I	2	0.0021
**13725**	K1QEA6_CRAGI	0e0	74.6%	Phosphoenolpyruvate carboxykinase [GTP]; Lotgi1|203707 (0e0)	**-H**	**Y,O**; I	3	0.0008
					**+H**	**Y,O**; S<I	13	0.0049
**137918**				Uncharacterized; domain: chromophore_lyase_CpcT/CPeT	**-H**	**Y,O,B**; S<I	4	0.0391
					**+H**	**Y,O,B**; S<I	4	0.0535
**138139**				Uncharacterized; transmembrane; 15% Q, 10% L, 10% S	**-H**	**Y,O,B**; S<I	5	0.0078
					**+H**	**Y,O**; S<I	5	0.0065
**1387**	Q86D82_APLCA (aa1-246)	6.7e-109	98.8%	β-tubulin; Lotgi1|223043 (8.4e-153)	**-H**	**Y,O,B**; S<I	4	0.0419
					**+H**	**Y,O,B**; S<I	5	0.0442
**139966**	K1PPK3_CRAGI (aa279-484)	2.8e-17	33.0%	Similar to fibropellin-1; domains: signal peptide, CBM_14/CHIT_BIND _II; Lotgi1|152043 (3.2e-20)	**-H**	**Y,O**; I	1	0.0020
					**+H**	**Y,O**; S,I	4	0.0077
**13999**	R7U801_9ANNE (aa569-977)	2.4e-135	74.6%	Uncharacterized/filamin; domains: filamin/ABP280_repeat; shares 2 peptides with contig_1628; Lotgi1|178063 (0e0)	**-H**	**-**	-	-
					**+H**	**Y,O**; I	7	0.0026

<table>
<tr><td>14003</td><td>K1PRD3_CRAGI
(aa985-1278)</td><td>3.6e-22</td><td>30.1%</td><td>Similar to IgGFc-binding protein; domains: CBM_14/CHIT_BIND_II; Shares peptide with 101824; Lotgi1|231869 (2.6e-51)</td><td>-H</td><td>Y,O,B; S<I</td><td>14</td><td>0.0788</td></tr>
<tr><td></td><td></td><td></td><td></td><td></td><td>+H</td><td>Y,O,B; S,I</td><td>18</td><td>0.3782</td></tr>
<tr><td>140752</td><td>J9JZU3_ACPI</td><td>7.5e-69</td><td>62.5%</td><td>Uncharacterized; domains: EFh;
Lotgi1|134987 (1.7e-144)</td><td>-H</td><td>-</td><td>-</td><td>-</td></tr>
<tr><td></td><td></td><td></td><td></td><td></td><td>+H</td><td>Y,O; I</td><td>3</td><td>0.0009</td></tr>
<tr><td>140914
54526</td><td>Q25017_HELAS
(aa1-497)
(aa506-646)</td><td>5.1e-115
1.5e-58</td><td>97.8%
99.3%</td><td>Neurofilament protein NF70; shares many peptides with 87417
Lotgi1|125719 (0e0)/Lotgi1|204366 (3.2e-4372.5e-50)</td><td>-H</td><td>Y,O;B; S<I
Y,O; S<I</td><td>7
4</td><td>0.0213
0.0400</td></tr>
<tr><td></td><td></td><td></td><td></td><td></td><td>+H</td><td>Y,O,B; I
Y,O; I</td><td>4
1</td><td>0.0020
0.0025</td></tr>
<tr><td>17464
87417</td><td>Q25017_HELAS
(aa496-646)
(aa1-497)</td><td>1.9e-45
1.2e-94</td><td>71.5%
81.5%</td><td>Similar to NF70; Lotgi1|165340 (0e0)</td><td>-H</td><td>Y,O,B; S<I
Y,O,B; S<I</td><td>8
21</td><td>0.0918
0.1645</td></tr>
<tr><td></td><td></td><td></td><td></td><td></td><td>+H</td><td>Y,O,B; I
Y,O,B; I</td><td>3
9</td><td>0.0037
0.0095</td></tr>
<tr><td>141518</td><td>K1QRU9_CRAGI</td><td>2.4e-73</td><td>78.4%</td><td>Epidermal growth factor receptor kinase substrate 8-like protein 1; domains: SH3, tensin_phosphotyrosin-binding;
Lotgi1|115450 (7.4e-147)</td><td>-H</td><td>Y,O,B; I</td><td>2</td><td>0.0005</td></tr>
<tr><td></td><td></td><td></td><td></td><td></td><td>+H</td><td>Y,O,B; S<I</td><td>6</td><td>0.0011</td></tr>
<tr><td>14188</td><td>K1PF62_CRAGI
(aa87-287)</td><td>4.6e-36</td><td>55.2%</td><td>Uncharacterized; domain: aldo/keto_reductase;
Lotgi1|137310 (1.7e-63)</td><td>-H</td><td>-</td><td>-</td><td>-</td></tr>
<tr><td></td><td></td><td></td><td></td><td></td><td>+H</td><td>Y; I</td><td>3</td><td>0.0005</td></tr>
<tr><td>143358</td><td>R7VES2_9ANNE
(aa94-477)</td><td>8.6e-54</td><td>38.5%</td><td>Uncharacterized; domains: DUF23/glycol_transf_92, transmembrane;
Lotgi1|155701 (5.3e-79)</td><td>-H</td><td>-</td><td>-</td><td>-</td></tr>
<tr><td></td><td></td><td></td><td></td><td></td><td>+H</td><td>Y,O; I</td><td>2</td><td>0.0005</td></tr>
<tr><td>142698</td><td>K1R3E8_CRAGI
(aa790-1236)</td><td>7.9e-20</td><td>26.8%</td><td>Similar to collagen α5(VI); domains: VWA, signal peptide; 11% T; Lotgi1|232362 (2.1e-25)</td><td>-H</td><td>Y,O,B; I</td><td>5</td><td>0.0042</td></tr>
<tr><td></td><td></td><td></td><td></td><td></td><td>+H</td><td>-</td><td>-</td><td>-</td></tr>
<tr><td>14344</td><td></td><td></td><td></td><td>Uncharacterized; 17% Q, 10% L, 17% P</td><td>-H</td><td>Y,O,B; S,I</td><td>9</td><td>1.6592</td></tr>
<tr><td></td><td></td><td></td><td></td><td></td><td>+H</td><td>Y,O,B; S<I</td><td>11</td><td>2.9614</td></tr>
<tr><td>143763</td><td>K1S2V5_CRAGI
(aa8-474)</td><td>4.8e-120</td><td>58.0%</td><td>N-acetylglucosamine-6-sulfatase;
Lotgi1|152768 (0e0)</td><td>-H</td><td>-</td><td>-</td><td>-</td></tr>
<tr><td></td><td></td><td></td><td></td><td></td><td>+H</td><td>Y,O; S<I</td><td>3</td><td>0.0020</td></tr>
</table>

**144901**	K1PP66_CRAGI (aa159-317)	6.6e-5	24.8%	Uncharacterized	**-H**	**Y,O,B**; I	2	0.0020
					**+H**	**Y,O**; S,I	3	0.0039
**145325**	ENO_DORPE	1.3e-139	78.7%	Enolase; Lotgi1|177346 (0e0)	**-H**	**Y,O,B**; I	4	0.0013
					**+H**	**Y,O,B**; S<I	7	0.0043
**14554**	D6X2U0_TRICA (aa610-771)	1.5e-40	54.9%	Uncharacterized; domain: peptidase_M13; Lotgi1|165736 (2.4e-56)	**-H**	**Y,B**; I	3	0.0084
					**+H**	-	-	-
**14570**<br>**36869**<br>**6241**	G3FPE6_HELLU<br>(aa775-997)<br>(aa2774-2945)<br>(aa1534-1867)	3.0e-75<br>5.8e-95<br>4.9e-146	91.9%<br>94.2%<br>94.9%	Hemocyanin αD; shares 1 peptide with 1031 (β-chain)	**-H**	**B**; I<br>**Y,B**; I<br>**Y,B**; I	1<br>1<br>1	0.0002<br>0.0025<br>0.0015
					**+H**	-<br>**Y,O,B**; I<br>-	-<br>1<br>-	-<br>>0.0001<br>-
**14584**	M7AZ14_CHEMY	3.4e-6	33.3%	Similar to WAP four-disulfide core domain protein 3; domains: EGF, WAP, signal peptide; 12% C, 10% P, 10% T	**-H**	**Y,O,B**; S<I	3	0.0800
					**+H**	**Y,O,B**; S>I	3	0.0650
**1459**	R4WPX3_9HEMI	8.0e-53	71.7%	Ribosomal protein L9; Lotgi1|217766 (8.7e-74)	**-H**	**-**	-	-
					**+H**	**Y,B**; I	2	0.0016
**146226**	R7VJR9_9ANNE (aa40-310)	2.3e-30	39.2%	Uncharacterized; domains: DUF4139, DUF4140; 11% E, 10%K; Lotgi1|230318 (7.4e-65)	**-H**	**Y,O,B**; I	3	0.0005
					**+H**	**Y,O,B**; I	7	0.0039
**14755**	C3ZH75_BRAFL (aa166-401)	1.8e-24	34.3%	Uncharacterized/similar to protein yellow; domain: major_royal_jelly_related/TolB_like	**-H**	**O,B**; I	3	0.0027
					**+H**	-	-	-
**14802**	L7M880_9ACAR	9.4e-142	72.9%	Putative rab geranylgeranyltransferase component a rab escort protein; domains: RAB_GDI; Lotgi1|179044 (0e0)	**-H**	**-**	-	-
					**+H**	**Y,O**; I	4	0.0005
**14834**	K1RE19_CRAGI (aa56-424)	1.5e-35	33.2%	Similar to V-type proton ATPase subunit S1; domains: transmembrane, ATP_synth_S1; Lotgi1|175264 (1.8e-51)	**-H**	**-**	-	-
					**+H**	**Y,O**; S<I	4	0.0024

**149478**<br>**152245**	L7MKZ3_9ACAR<br>(aa353-529)<br>(aa66-359)	3.8e-39<br>7.6e-53	57.8%<br>44.5%	Putative phospholipase d3 protein; domain: PLDc, transmembrane; Lotgi1|119020 (3.2e-56)	**-H**	**Y,O**; I	1	0.0003
					**+H**	**Y,O**; S<I<br>**Y,O**; I	3<br>1	0.0048<br>0.0024
**14978**	G1KP65_ANOCA<br>(aa455-596)	3.0e-34	67.1%	Uncharacterized/Vacuolar proton ATPase; domains: transmembrane, V-ATPase_I; Lotgi1|108161 (1.7e-62)	**-H**	**-**	-	-
					**+H**	**Y,O**; I	2	0.0261
**14997**	K1S6V7_CRAGI<br>(aa1-587)	1.7e-209	83.3%	Serine/threonine-protein phosphatase 2A 65 kDa regulatory subunit A alpha isoform; domains: HEAT; Lotgi1|165581 (0e0)	**-H**	**-**	-	-
					**+H**	**Y,B**; I	4	0.0004
**150060**	R7TKT4_9ANNE	2.4e-155	75.7%	Uncharacterized/ribosomal protein S6 kinase; Lotgi1|236015 (0e0)	**-H**	**-**	-	-
					**+H**	**Y**; I	3	0.0004
**150198**	A7RQD5_NEMVE<br>(aa251-495)	1.9e-22	30.7%	Uncharacterized; shares peptides with 7323	**-H**	**Y,O,B**; S<I	7	0.0260
					**+H**	**Y,O**; S<I	8	0.0007
**150378**	H2M677_ORYLA	3.3e-40	33.4%	Uncharacterized; domains: Armadillo_like, ataxin-10; Lotgi1|167953 (1.1e-53)	**-H**	**-**	-	-
					**+H**	**Y**; S<I	5	0.0011
**1504**				Uncharacterized, fragment; signal peptide; without signal peptide 16% G, 12% L, 21% M, 10% S in 58aa; only 1 peptide possible!	**-H**	**Y,O,B**; S>I	1	1.2760
					**+H**	**Y,O,B**; S>I	1	1.1542
**15054**	R7UXV8_9ANNE	0e0	72.9%	Uncharacterized/ATP-citrate synthase	**-H**	**-**	-	-
					**+H**	**Y,O**; I	3	0.0001
**15065**	F6U982_XENTR<br>(aa7-360)	1.7e-95	66.9%	Similar to Peptidase (mitochondrial processing) beta; domain: peptidase_M16; Lotgi1|105568 (9.7e-124)	**-H**	**Y,O,B**; I	4	0.0021
					**+H**	**Y,O**; I	6	0.0044
**15106**	R7UIL6_9ANNE	2.5e-91	66.7%	Uncharacterized/similar to sorbitol dehydrogenase; Lotgi1|208637 (2.1e-172)	**-H**	**-**	-	-
					**+H**	**Y**; I	2	0.0002
**1524**	F8V2T7_BATAR<br>(aa2-313)	5.2e-105	82.4%	Putative Na^+/K^+-ATPase alpha subunit; domains: transmembrane, P_type_ATPase; Lotgi1|169373 (1.3e-155)	**-H**	**-**	-	-
					**+H**	**Y**; I	2	0.0009

**15247**	R7VC37_9ANNE	0e0	65.4%	6-phosphofructokinase; Lotgi1|235800 (0e0)	**-H**	-	-	-
					**+H**	**Y,O**; I	10	0.0030
**15338**	K1P6B3_CRAGI	3.9e-92	60.6%	Endoplasmic reticulum resident protein ERp44; domains: thioredoxin, signal peptide; Lotgi1|131935 (3.0e-161)	**-H**	**Y**; I	3	0.0007
					**+H**	**Y,O**; S<I	6	0.0046
**15360**	N6UCC9_9CUCU (aa4-266)	1.3e-22	30.3%	Uncharacterized/serine protease inhibitor; domains: serpin, signal peptide; Lotgi1|129913 (2.2e-29)	**-H**	**Y,O,B**; I	2	0.0058
					**+H**	**Y,O**; I	4	0.0118
	G3FPE7_HELLU			Hemocyanin αN;				
**154**	(aa2266-2871)	0e0	90.9%	Lotgi1|234481 (2.8e-9)/163934 (4.0e-8)	**-H**	**Y,O,B**; I	12	0.0103
**157**	(aa2056-2226)	4.3e-74	94.2%			**Y,O,B**; I	3	0.0111
**3460**	(aa791-1054)	2.4e-119	93.6%			**Y,O,B**; I	8	0.0224
**3785**	(aa1669-1887)	8.2e-107	99.1%			**Y,O,B**; I	4	0.0080
**571**	(aa1129-1606)	0e0	94.4%			**Y,B**; I	5	
					**+H**	**Y,O,B**; I	14	0.0091
						**Y,O,B**; S<I	3	0.0071
						**Y,O,B**; I	6	0.0192
						**Y,O,B**; I	2	0.0060
						**Y,O,B**; I	4	0.0027
**15424**	K1RH58_CRAGI	5.8e-216	80.7%	Sarcomeric α-actinin; domains: CH, EFh, actinin Lotgi1|189716 (0e0)	**-H**	**Y,O,B**; S<I	13	0.0025
					**+H**	**Y,O,B**; S<I	26	0.0189
**15432**	K1QWZ3_CRAGI (aa37-252)	1.8e-46	51.6%	Uncharacterized; 11% G, 13% S; ; shares peptides with 15433 Lotgi1|234011 (1.9e-41)	**-H**	**Y,O,B**; I	4	0.0087
					**+H**	**Y,O,B**; S<I	5	0.0141
**15433**	K1QWZ3_CRAGI (aa35-252)	8.0e-49	53.9%	Uncharacterized; domains: SH3_like, bacterial type; 10% A, 11% G, 12% S; shares peptides with 15432; Lotgi1|163736 (1.8e-40)	**-H**	**Y,O,B**; I	6	0.0366
					**+H**	**Y,O,B**; S<I	3	0.0061
**15472**	K1QFS4_CRAGI	1.9e-145	85.6%	Actin-related protein 2; Lotgi|178898 (0e0)	**-H**	-	-	-
					**+H**	**Y,B**; I	2	0.0008
**15506**				Uncharacterized; signal peptide; 16% L	**-H**	**Y,O,B**; S>I	1	0.0686
					**+H**	**Y,O,B**; S,I	1	0.0659

**157680**				Uncharacterized, similarity to very Q-rich proteins; 13% R, 30% Q mainly concentrated in C-term	**-H**	**Y,O,B**; I	1	0.0003
					**+H**	**Y,O,B**; I	2	0.0004
**158**	Q9NJ19_ARGIR	5.7e-126	67.7%	Catchin; domain: myosin_tail_1; shares peptides with 1617; Lotgi1|170428 (0e0)	**-H**	**Y,O**; I	17	0.0019
					**+H**	-	-	-
**159000**	K1P8K1_CRAGI	4.5e-35	32.5%	Meteorin-like protein; see also 126964; Lotgi1156516 (6.8e-48)	**-H**	**Y,O,B**; I	2	0.0025
					**+H**	**Y,O**; S<I	10	0.0153
**1594**	Q1EL60_9EUPU	1.6e-6	32.3%	aa173-258 similar to CHAII-like protein; domain: H-lectin; shares peptides with 1595; also similar to Q5F4K1_9EUPU (CHAII protein)	**-H**	**Y,O,B**; I	2	0.0050
					**+H**	**O,B**; I	3	0.0040
**1595**	Q1EL60_9EUPU	1.2e-4	29.7%	aa173-258 similar to CHAII-like protein; domain: H-lectin; shares peptides with 1594; also similar to Q5F4K1_9EUPU (CHAII protein)	**-H**	**Y,O,B**; I	4	0.0051
					**+H**	**Y,O,B**; S<I	5	0.0218
**15953**				Uncharacterized; 11% G, 11% L	**-H**	**Y,O,B**; S<I	3	0.0196
					**+H**	**Y,O,B**; S<I	5	0.0489
**15995**	C3ZEP8_BRAFL (aa1-252)	5.5e-43	44./%	Uncharacterized/epithelial chloride channel protein; domains: CLCA_N, signal peptide: Lotgi1|238844 (8.7e-110)	**-H**	**Y,O**; I	3	0.0006
					**+H**	**Y,O**; S<I	6	0.0070
**1604**				Uncharacterized; 31% Q, 23% P	**-H**	**Y,O,B**; S>I	2	0.2137
					**+H**	**Y,O,B**; S>I	2	0.4750
**1611**				Uncharacterized; signal peptide	**-H**	**Y,O,B**; S<I	4	0.0795
					**+H**	**O,B**; I	3	0.0042
**1617**	G4V4Y8_DORPE (aa250-1938)	0e0	77.0%	Myosin heavy chain isoform C; shares peptides with 158; Lotgi1198366 (0e0)	**-H**	**Y**; I	2	0.0002
					**+H**	-	-	-
**161838**	R7TWN6_9ANNE	2.7e-33	37.5%	Uncharacterized; 11% L, 12% S; Lotgi1|152551 (7.0e-64)	**-H**	**Y,O,B**; S	2	0.0017
					**+H**	-	-	-
**1628**	G7Y5W0_CLOSI (aa1664-2333)	9.0e-192	65.1%	Filamin; Lotgi|158607 (0e0)	**-H**	**Y,O,B**; I	3	0.0005
					**+H**	**Y,B**; I	2	0.0008

**1647**				Uncharacterized; 12% P, 11% S, 11% T; domain:Sushi/SCR/CCP Lotgi1|159256 (5.2e-10)	**-H**	**Y,O,B**; S>I	9	0.3743
					**+H**	**Y,O,B**; S>I	9	0.3790
**165344**				Uncharacterized; 11% T, 11% V; Lotgi169600 (4.6e-7)	**-H**	**Y,O,B**; I	4	0.0109
					**+H**	**Y,O,B**; S<I	7	0.0467
**16679**	K3WH68_PYTUL	0e0	62.3%	Urease; Lotgi|105464 (0e0)	**-H**	-	-	-
					**+H**	**Y**; I	5	0.0004
**16710**	K1QZ49_CRAGI (aa59-407)	2.3e-45	35.0%	Similar to adipocyte plasma membrane-associated protein; domains: signal peptide, TolB_like/ strictosidine synthase; Lotgi1|115637 (3.0e-47); see also contig_221/contig_58150/contig_8812	**-H**	**Y,O**; I	4	0.0014
					**+H**	**Y,O**; S<I	9	0.0181
**16729**	Q9BLF6_OCTVU	1.7e-80	56.0%	D-lactate dehydrogenase; Lotgi1|125448 (1.3e-134)	**-H**	-	-	-
					**+H**	**Y,O**; I	3	0.0011
**168604**	A7S7T9_NEMVE (aa1-241)	2.4e-53	56.0%	Uncharacterized; domains:transmembrane, HSP70; Lotgi1|159745 (1.5e-103)	**-H**	-	-	-
					**+H**	**Y,O**; S<I	4	0.0106
**16878**				Uncharacterized; 29% Q, 16% P;	**-H**	**Y,O,B**; S>I	3	0.9451
					**+H**	**Y,O,B**; S>I	3	1.0824
**1696**	Q4H45_CRAGI	2.1e-55	85.8%	Ribosomal protein S18	**-H**	**Y,O,B**; I	4	0.0052
					**+H**	**Y,B**; I	2	0.0044
	K1PTY5_CRAGI			Similar to Protocadherin Fat 4; domains:cadherin Lotgi1|154324 (0e0)/229125 (2.0e-163); 2 proteins?				
**1697**	(aa2393-4946)	3.1e-49	30.4%		**-H**	**Y,O,B**; I	18	0.0061
**22033**	(aa7445-8584)	2.0e-144	37.3%			**Y,O,B**; I	11	0.0045
**246989**	(aa1090-1277)	3.3e-21	37.8%			**Y,O**; I	3	0.0058
**291505**	(aa2381-2937)	1.3e-59	35.1%			**Y,O,B**; I	4	0.0110
**3849**	(aa9466-10119)	4.5e-41	34.8%			**Y,O,B**; I	4	0.0030
					**+H**	**Y,O,B**; S<I	25	0.0127
						**Y,O,B**; S<I	19	0.0082
						**Y,O**; I	3	0.0143
						**Y,O,B**; S<I	6	0.0250
						**Y,O,B**; S<I	6	0.0080

**169764**	R7VB66_9ANNE (aa53-195)	3.0e-31	54.5%	Uncharacterized; signal peptide	**-H**	**Y,O**; S<I	5	0.0302
					**+H**	**Y,O**; S<I	6	0.1591
**170169**	F6MFF0_BRABE (aa29-608)	2.9e-31	26.2%	Similar to α2-macroglobulin; domains: A2M_N, A2M_N_2; Lotgi1|229818 (4.4e-26)	**-H**	-	-	-
					**+H**	**Y,O**; I	3	0.0005
**1703**				Uncharacterized; 10% R, 11% Q, 10% G, 14% T	**-H**	**Y,O**; I	2	0.0004
					**+H**	**Y,O**; S<I	7	0.0020
**172966**	B4L6S0_DROMO (aa149-455)	7.4e-89	61.6%	Uncharacterized; domain: phosphoesterase; Lotgi1|150028 (3.2e-112)	**-H**	**Y,O**; I	2	0.0006
					**+H**	**Y,O**; S<I	4	0.0073
**1744**	Q6H3X5_PINFU	1.1e-84	87.6%	QM protein (ribosomal protein); Lotgi1|229535 (3.4e-127)	**-H**	-	-	-
					**+H**	**Y,B**; I	2	0.0010
	ESRSV6_HALDI			Paramyosin; Lotgi1|205639 (1.1e-103)/104779 (0e0)				
**1757**<br>**2598**	(aa589-860)<br>(aa34-562)	4.2e-45<br>1.1e-73	75.1%<br>72.6%		**-H**	**Y,O**; S<I<br>**Y,O**; S<I	14<br>22	0.0323<br>0.0167
					**+H**	**O**, I<br>**O,B**; I	1<br>3	0.0003<br>0.0004
**1758**	K1R0Y9_CRAGI	1.1e-109	82.0%	ADP,ATP carrier protein; Lotgi1|232343 (1.0e-161)	**-H**	**Y,O,B**; S<I	4	0.0065
					**+H**	**Y,O,B**; S<I	4	0.0117
**1764**	TPM_HELAS	1.6e-22	52.3%	Tropomyosin; shares peptide with 6620; Lotgi1|162586 (2.0e-54)	**-H**	**Y,O,B**; I	3	0.0033
					**+H**	**Y,O,B**; I	7	0.0105
**1768**	E9H8M4_DAPPU	4.5e-84	81.1%	Uncharacterized; shares peptides with 19017, 27726,4405, 7641, 8216; domain: 14-3-3; Lotgi1|107549 (1.9e-116)	**-H**	**Y,O,B**; I	4	0.0103
					**+H**	**Y,O,B**; S<I	6	0.0291
**17713**				Uncharacterized; signal peptide	**-H**	**Y,O,B**; I	5	0.0060
					**+H**	**B**; I	2	0.0011
**18035**	R7U8H5_9ANNE	1.8e-38	40.9%	Uncharacterized/similar to D-beta-hydroxybutyrate dehydrogenase (K1Q2H3_CRAGI); Lotgi1|168913 (2.2e-78)	**-H**	**Y,O**; I	2	0.0010
					**+H**	-	-	-

**180559**	F7HD86_CALJA (aa80-245)	5.2e-12	34.5%	Uncharacterized; domain: peptidase_S1; Lotgi1|225501 (1.3e-8)	**-H**	**Y,O,B**; I	5	0.0262
					**+H**	**Y,O**; S<I	6	0.0553
**18225**	I3J590_ORENI (aa3-304)	4.1e-85	62.9%	Uncharacterized/actin-related protein 2/3 complex subunit 1A; Lotgi1|167800 (2.8e-155)	**-H**	**-**	-	-
					**+H**	**Y**; I	2	0.0009
**18268**	A7RQD5_NEMVE	8.0e-38	28.4%	Uncharacterized; signal peptide; Lotgi1|160009 (9.7e-7)	**-H**	**Y,O,B**; I	10	0.0117
					**+H**	**Y,O,B**; S<I	12	0.0301
**18276**	B8Y4I8_BIOGL	1.9e-188	91.6%	HSP60; Lotgi1|237408 (0e0)	**-H**	**Y,O,B**; I	3	0.0007
					**+H**	**Y,O,B**; S<I	8	0.0014
**1837**				Uncharacterized; 12% I, 15% S in 73aa	**-H**	**Y,O,B**; S<I	2	0.3739
					**+H**	**Y,O,B**;I	2	0.0434
**1850**				Uncharacterized; 22% P; transmembrane (or signal peptide if correct sequence starts at the first Met)	**-H**	**Y,O,B**; S>I	15	1.5474
					**+H**	**Y,O,B**; S>I	15	1.3134
**1869**	I1SKJ0_APLCA	2.5e-50	100%	Histone H2A; Lotgi1|156628 (1.3e-61)	**-H**	**Y,O,B**; S,I	4	0.1482
					**+H**	**Y,O,B**; S,I	2	0.0923
**188205**	H2T7Q9_TAKRU (aa1037-1211)	7.1e-6	31.7%	Uncharacterized; domain: cadherin Lotgi1|154324 (5.6e-10)	**-H**	**O,B**; I	1	0.0034
					**+H**	**Y,O**; I	1	0.0108
**18938**	E2C4M7_HARSA	4.0e-46	39.0%	Lamin Dm0; domain: Lamin_tail/IF; Lotgi1|179001 (5.3e-104)	**-H**	**Y,O,B**; I	4	0.0009
					**+H**	**Y,O,B**; I	4	0.0011
**19017**	14331_CAEEL	2.2e-54	62.1%	aa49-287 similar to 14-3-3-like protein 1; shares peptide with 19017, 27726,4405, 7641, 8216; Lotgi1|222442 (7.4e-86)	**-H**	**Y,O,B**; S<I	3	0.0038
					**+H**	**Y,O,B**; S<I	3	0.0069
**190546**	A7RPT6_NEMVE (aa41-341)	4.8e-33	37.1%	Uncharacterized/similar to 1-phosphatidylinositol 4,5-bisphosphate phosphodiesterase; domains: Phospholipase_C_X, PH, EFh; Lotgi1|62286 (6.2e-65)	**-H**	**-**	-	-
					**+H**	**Y**; I	2	0.0005
**191**	K1QW36_CRAGI	1.9e-65	71.2%	60S ribosomal protein L6; Lotgi1|207552 (4.9e-84)	**-H**	**Y,O,B**; I	1	0.0005
					**+H**	**Y,O,B**; I	1	0.0004

**193976**<br>**36933**	K1QSS1_CRAGI<br>(aa1-173)<br>(aa175-356)	4.3e-59<br>1.1e-35	74.6%<br>56.6%	Uncharacterized; domain: Ndr; Lotgi1|150144 (1.5e-100/4.2e-61)	**-H**	**Y,O,B**; I<br>**Y,O,B**; I	3<br>3	0.0049<br>0.0065
					**+H**	**Y,O**; S<I<br>**Y,O,B**; I	4<br>7	0.0194<br>0.0217
**194050**	A4L7H3_BIOGL	9.8e-100	53.8%	Gram-negative bacteria binding protein; domains: Glyco_hydro_16/conA_like_lectin, transmembrane; Lotgi1|233531 (3.4e-102)	**-H**	**Y**; I	2	0.0012
					**+H**	-	-	-
**195796**	K1QBI9_CRAGI<br>(aa169-536)	4.1e-86	54.1%	Cytosolic purine 5'-nucleotidase; domain: HAD_like; Lotgi1|190218 (4.0e-129)	**-H**	**-**	-	-
					**+H**	**Y,O**; I	3	0.0007
**19597**	R7TY81_9ANNE	1.2e-149	57.8%	Uncharacterized/similar to plastin; domains:actinin/CH, EFh; Lotgi1|152582 (0e0)	**-H**	**Y,O**; I	3	0.0003
					**+H**	**Y,O**; I	11	0.0041
**196388**				Uncharacterized; 12% A, 11% G, 11% P, 14% S; only 1 peptide possible!	**-H**	**Y,O,B**; S,I	1	0.1994
					**+H**	**Y,O,B**; S<I	1	0.1422
**196754**				Uncharacterized; 14% P, 12% S; P-rich C-term	**-H**	**-**	-	-
					**+H**	**Y,O**; S	3	0.0001
**19784**	K1PWM6_CRAGI	1.0e-35	43.8%	Neural/ectodermal development factor IMP-L2; domains: signal peptide, IG; Lotgi1|155640 (1.9e-64)	**-H**	**Y,O**; I	2	0.0025
					**+H**	**Y,O,B**; S<I	2	0.0052
**198**	A6YM37_9EUPU<br>(aa143-314)	1.2e-29	45.9%	Similar to matrilin_like 40kDa; domains: EGF_like, VWA; Lotgi1|74980 (2.6e-14)	**-H**	**Y,O,B**; I	2	0.0051
					**+H**	**Y,O,B**; I	4	0.0072
**1986**	RS3A_APLCA	9.6e-102	92.9%	40S ribosomal protein S3a; Lotgi1|202499 (2.7e-98)	**-H**	**Y,O,B**; I	1	0.0013
					**+H**	**Y,O,B**; I	3	0.0020
**19980**	Q56UG2_LYMST	6.4e-34	37.3 %	aa1-215 similar to Lipopolysaccharide binding protein-like protein/Bactericidal permeability increasing protein; Lotgi1|231708 (1.7e-48)	**-H**	**Y,O,B**; S<I	8	0.0223
					**+H**	**Y,O,B**; S<I	10	0.0722

**20097**	Q596I0_CRAGI	0e0	75.5%	Phosphorylase; Lotgi1|201834 (0e0)	**-H**	-	-	-
					**+H**	**Y,O**; I	4	0.0005
**201**				Uncharacterized; signal peptide; 11% N, 10% Q	**-H**	**Y,O,B**; S<I	10	0.5010
					**+H**	**Y,O,B**; S<I	11	1.2000
**20308**	Q2LZN0_DROPS (aa81-527)	1.6e-9	27.0%	aa49-467 similar to Dpse/GA10422/alkaline phosphatase; domains: signal peptide, alk_phos; Lotgi1|236474 (1.3e-13)	**-H**	**Y,O,B**; S<I	12	0.0475
					**+H**	**Y,O,B**; S<I	14	0.1142
**20352**	K1QVK3_CRAGI	2.4e-192	45.5%	Glutamyl aminopeptidase; domains: transmembrane, peptidase_M1, ERAP1_C; Lotgi1|130453 (0e0)	**-H**	-	-	-
					**+H**	**Y,O**; I	4	0.0005
**20360**	H9K6W1_APIME (aa687-1009)	3.9e-37	37.5%	Similar to cadherin; domains: cadherin; Lotgi1|167053 (1.5e-35); see also contig_75801!	**-H**	**Y,O,B**; S<I	7	0.0313
					**+H**	**Y,O,B**; S<I	8	0.1083
**20638**	C3Y2H6_BRAFL	1.2e-36	53.1%	Uncharacterized/similar to V-type proton ATPase subunit E (K1PIH2_CRAGI); Lotgi1|209321 (4.5e-60)	**-H**	**Y,O,B**; S<I	8	0.0155
					**+H**	**Y,O,B**; S<I	14	0.0987
**2065**				Uncharacterized; signal peptide	**-H**	**Y,O,B**; S>I	2	0.0171
					**+H**	**Y,O,B**; S	2	0.0107
**207220**				Uncharacterized; signal peptide; 13%S, 11% T; Lotgi1|169040 (2.6e-9)	**-H**	**Y,O,B**; S>I	2	0.0234
					**+H**	**Y,O,B**; S,I	2	0.0088
**208250**				Uncharacterized; 13% Q, 10% G	**-H**	**Y,O,B**; S>I	2	0.0074
					**+H**	**Y,O**; S	1	0.0036
**21046**	B7PIZ1_IXOSC	3.4e-142	71.9%	GDI-1 GDP dissociation inhibitor, putative (Fragment); shares peptide with 14802; domains: Rab_GDI; Lotgi1|218952 (0e0)	**-H**	**Y,O,B**; S<I	3	0.0007
					**+H**	**Y,O**; I	7	0.0031
**21066**	R7T7N1_9ANNE	5.1e-20	42.1%	Uncharacterized; domains: signal peptide, EFh; Lotgi1|234943 (8.5e-56)	**-H**	-	-	-
					**+H**	**O**; S	3	0.0014
**2108**				Uncharacterized; 10% A, 10% Q	**-H**	**Y,O,B**; S<I	8	0.5032
					**+H**	**Y,O,B**; S<I	8	1.3212

**21112**				Uncharacterized; 18% P, 10% N	**-H**	**Y,O,B**; S>I	8	3.5681
					**+H**	**Y,O,B**; S>I	8	3.4095
**211922**				Uncharacterized; transmembrane	**-H**	**Y,O,B**; S,I	1	0.0127
					**+H**	**Y,O,B**; S,I	1	0.0116
**2120**	A7RQU4_NEMVE	6.3e-48	65.9%	Uncharacterized/V-type proton ATPase 21 kDa proteolipid subunit; domains: transmembrane, V-ATPase_proteolipid_subunit; Lotgi1|223715 (5.8e-97)	**-H**	**-**	-	-
					**+H**	**Y,O**; I	3	0.0290
**213707**				Uncharacterized	**-H**	**Y,O,B**; I	2	0.0012
					**+H**	**Y,O,B**; S<I	4	0.0027
**21551**	C3YEW8_BRAFL	1.1e-31	40.4%	Uncharacterized/similar to cysteine proteinase 3 (K1QEW8_CRAGI); domains:signal peptide, peptidase_C1A; Lotgi1|172091 (2.2e-45)	**-H**	**Y**; S<I	2	0.0027
					**+H**	**Y,O,B**; S,I	6	0.0204
**216988**				Uncharacterized; domains: FN3	**-H**	**Y,O,B**; I	7	0.0054
					**+H**	**Y,O**; S<I	14	0.0735
**2171**	K1RB91_CRAGI	0e0	61.3%	Neutral α-glucosidase AB; domains: signal peptide, glycoside_hydrolase_31; Lotgi1|217135 (0e0)	**-H**	**-**	-	-
					**+H**	**Y,O**; I	8	0.0007
**2177**	H3C8Z4_TETNG	3.4e-17	31.1%	Uncharacterized; similarity to BMSP (G1UCX0_ MYTGA) and Deleted in malignant brain tumors 1 protein(K1QA70_CRAGI); domains: EGF, VWA; Lotgi1|233176 (2.8e-22)	**-H**	**Y,B**; I	5	0.0027
					**+H**	**Y,O,B**; I	8	0.0098
**22065**	D2CLZ7_9PERC	2.0e-6	31.2%	Muscle fatty acid binding protein; domain: calycin	**-H**	**Y,B**; I	2	0.0013
					**+H**	-	-	-
**221**	K1QZ49_CRAGI (aa62-398)	2.3e-16	34.3%	Adipocyte plasma membrane-associated protein; Shares peptide with 8812; domains: signal peptide, TolB_like/strictosidine_synthase_related; See also contig_16710 and 6697; Lotgi1|115667 (2.4e-46)	**-H**	**Y,O,B**; S<I	13	0.0042
					**+H**	**Y,O,B**; S<I	24	0.0091
**22210**	G8DF88_DANPL	1.5e-159	76.5%	Citrate synthase; Lotgi1|170380 (0e0)	**-H**	**-**	-	-
					**+H**	**Y**; I	2	0.0005

**22472**	B5X305_SALSA	6.8e-87	44.0%	aa138-595 similar to cathepsin F and aa275-595 similar to aa378-714 of cathepsin F (K1QYP7_ CRAGI); domains: signal peptide, peptidase inhibitor I25 (cystatin) and C1A (papain); Lotgi1|207656 (6.3e-127)	**-H**	**Y,O,B**; I	8	0.0023
					**+H**	**Y,O,B**; S<I	10	0.0138
**22541**	K1PQI4_CRAGI	1.5e-51	58.8%	Enolase-phosphatase E1; 14% E; Lotgi1|168703 (8.7e-104)	**-H**	**Y,O,B**; S<I	8	0.0104
					**+H**	**Y,O,B**; I	4	0.0026
**22679**	B8K1X2_FENCH	4.4e-38	73.5%	Triosephosphate isomerase	**-H**	**Y,O,B**; S,I	1	0.0045
					**+H**	**Y,O,B**; S,I	3	0.0161
**226862**	R7VE82_9ANNE (aa204-838)	3.8e-127	45.2%	Uncharaterized/chitobiase/β-hexosaminidase; domain: glycoside_hydrolase_20; Lotgi1|168843 (0e0)	**-H**	-	-	-
					**+H**	**Y,O**; I	3	0.0009
**227**	D3BGG3_POLPA (aa471-830)	8.1e-5	23.9%	Similar to Zipper-like domain-containing protein; Lotgi1|160957 (2.2e-13)	**-H**	**Y,O,B**; S<I	16	0.3286
					**+H**	**Y,O,B**; S<I	14	0.1975
**228379**	A9UTT4_MONBE	2.6e-169	92.1%	β-tubulin; Lotgi1|178823 (0e0)	**-H**	**Y,O,B**; S<I	3	0.0061
					**+H**	**Y,O,B**; S<I	4	0.0078
**23**	K1QFW9_CRAGI	0e0	81.5%	Uncharacterized/similar to elongation factor 2; Lotgi1|180085	**-H**	**Y,O,B**; I	7	0.0017
					**+H**	**Y,O**; I	12	0.0063
**23080**	Q6TU32_APLCA	4.7e-116	74.3%	PKA type II regulatory subunit; Lotgi1|51570 (9.8e-146)	**-H**	-	-	-
					**+H**	**Y**; I	2	0.0007
**2339**	K1QQK6_CRAGI (aa35-165)	6.3e-10	37.1%	Calcium-dependent protein kinase isoform 2; domains: EFh; 11% D, 10% E, 13% K; Lotgi1|84721 (1.8e-19)	**-H**	**Y,O,B**; I	6	0.0116
					**+H**	**Y,O,B**; S<I	6	0.0307
**2357**				Uncharacterized protein; domains: signal peptide, LDL_recept_a; 15% T; Lotgi1|159329 (5.4e-9)	**-H**	**Y,O,B**; S<I	13	0.0890
					**+H**	**Y,O,B**; S,I	12	0.1907
**23598**				Uncharacterized; 26% Q, 12% S, 11% T; Lotgi1|172698 (1.3e-6)	**-H**	**Y,O,B**; S>I	1	0.0054
					**+H**	**Y,O,B**; S	1	0.0029

**2380**	K1QL55_CRAGI (aa413-735)	1.0e-114	77.8%	Protein phosphatase 1B; domain: protein_ phosphatase_2C; Lotgi1|194163 (0e0)	**-H**	-	-	-
					**+H**	**Y**; I	2	0.0004
**23899**	R7TVD2_9ANNE	0e0	57.9%	Uncharacterized/IQGAP protein; domains: IQ, RasGAP, CH; Lotgi1|162747 (0e0)	**-H**	-	-	-
					**+H**	**Y**; I	16	0.0011
**2429**	L7M4W5_9ACAR	2.0e-59	71.7%	Ribosomal protein L13; Lotgi1|197609 (2.6e-68)	**-H**	**Y,O,B**; I	2	0.0047
					**+H**	**Y,O,B**; I	2	0.0029
**24305**	K1QNT7_CRAGI	1.6e-152	70.5%	Aldehyde dehydrogenase, mitochondrial; Lotgi1|177987 (0e0)	**-H**	**Y,O,B**; I	2	0.0005
					**+H**	**Y,O**; I	1	0.0003
**24452**	N6T3L3_9CUCU	5-1e-117	56.9%	Uncharacterized/purple acid phosphatase; domains: phosphoesterase, signal peptide; Lotgi1|1500028 (1.2e-137)	**-H**	**Y,O**; I	1	0.0004
					**+H**	**Y,O**; I	4	0.0021
**24529**				Uncharacterized; signal peptide; 11% Q; Lotgi1|159706 (1.0e-16)	**-H**	**Y,O,B**; S,I	11	0.0142
					**+H**	**Y,O,B**; S,I	11	0.0128
**2456**	B3TK66_HALDV	4.4e-68	81.6%	Ribosomal protein S8; Lotgi1|239290 (1.5e-78)	**-H**	**Y,O,B**; I	3	0.0059
					**+H**	**Y,B**; I	3	0.0060
**2468**	D7RP02_HALDV	1.1e-90	71.6%	Voltage-dependent anion channel 2-like protein; domains: porin; Lotgi1|168464 (9.4e-1409	**-H**	**Y,O,B**; I	6	0.0102
					**+H**	**Y,O,B**; I	6	0.0111
**247174**	GNAQ_LYMST	1.0e-140	93.2%	Guanine nucleotide-binding protein G(q) subunit alpha; Lotgi1|194019 (0e0)	**-H**	-	-	-
					**+H**	**Y,O**; I	4	0.0010
**248122**	C3Z1I6_BRAFL (aa338-498)	6.1e-15	38.8%	Uncharacterized/similar to chitinase; domains: glyco_hydro_18/chitinase; Lotgi1|195782 82.1e-31)	**-H**	**Y,O,B**; S<I	3	0.0481
					**+H**	**Y,O,B**; S<I	4	0.1318
**24851**	MTAP_DANRE	1.7e-79	64.7%	S-methyl-5'-thioadenosine phosphorylase; Lotgi1|216998 (6.3e-131)	**-H**	-	-	-
					**+H**	**Y**; I	2	0.0005
**2503**	H2SK47_TAKRU	4.1e-40	83.9%	Uncharacterized/similar to V-ATPase proteolipid subunit; domains: VACATPASE, transmembrane; Lotgi1|224185 (1.3e-42)	**-H**	**O**; I	1	0.0096
					**+H**	**Y,O**; S<I	1	0.0570

<table>
<tr><td>**250568**
42886</td><td>K1QZN3_CRAGI
(aa1-424)
(aa636-1023)</td><td>1.1e-108
1.6e-101</td><td>69.3%
59.2%</td><td>Myosin-Id; Lotgi1|143556 (0e0)</td><td>**-H**</td><td>-</td><td>-</td><td>-</td></tr>
<tr><td></td><td></td><td></td><td></td><td></td><td>**+H**</td><td>**Y**; I
Y; I</td><td>6
2</td><td>0.0019
0.0005</td></tr>
<tr><td>**2521**</td><td>A5Z1D6_9EUPU
(aa1-156)</td><td>5.8e-12</td><td>34.4%</td><td>Epiphragmin; domain: transmembrane</td><td>**-H**</td><td>**Y,O,B**; I</td><td>6</td><td>0.0738</td></tr>
<tr><td></td><td></td><td></td><td></td><td></td><td>**+H**</td><td>**Y,O,B**; S<I</td><td>6</td><td>0.0585</td></tr>
<tr><td>**25443**</td><td>K1QAP9_CRAGI
(aa94-284)</td><td>2.8e-19</td><td>38.0%</td><td>Similar to collagen α1(XII) chain non-triple helical domain; domains: VWA; Lotgi1|123318 (2.7e-23)</td><td>**-H**</td><td>**Y,O,B**; I</td><td>5</td><td>0.0125</td></tr>
<tr><td></td><td></td><td></td><td></td><td></td><td>**+H**</td><td>**O**; S<I</td><td>5</td><td>0.0063</td></tr>
<tr><td>**25444**</td><td>Q568E7_DANRE</td><td>5.6e-35</td><td>45.4%</td><td>Similar to Zgc:110339; domains: transmembrane, short_chain_dehydrogenase; Lotgi1|131045 (5.6e-35)</td><td>**-H**</td><td>**Y,O,B**; I</td><td>3</td><td>0.0023</td></tr>
<tr><td></td><td></td><td></td><td></td><td></td><td>**+H**</td><td>**Y,B**; I</td><td>3</td><td>0.0006</td></tr>
<tr><td>**25522**</td><td>K1RCB4_CRAGI
(aa28-746)</td><td>1.8e-24</td><td>26.0%</td><td>Similar to von Willebrand factor D and EGF domain-containing protein; domains: VWD, EGF; Lotgi1|158683 (5.6e-76)</td><td>**-H**</td><td>**Y,O,B**; S<I</td><td>16</td><td>0.0155</td></tr>
<tr><td></td><td></td><td></td><td></td><td></td><td>**+H**</td><td>**Y,O,B**; S<I</td><td>19</td><td>0.0218</td></tr>
<tr><td>**256482**</td><td></td><td></td><td></td><td>Uncharacterized; domains: FN3/IG</td><td>**-H**</td><td>**Y,O**; S<I</td><td>1</td><td>0.0053</td></tr>
<tr><td></td><td></td><td></td><td></td><td></td><td>**+H**</td><td>**Y,O**; S<I</td><td>4</td><td>0.0710</td></tr>
<tr><td>**25670**</td><td>Q32NX0_XENLA</td><td>1.7e-52</td><td>46.0%</td><td>Uncharacterized; domains: Peptidase_C26/γ-glutamyl hydrolase, transmembrane; Lotgi1|108329 (8.0e-76)</td><td>**-H**</td><td>**Y,O**; I</td><td>2</td><td>0.0092</td></tr>
<tr><td></td><td></td><td></td><td></td><td></td><td>**+H**</td><td>**Y,O**; S<I</td><td>3</td><td>0.0364</td></tr>
<tr><td>**25804**
36369</td><td>Q9BKQ1_APLCA
(aa348-735)
(aa27-354)</td><td>1.2e-59
1.5e-58</td><td>44.6%
45.1%</td><td>NCAM-related adhesion molecule; domains: IG, signal peptide; Lotgi1|230852 (4.7e-44)/ Lotgi1|51189 (2.1e-19)</td><td>**-H**</td><td>-
Y,O,B; S<I</td><td>-
5</td><td>-
0.0173</td></tr>
<tr><td></td><td></td><td></td><td></td><td></td><td>**+H**</td><td>**Y**; I
Y,O,B; S<I</td><td>2
6</td><td>0.0006
0.0321</td></tr>
<tr><td>**25826**</td><td>Q45RT8_BIOGL</td><td>1.4e-75</td><td>81.4%</td><td>Fructose-bisphosphate aldolase; Lotgi1|217342 (2.0e-97)</td><td>**-H**</td><td>**Y,O**; I</td><td>2</td><td>0.0019</td></tr>
<tr><td></td><td></td><td></td><td></td><td></td><td>**+H**</td><td>-</td><td>-</td><td>-</td></tr>
<tr><td>**258525**</td><td>Q5DL74_BIOGL</td><td>6.4e-5</td><td>31.3%</td><td>Similar to Calcium binding protein 1; domains: signal peptide, EFh; Lotgi1|236429 (6.8e-7)</td><td>**-H**</td><td>**Y,O,B**; S,I</td><td>3</td><td>0.0368</td></tr>
<tr><td></td><td></td><td></td><td></td><td></td><td>**+H**</td><td>-</td><td>-</td><td></td></tr>
</table>

**25891**	J7QJT8_PATVU	6.3e-25	34.7%	Carbonic anhydrase; domains: signal peptide, αCA; Lotgi1|144122 (2.4e-65)	**-H**	**Y,O,B**; S<I	36	0.2168
					**+H**	**Y,O,B**; S<I	42	0.5870
**25994**	044341_HALRU (aa788-865)	0.0086	38.6%	Similar to lustrin A; signal peptide; 12% C, 10% L, 10% T	**-H**	**Y,O**; I	1	0.0092
					**+H**	**Y,O,B**; S<I	2	0.0105
**261700**				Uncharacterized; domain: transmembrane; 12% S	**-H**	**Y,B**; S,I	1	0.0009
					**+H**	**Y,B**; S	2	0.0013
**263**				Uncharacterized, many gaps in sequence! only 1 peptide possible!	**-H**	**Y,O,B**; S,I	1	0.0467
					**+H**	**Y,O,B**; S<I	1	0.2115
**263455**				Uncharacterized; 20% Q, very Q-rich N-term	**-H**	**Y,O**; S>I	1	0.0004
					**+H**	**Y**; S,I	2	0.0001
**2636**	P3476-2 (aa378-714)	4.6e-6	27.2%	Similar to Transmembrane cell adhesion receptor mua-3 (MUA_CAEEL); domain: VWA; Lotgi1|234764 (1.7e-10)	**-H**	**-**	-	-
					**+H**	**O**; I	3	0.0007
**26404**	B4N170_DROWI (aa1001-2481)	1.6e-89	29.8%	Similar to GK24802; domains: Sushi/SCR(CCP, EGF, Hyalin, GCC2_GCC3; Lotgi11|230171 (0e0)	**-H**	**-**	-	-
					**+H**	**Y**; I	2	0.0003
**26435**				Uncharacterized; 10% A, 13% S, 16% T	**-H**	**Y,O,B**; S>I	5	0.0356
					**+H**	**Y,O,B**; S>I	4	0.0650
**264452**	K1Q2S1-CRAGI (aa184-400)	2.6e-5	24.3%	Similar to cartilage matrix protein; domain: CBM_14/CHIT_BIND_II (~aa1-70)	**-H**	**Y,O**; S	2	0.0041
					**+H**	**Y,O,B**; S>I	3	0.0053
**26574**	R7TH93_9ANNE (aa1-675)	4.4e-145	51.9%	Uncharacterized/vacuolar-type H(+)-ATPase; domain: V_ATPase_I; Lotgi1|108161 (0e0)	**-H**	**Y,O,B**; I	7	0.0030
					**+H**	**Y,O,B**: S<I	14	0.0328
**2666**	A7RH56_NEMVE (aa212-367)			Uncharacterized; domain: peptidase_A1/cathepsin D; Lotgi1|173095 (1.4e-63)	**-H**	**Y**; I	2	0.0006
					**+H**	**Y,O**; I	4	0.0017
**26661**	E6Y6Q1_APLCA	2.3e-103	72.8%	Gelsolin; Lotgi1|214936 (5.0e-139)	**-H**	**Y,O,B**; I	5	0.0025
					**+H**	**Y,O,B**: S<I	10	0.0216

<table>
<tr><td rowspan="2">2668</td><td rowspan="2"></td><td rowspan="2"></td><td rowspan="2"></td><td rowspan="2">Uncharacterized; domains: Sushi/SCR/CCP, signal peptide; 10% A, 13% G, 10% S; Lotgi1|197362 (4.5e-11)</td><td>-H</td><td>Y,O,B; S>I</td><td>12</td><td>3.8981</td></tr>
<tr><td>+H</td><td>Y,O,B; S>I</td><td>13</td><td>3.8723</td></tr>
<tr><td rowspan="2">2678</td><td rowspan="2">R4WRF7_9HEMI</td><td rowspan="2">1.2e-37</td><td rowspan="2">79.5%</td><td rowspan="2">V-type proton ATPase subunit F; Lotgi1|205831 (2.9e-54)</td><td>-H</td><td>-</td><td>-</td><td>-</td></tr>
<tr><td>+H</td><td>Y; I</td><td>3</td><td>0.0107</td></tr>
<tr><td rowspan="2">269</td><td rowspan="2"></td><td rowspan="2"></td><td rowspan="2"></td><td rowspan="2">Uncharacterized; 18% G</td><td>-H</td><td>Y,O,B; S<I</td><td>8</td><td>0.6532</td></tr>
<tr><td>+H</td><td>Y,O,B; S<I</td><td>8</td><td>0.4419</td></tr>
<tr><td>2692</td><td>M4VQH6_APLCA</td><td>2.0e-107</td><td>79.2%</td><td>GAPDH; almost the same</td><td>-H</td><td>Y,O,B; I</td><td>8</td><td>0.0123</td></tr>
<tr><td>2985</td><td></td><td>1.6e-110</td><td>81.9%</td><td>Lotgi1|222542 (1.8e-108)</td><td>+H</td><td>Y,O,B; S<I</td><td>12</td><td>0.0177</td></tr>
<tr><td rowspan="2">27174</td><td rowspan="2">CCAP_CONVL (aa28-100)</td><td rowspan="2">1.1e-10</td><td rowspan="2">54.7%</td><td rowspan="2">aa80-164 similar to conoaCAP; domains: CCAP, signal peptide; Lotgi1|163724 (4.7e-6)</td><td>-H</td><td>Y,O; S,I</td><td>2</td><td>0.0015</td></tr>
<tr><td>+H</td><td>O,B; S>I</td><td>2</td><td>0.0025</td></tr>
<tr><td rowspan="2">272613</td><td rowspan="2"></td><td rowspan="2"></td><td rowspan="2"></td><td rowspan="2">Uncharacterized; domain: transmembrane</td><td>-H</td><td>O,B; S>I</td><td>1</td><td>0.0316</td></tr>
<tr><td>+H</td><td>Y,O,B; S>I</td><td>2</td><td>0.0147</td></tr>
<tr><td rowspan="2">273</td><td rowspan="2">M4AKV7_XIPMA</td><td rowspan="2">9.5e-35</td><td rowspan="2">47.0%</td><td rowspan="2">Uncharacterized/similar to sialic acid-binding lectin; domain: fibrinogen_C; Lotgi1|228333 (2.0e-30)</td><td>-H</td><td>Y,O,B; I</td><td>2</td><td>0.0065</td></tr>
<tr><td>+H</td><td>O,B; I</td><td>6</td><td>0.0475</td></tr>
<tr><td rowspan="2">27370</td><td rowspan="2">F1NCJ8_CHICK</td><td rowspan="2">4.6e-35</td><td rowspan="2">35.8%</td><td rowspan="2">Uncharacterized; domain: methyltransferase; Lotgi1|167413 (4.7e-96)</td><td>-H</td><td>-</td><td>-</td><td>-</td></tr>
<tr><td>+H</td><td>Y; S<I</td><td>4</td><td>0.0025</td></tr>
<tr><td rowspan="2">2744</td><td rowspan="2"></td><td rowspan="2"></td><td rowspan="2"></td><td rowspan="2">Uncharacterized; 10% K, 10% S</td><td>-H</td><td>Y,O,B; S<I</td><td>10</td><td>0.6080</td></tr>
<tr><td>+H</td><td>Y,O,B; S<I</td><td>14</td><td>0.9048</td></tr>
<tr><td rowspan="2">27726</td><td rowspan="2">E7EX29_HUMAN</td><td rowspan="2">6.0e-61</td><td rowspan="2">66.7%</td><td rowspan="2">14-3-3 ζ/δ ; shares peptides with other 14-3-3 proteins; Lotgi1|205379 (8.7e-83)</td><td>-H</td><td>Y,O,B; I</td><td>2</td><td>0.0017</td></tr>
<tr><td>+H</td><td>Y,O,B; I</td><td>5</td><td>0.0054</td></tr>
<tr><td rowspan="2">277362</td><td rowspan="2">K1RN67_CRAGI (aa971-1220)</td><td rowspan="2">7.3e-37</td><td rowspan="2">42.6%</td><td rowspan="2">Similar to Receptor-type tyrosine-protein phosphatase T; Lotgi1|102205 (5.3e-53)</td><td>-H</td><td>-</td><td>-</td><td>-</td></tr>
<tr><td>+H</td><td>Y,O; I</td><td>2</td><td>0.0020</td></tr>
<tr><td rowspan="2">27801</td><td rowspan="2">K1PZK2_CRAGI</td><td rowspan="2">3.3e-53</td><td rowspan="2">74.9%</td><td rowspan="2">Putative myosin regulatory light chain; domains: EFh; Lotgi1|190618 (6.8e-89)</td><td>-H</td><td>Y,O,B; I</td><td>1</td><td>0.0017</td></tr>
<tr><td>+H</td><td>Y,B; I</td><td>1</td><td>0.0019</td></tr>
</table>

**27819**	G1LA32_AILME	9.2e-56	44.4%	Uncharacterized; domain: serpin; Lotgi1|195172 (1.7e-48)	**-H**<br>**+H**	**Y,B**; I<br>**Y,O**; I	2<br>4	0.0006<br>0.0016
**27977**	H9KHU2_APIME	5.0e-35	34.3%	Uncharacterized/similar to membrane metallo-endopeptidase-like; domain: peptidase M13; Lotgi1|165736 (7.2e-53)	**-H**<br>**+H**	**Y,O,B**; I<br>-	3<br>-	0.0034<br>-
**28132**	C3XVU1_BRAFL (aa512-857)	9.8e-18	28.5%	Uncharacterized/similar to fibrillin; domains: EGF; 11% C, 11% G, 10% S; Lotgi1|230443 (1.4e-29)	**-H**<br>**+H**	**Y,O,B**; I<br>-	3<br>-	0.0024<br>-
**2836**	K1QWX2_CRAGI	1.3e-91	78.6%	60S acidic ribosomal protein P0; Lotgi1|237709 (2.0e-144)	**-H**<br>**+H**	**Y,O,B**; S<I<br>**Y, O**; I	4<br>5	0.0034<br>0.0048
**28471**	K1PQF1_CRAGI	1.5e-60	33.0%	Similar to neural cell adhesion molecule L1; domains: signal peptide, IG, transmembrane segment; Lotgi1|239531 (7.3e-61)	**-H**<br>**+H**	**Y,B**; I<br>**Y,B**; I	2<br>1	0.0010<br>0.0004
**2855**	A4LAB0_DORPE	7.6e-52	43.2%	Na^+/K^+ ATPase beta subunit; Lotgi1|213719 (3.8e-81)	**-H**<br>**+H**	**Y,O,B**; S<I<br>**Y,O**; S,I	2<br>5	0.0096<br>0.0182
**2858**	H2ZUY5_LATCH (aa165-414)	5.5e-41	44.6%	Uncharacterized/similar to adipocyte plasma membrane-associated protein; domains: TolB_like/strictosidine:synthase_related; Lotgi1|231719 (3.8e-81)	**-H**<br>**+H**	**Y,O,B**; S<I<br>**Y,O,B**; S<I	8<br>11	0.0388<br>0.1403
**28685**	H1AFK3_APLKU	0e0	58.5%	Beta-D-mannosidase; domains: transmembrane, glycoside_hydrolase_fam_2; Lotgi1|212346 (0e0)	**-H**<br>**+H**	-<br>**Y,O**; I	-<br>3	-<br>0.0002
**28902**	B6DYD6_PROCL	2.7e-50	100%	Calmodulin; Lotgi1|216228 (1.1e-94)	**-H**<br>**+H**	**Y,O,B**; S<I<br>**Y,O,B**; S<I	5<br>3	0.0337<br>0.0285
**28994**	K1QPM9_CRAGI	1.5e-22	45.6%	Similar to fatty acid-binding protein, brain; Lotgi1|158663 (2.9e-26)	**-H**<br>**+H**	**Y,O,B**; S,I<br>**Y,O,B**; S,I	5<br>7	0.1948<br>0.2396
**29148**	K1S5N3_CRAGI	1.3e-103	53.4%	Hexokinase type 2; Lotgi1|233221 (0e0)	**-H**<br>**+H**	-<br>**Y**; I	-<br>3	-<br>0.0005
**29171**	G0YL88_MYTGA	8.8e-123	47.0%	MACPF domain-containing protein; domains: MACPF_1/perforin, signal peptide	**-H**<br>**+H**	**Y,O,B**; I<br>**Y,O,B**; S<I	7<br>9	0.0148<br>0.0229

	G0YL88_MYTGA			MACPF domain-containing protein; fragment;				
**29173**	(aa359-557)	7.3e-49	52.3%		**-H**	**Y,O,B**; I	3	0.0128
**59951**	(aa24-171)	8.9e-22	39.7%	signal peptide		**Y,O,B**; I	3	0.0020
					**+H**	**Y,O,B**; S<I	5	0.0293
						**Y,O,B**; S<I	3	0.0096
**29423**	Q8MY76_9BIVA	1.3e-140	37.9%	aa209-1416 similar to Se-cadherin, fragment; Lotgi1|167053 (0e0)	**-H**	**-**	-	-
					**+H**	**Y,O; I**	4	0.0005
**29324**	E1B2D0_9BIVA (aa12-401)	2.5e-135	71.7%	Catalase; Lotgi1|13141 (0e0)	**-H**	**Y,O,B**; S<I	13	0.0149
					**+H**	-	-	-
**29426**	K1QGP8_CRAGI (aa188-2080)	7.9e-65	29.7%	aa304-2129 similar to atrial natriuretic peptide-converting enzyme; domains: MAM, trypsin, LDLRA, frizzled; Lotgi1|167919 (0e0)	**-H**	**Y,O**; I	1	<0.0001
					**+H**	**Y,O**; I	5	0.0004
**29558**	F6XNT9_XENTR	4.9e-139	50.3%	β-galactosidase; transmembrane/signal peptide; Lotgi1|236200 (0e0)	**-H**	**-**	-	-
					**+H**	**Y,O**; I	3	0.0005
**2957**	K1PVC1_CRAGI	1.1e-38	37.9%	aa121-395 similar to Carbohydrate sulfotransferase 11; domains; transmembrane/signal sequence, sulfotransf_2; Lotgi1|166777 (2.6e-88)	**-H**	**-**	-	-
					**+H**	**Y,O**; S<I	3	0.0013
**297**	G3FPE5_HELLU (aa2924-3142)	1.0e-70	77.3%	Similar to β-hemocyanin (probably not identical!), shares peptide with contig_67	**-H**	**Y,O,B**; I	3	0.0068
					**+H**	**Y,O,B**; I	3	0.0082
**2977**	Q8I8Y1_MANSE (aa260-744)	7.2e-103	45.5%	; domains: cupredoxin; Lotgi1|231844 (7.0e-152)	**-H**	**B**; I	2	0.0007
					**+H**	-	-	-
	Q000T7_PINFU			ATP synthase subunit α; same protein with	**-H**	**Y,O,B**; S<I	9	0.0285
**3002**	(aa1-302)	8.4e-114	84.8%	microheterogeneities? Lotgi1|206617 (0e0)/178690	**+H**	**Y,O,B**; S<I	11	0.0384
**39893**	(aa1-146)	4.5e-37	75.5%	(2.1e-64)		**Y**; I	2	0.0022
**3007**	K1QZJ5_CRAGI (aa20-534)	8.8e-80	42.8%	Similar to glucose dehydrogenase [acceptor]; Lotgi1|169492 82.0e-70)	**-H**	**Y,O**; I	2	0.0003
					**+H**	**Y,O**; I	3	0.0004
**30629**	R7TIU6_9ANNE	1.5e-95	60.9%	Uncharacterized/Vesicle amine transport protein; domains: ADH, GroES_like; Lotgi1|219806 (8.6e-115)	**-H**	**-**	-	-
					**+H**	**Y**; I	2	0.0003

**3069**	K1QXS6_CRAGI (aa1-303)	5.7e-35	53.9%	Similar to heterogeneous nuclear ribonucleoprotein A2-like protein 1; Lotgi1|166794 (8.5e-68)	**-H**	**Y,O**; I	2	0.0003
					**+H**	**Y**; I	2	0.0002
**3077**	B0WIN7_CULQU (aa151-335)	7.4e-22	35.2%	Similar to peritrophic membrane chitin binding protein; domains:chitin deacetylase; Lotgi1|153749 (2.8e-30)	**-H**	**Y,O,B**; S<I	6	0.0495
					**+H**	**Y,O,B**; S<I	7	0.0548
**30790**	K1PNR3_CRAGI	0e0	88.3%	Clathrin heavy chain 1; Lotgi1|233411 (0e0)	**-H**	**O,B**; S>I	5	0.0002
					**+H**	**Y,O,B**; I	6	0.0001
**31026**	A7SDH9_NEMVE (aa254-665)	1.7e-76	48.3%	Uncharacterized; domains: phospholipase_C, CH; Lotgi1|201534 (7.4e-161)	**-H**	**-**	-	-
					**+H**	**Y**; I	3	0.0008
**3107**				Uncharacterized; signal peptide; 12% R, 11% S, 11% T, 10% V	**-H**	**Y,O,B**; I	5	0.0116
					**+H**	**Y,O,B**; S<I	5	0.0324
**31170**	K7S1Q8_NASVI (aa431-797)	3.2e-37	35.0%	Uncharacterized; domains: CMB_14/CHIT_BIND_II, signal peptide; Lotgi1|153749 (7.4e-45)	**-H**	**Y,O,B**; S<I	13	0.0769
					**+H**	**Y,O,B**; S<I	17	0.1140
**31301**	G3VTN2_SARHA	7.2e-30	29.9%	Uncharacterized/similar to renin receptor; domains: renin_receptor, transmembrane; Lotgi1|211795 (1.1e-71)	**-H**	**Y,O,B**; S<I	5	0.0030
					**+H**	**Y,O**; S<I	7	0.0173
**313922**				Uncharacterized; signal peptide	**-H**	**Y,O,B**; S<I	3	0.0306
					**+H**	-	-	-
**31433**	I1VYY1_MYTTR	3.2e-158	77.7%	Isocitrate dehydrogenase; Lotgi1|220933 (0e0)	**-H**	**Y,O**; I	2	0.0006
					**+H**	**Y,O,B**; I	4	<0.0001
**32001**	K1PQI4_CRAGI	1.1e-31	55.2%	Enolase-phosphatase E1; Lotgi1|168703 (4.5e-57)	**-H**	**Y,O,B**; I	2	0.0037
					**+H**	**Y,O,B**; I	3	0.0025
**320130**	K1QC92_CRAGI (aa320-509)	4.7e-41	45.8%	Similar to Sulfhydryl oxidase 1; domain: ERV_ALR; Lotgi1|237352 (1.5e-65)	**-H**	**-**	-	-
					**+H**	**Y,O**; S<I	2	0.0036
**32297**				Uncharacterized; signal peptide; 14% G, 10% P	**-H**	**Y,O,B**; S>I	2	0.2826
					**+H**	**Y,O,B**; S>I	2	0.2291

**3243**				Uncharacterized; domains: signal peptide, H-type_lectin	**-H**	**Y,O,B**; I	2	0.0044
					**+H**	**Y,O,B**; S<I	3	0.0098
**324404**	K1PUB4_CRAGI	7.8e-13	37.3%	Similar to ADAM family mig-17; domain: peptidase M12B; Lotgi1|228200 (8.0e-29)	**-H**	**Y,O,B**; I	2	0.0088
					**+H**	**Y,O,B**; S<I	2	0.0348
**32583**	A1L3T3_MOUSE	7.8e-127	54.6%	Similar to N-sulfoglucosamine sulfohydrolase/heparin N-sulfatase; domains: sulfatase, signal peptide; Lotgi1|175173 (0e0)	**-H**	**Y,O,B**; I	6	0.0030
					**+H**	**Y,O,B**; S<I	8	0.0137
**32596**	Q86DI0_APLCA	1.3e-68	97.7%	Rac; domain: Ras/Rho; Lotgi1|230301 (1.2e-115)	**-H**	-	-	-
					**+H**	**Y,O**; I	2	0.0014
**32713**				Uncharacterized; domains: EFh	**-H**	**Y,O,B**; S<I	3	0.0093
					**+H**	**Y,O**; S<I	4	0.0081
**32845**				Uncharacterized; domain: ShK	**-H**	**Y,O,B**; S<I	4	0.0010
					**+H**	**Y,O,B**; S>I	8	0.0030
**32858**	K1PXG8_CRAGI	1.1e-157	99.1%	Serine/threonine-protein phosphatase; shares peptide with contig_39278	**-H**	**Y,O**; S,I	2	0.0005
					**+H**	**Y,O**; I	2	0.0009
**33806**	A7SFM8_NEMVE	7.6e-107	52.3%	Aldehyde dehydrogenase; Lotgi1|229102 (0e0)	**-H**	-	-	-
					**+H**	**Y,O**; I	4	0.0011
**34386**	K1PD20_CRAGI	7.6e-70	46.3%	Similar to Di-N-acetylchitobiase; domains: chitinase_II, signal peptide; Lotgi1|209107 (7.1e-99)	**-H**	**Y,O**; I	3	0.0006
					**+H**	**Y,O**; S<I	16	0.0076
**34455**	H9LSX1_CRAGI	1.6e-90	60.6%	Cathepsin B; Lotgi1|197490 (6.4e-137)	**-H**	**Y,O**; I	2	0.0009
					**+H**	**Y,O**; S<I	5	0.0122
**3453**	L7NCQ8_9CAEN	3.1e-51	56.6%	Mu-class glutathione S-transferase; domains: GST_N, GST_C; Lotgi1|233779 (1.5e-68)	**-H**	-	-	-
					**+H**	**Y**; I	2	0.0002
**34537**	C3YKY0_CRAGI	7.1e-15	36.6%	Uncharacterized; Lotgi1236424 (1.2e-20)	**-H**	**Y,O,B**; S,I	2	0.0054
					**+H**	-	-	-

**34569**	S4R9R4_PETMA	6.2e-82	77.1%	Uncharacterized/V-type proton ATPase subunit d 1; Lotgi1|108853 (1.8e-137)	**-H**	**Y,O**; I	2	0.0015
					**+H**	**Y,O**; S<I	6	0.0143
**34894**	B3RQ10_TRIAD (aa417-1735)	2.6e-26	24.2%	Uncharacterized; domains: EGF, VWD, extracellular (IPR003886), transmembrane; Lotgi1|166811 (1.9e-66)	**-H**	**-**	-	-
					**+H**	**Y**; I	5	0.0012
**34916**	B7PSF8_IXOSC (aa504-655)	3.6e-12	31.2%	Similar to hemocyte protein-glutamine γ-glutamyltransferase; domain: transglutam_C; IG; Lotgi1|231723 (4.2e-33)	**-H**	**Y,O,B**; I	2	0.0020
					**+H**	**Y,O,B**; S<I	5	0.0089
**35033**	B0JZP5_DANRE	8.1e-55	41.3%	DNAse 2; Lotgi1|125778 (8.9e-64)	**-H**	**Y,O,B**; I	2	0.0013
					**+H**	**Y,O**; S>I	2	0.0047
**35115**	B6RB39_HALDI	4.7e-12	26.2%	Similar to X-box binding protein; domain: ependymin, signal peptide; Lotgi1|233583 (1.1e-15)	**-H**	**Y,B**; I	1	0.0063
					**+H**	**Y,B**; S,I	1	0.0021
**351458**				Uncharacterized; domains: PDZ; 13% S	**-H**	**Y,O,B**; S<I	1	0.0107
					**+H**	**Y,O,B**; I	1	0.0041
**353**	C3YD43_BRAFL	1.7e-30	69.3%	Ribosomal protein L23; Lotgi1|179313 (5.3e-55)	**-H**	**Y,B**; I	1	0.0024
					**+H**	**Y,B**; I	1	0.0024
**353551**	Q32R30_EPTBU (aa64-160)	6.9e-6	38.8%	aa4-105 similar to Variable lymphocyte receptor A; domains: Leu-rich repeats; Lotgi1|171753 (1.0e-11)	**-H**	**-**	-	-
					**+H**	**Y,O**; I	2	0.0007
**358462**	K1QPM9_CRAGI	8.1e-4	27.9%	Similar to fatty acid binding protein; domain: calycin_like; 15% T, 12% V	**-H**	**Y,O,B**; I	4	0.0078
					**+H**	-	-	-
**35849**	R7VAX7_9ANNE (aa9-485)	5.1e-117	54.4%	β-hexosaminidase; shares 2 peptides with contig_11304;domains:signal sequence, glycol_hydro_20; Lotgi1|120422 (5.5e-177)	**-H**	**-**	-	-
					**+H**	**Y,O**; S<I	3	0.0011
**35852**				Uncharacterized; 11% A, 11% P	**-H**	**Y,O,B**; S,I	13	0.7909
					**+H**	**Y,O,B**; S>I	14	1.4581
**359**	IFEA_HELAS (aa444-551)	9.4e-51	93.1%	Non-neuronal cytoplasmic intermediate filament protein; Lotgi1|156050 (6.6e-11)	**-H**	**Y,O,B**; I	4	0.0702
					**+H**	**Y,O,B**; S<I	4	0.0935

**36266**	R7TC05_9ANNE	4.3e-167	56.0%	Uncharacterized/β-glucuronidase; domains: glycoside_hydrolase_family_2, signal peptide; Lotgi1|165508 (0e0)	**-H**	**Y,O,B**; I	1	0.0004
					**+H**	**Y**; I	1	0.0004
**3631**	R7UU94_9ANNE (aa185-275)	1.2e-11	42.9%	Uncharacterized/α-carbonic anhydrase; domain: α-CA; Lotgi1|163324 (6.0e-13)	**-H**	**Y,O,B**; I	3	0.0374
					**+H**	**Y,O,B**; S<I	2	0.0702
**36446**	K1QA50_CRAGI (aa22-427)	8.4e-115	65.3%	V-type proton ATPase subunit H; Lotgi1|207139 (0e0)	**-H**	**Y,O**; I	7	0.0016
					**+H**	**Y,O**; S<I	14	0.0220
**36714**				Uncharacterized; domains: FN3	**-H**	**-**	-	-
					**+H**	**Y,O**; S<I	2	0.0230
**37078**	K1PXB8_CRAGI (aa25-554)	1.6e-28	25.4%	aa52-552 similar to disintegrin and metalloproteinase with thrombospondin motifs 1; domains: signal peptide, peptidase_M12B; Lotg1|171445 (1.4e-50)	**-H**	**-**	-	-
					**+H**	**Y,O**; S,I	2	0.0003
**3720**	K1PVW0_CRAGI (aa197-410)	8.8e-76	85.0%	S-adenosylmethionine synthetase; Lotgi1|141813 (5.8e-115)	**-H**	**Y,O,B**; S<I	1	0.0027
					**+H**	**Y,O,B**; I	1	0.0024
**37240**				Uncharacterized; signal peptide	**-H**	**Y,B**; S,I	2	0.0042
					**+H**	**Y,B**; S	2	0.0032
**37423**	B4LLG4_DROVI	4.4e-44	62.2%	GJ22316/FKBP-type peptidyl-prolyl cis-trans isomerase; domans: signal peptide, PPI_FKBP_type; Lotgi1|238515 (1.3e-54)	**-H**	**Y,O**; I	1	0.0010
					**+H**	**Y,O**; S	1	0.0018
**3748**	Q0KHB6_CRAGI	2.2e-172	69.2%	Pyruvate kinase; Lotgi1|199626 (0e0)	**-H**	**-**	-	-
					**+H**	**Y,O,B**; I	19	0.0106
**3773**	Q45RT8_BIOGL (aa1-175)	8.2e-58	79.4%	Fructose-bisphosphate aldolase; Lotgi1|217342 (5.2e-81)	**-H**	**Y,O,B**; I	4	0.0048
					**+H**	**Y,O**; I	7	0.0221
**3799**	K1PJE5_CRAGI (aa119-347)	2.4e-43	49.1%	Pancreatic triacylglycerol lipase (Fragment); Lotgi1|216931 (3.0e-71)	**-H**	**Y,O**; I	1	0.0003
					**+H**	**Y,O**; S<I	5	0.0010

**38327**	K1PTI6_CRAGI	3.2e-181	72.1%	Glucose-6-phosphate isomerase; Lotgi1|217535 (0e0)	**-H**	**-**	-	-
					**+H**	**Y,B**; I	2	0.0007
**38376**	K1RWX7_CRAGI	3.4e-112	28.3%	Similar to metabotropic glutamate receptor 3; domains: signal peptide, transmembrane, GPCR_3, ANF_receptor, periplasmic_binding_like_1; Lotgi1|152964 (0e0)	**-H**	**-**	-	-
					**+H**	**Y,O**; I	6	0.0002
**38447**	K1QHL0_CRAGI (aa10-220)	3.3e-31	42.9%	Allene oxide synthase-lipoxygenase protein; Lotgi1|171809 (9.8e-63)	**-H**	**B**; I	1	<0.0001
					**+H**	**Y,O**; I	3	0.0035
**38612**	G3NQT9_GASAC (aa280-662)	6.2e-131	77.9%	Uncharacterized/succinate dehydrogenase [ubiquinone] flavoprotein subunit, mitochondrial; Lotgi1221335 (0e0)	**-H**	**Y,O,B**; I	3	0.0012
					**+H**	**Y,O,B**; I	3	0.0022
**3868**	D7QYI1_BIOGL (aa378-661)	8.7e-51	48.6%	Thioester-containing protein 1.5; domain: A2M_N_2; Lotgi1|162872 (8.0e-38); see also contig_11338 and 21999	**-H**	**Y,O,B**; S<I	8	0.0522
					**+H**	**Y,O,B**; S<I	7	0.0172
**38710**	K1P9R1_CRAGI	2.3e-83	50.6%	Anhydro-N-acetylmuramic acid kinase; Lotgi1|122845 (1.9e-147)	**-H**	**-**	-	-
					**+H**	**Y**; I	3	0.0012
**3883**	K1Q9V3_CRAGI	5.9e-190	86.2%	V-type proton ATPase catalytic subunit A; Lotgi1|185986 (0e0)	**-H**	**Y,O,B**; S<I	19	0.0247
					**+H**	**Y,O,B**; S<I	27	0.1671
	K1R401_CRAGI			Spectrin α-chain; Lotgi1|202396 (0e0)	**-H**	**-**	-	-
**38980**	(aa1545-2108)	2.1e-151	79.3%		**+H**	**Y,B**; I	5	0.0006
**39106**	(aa1-381)	5.4e-53	59.5%			**Y,O**; I	3	0.0002
**39018**				Uncharacterized; signal peptide	**-H**	**Y,O,B**; I	2	0.0076
					**+H**	**Y,O,B**; S<I	3	0.0130
**3938**				Uncharacterized; signal peptide	**-H**	**Y,O,B**; S>I	2	0.5678
					**+H**	**Y,O,B**; S>I	2	0.5817
**3942**	K1QHY1_CRAGI (aa112-696)	2.7e-77	36.2%	Similar to eosinophil peroxidase; domains: Haem_peroxidase, signal peptide; Lotgi1|233510 (7.3e-75); see also contig_11883	**-H**	**Y,O,B**; I	5	0.0004
					**+H**	-	-	-

**394728**				Uncharacterized	**-H**	**Y,O,B**; S<I	4	0.0089
					**+H**	-	-	-
**401**	F7A172_CIOIN	2.2e-91	75.0%	Ribosomal protein L5 ; Lotgi1|171614 (8.4e-113)	**-H**	-	-	-
					**+H**	**Y,B**; I	2	0.0021
**4048**	K1QX26_Cragi (aa608-1082)	7.5e-122	74.0%	Endoplasmin; Lotgi1|197084 (0e0)	**-H**	**Y,O,B**; S<I	3	0.0016
					**+H**	**Y,O,B**; I	4	0.0014
**40858**	B1N694_HALDI	1.5e-69	89.6%	Thioredoxin peroxidase 2; Lotgi1|150310 (2.8e-105)	**-H**	**Y,O,B**; S<I	2	0.0242
					**+H**	**Y,O,B**; S<I	2	0.0290
**4111**	K1RG91_CRAGI	1.6e-25	50.6%	Transgelin-2; Lotgi1|152623 (8.2e-56); very similar entries but different peptides at the same place! Share 1 peptide.	**-H**	**Y,B**; I	2	0.0012
**416**		2.1e-29	51.5%			**Y,B,O**; I	3	0.0065
					**+H**	-	-	-
**41145**	L5KXM7_PTEAL (aa54-256	5.1e-79	83.7%	α3-tubulin; Lotgi1|201713 (3.8e-111)	**-H**	**Y,B**; I	1	0.0009
					**+H**	**Y**; I	1	0.0008
**41290**	R7TDC7_9ANNE (aa2-214)	1.4e-33	52.5%	Uncharacterized/similar to hsc70-interacting protein; domain: TPR_2; Lotgi1|64759 (1.6e-68)	**-H**	**Y,B**; I	1	0.0004
					**+H**	**Y**; I	2	0.0018
**4262**	R7TCA8_9ANNE (aa70-395)	2.0e-44	51.0%	Uncharacterized/similar to Heterogeneous nuclear ribonucleoprotein K (K1Q324_CRAGI); Lotgi1|121010 (8.3e-69)	**-H**	-	-	-
					**+H**	**Y,B**; I	2	0.0002
**41623**	K1R2C6_CRAGI	2.0e-107	52.7%	aa43-555 similar to Alkaline phosphatase, tissue-nonspecific isozyme; Lotgi1|236474 (1.9e-118)	**-H**	-	-	-
					**+H**	**Y,O**; I	8	0.0019
**4164**				Uncharacterized; limited similarity to UP2_HALAI (e-value 0.19; 28.1% identity)	**-H**	**Y,O,B**; S>I	2	1.4832
					**+H**	**Y,O,B**; S>I	3	2.0730
**41818**				Uncharacterized; signal peptide; 13% A, 10% D, 10% V	**-H**	**Y,O,B**; S,I	1	0.0129
					**+H**	**Y,O,B**; S<I	1	0.0128
**418581**	K1PII9_CRAGI (aa173-371)	2.3e-23	36.3%	Reelin; Lotgi1|228850 (5.2e-39)	**-H**	**Y,O**; I	1	0.0007
					**+H**	**Y,O**; I	1	0.0018

**4189**				Uncharacterized; fragment?	**-H**	**Y,O,B**; S,I	1	0.0745
					**+H**	**O,B**; I	1	0.0084
**41918**	K1S6N9_CRAGI (aa250-484)	6.6e-31	39.7%	Similar to lachesin; domain: IG; Lotgi1|162469 (7.3e-61)	**-H**	**Y,O**; I	1	0.0004
					**+H**	**Y,O**; S<I	1	0.0023
**42513**	G6D8P2_DANPL	1.8e-77	38.3%	Uncharacterized/similar to disintegrin and metalloproteinase domain containing protein; domains: peptidase_M12B, disintegrin, ADAM10, signal peptide; Lotgi1|199969 (0e0)	**-H**	**Y,O,B**; S>I	2	0.0016
					**+H**	**Y,O,B**; S>I	4	0.0040
**4265**	K1QTT1_CRAGI (aa2-147)	3.4e-28	51.0%	aa42-185 similar to uncharacterized; signal peptide; Lotgi1|232745 (6.7e-34)	**-H**	**-**	-	-
					**+H**	**Y,O**; I	2	0.0036
**4275**	C5IIM1_HALDV	1.3e-60	48.5%	Cathepsin L-like cysteine proteinase;domains: signal peptide, peptidase_C1A; see also contig_10485; Lotgi1|221163 (4.1e-79)	**-H**	**Y**; I	2	0.0005
					**+H**	**Y,O**; S<I	4	0.0041
**42852**	K1QK91_CRAGI	2.3e-152	51.6%	Prestin; domain: sulfate_anion_transporter; Lotgi1|53031 (0e0)	**-H**	**Y,O**; I	2	<0.0001
					**+H**	**Y**; I	5	0.0008
**43333**	K1QKM4_CRAGI (aa1-459)	1.7e-157	72.8%	Xylose isomerase; Lotgi1|202268 (0e0)	**-H**	**Y,O**; I	4	0.0005
					**+H**	**Y,O**; I	8	0.0038
**4367**	R7V5G0_9ANNE	1.7e-65	88.8%	Uncharacterized/Rab 7; Lotgi1|223383 (3.0e-126)	**-H**	**O**; S,I	2	0.0002
					**+H**	**Y,B**; I	3	0.0011
**4368**	K1PVA1_CRAGI (aa7-370)	1.9e-115	94.5%	Transitional endoplasmic reticulum ATPase; Lotgi1|216777 (0e0)	**-H**	**-**	-	-
					**+H**	**Y,B**; I	6	0.0020
**43829**	Q8I819_APLCA (aa26-153)	2.1e-13	37.4%	Uncharacterized/similar to ectonucleoside triphosphate diphosphohydrolase; domains: signal peptide, nucleoside phosphatase GDA1/CD39; Lotgi1|238045 (6.8e-11)	**-H**	**Y,O**; I	1	0.0012
					**+H**	**Y,O**; S<I	2	0.0040
**4386**	F2TX66_SALS5 (aa490-698)	4.5e-10	31.6%	Uncharacterized; domain: VWA; Lotgi1|123318 (2.8e-13)	**-H**	**Y,O,B**; S<I	3	0.0313
					**+H**	**O;B**; I	2	0.0008

**4405**	I3K648_ORENI	2.7e-61	66.4%	Uncharacterized/14-3-3 ; Lotgi1|207549 (6.1e-87); shares peptides with other 14-3-3 proteins	**-H**	**Y,O,B**; I	1	0.0020
					**+H**	**Y,O**; S<I	4	0.0064
**4409**	K1QXR1_CRAGI (aa277-772)	2.9e-51	37.2%	Putative tyrosinase-like protein tyr-3; Lotgi1|166196 (2-1e-92); see also contig_8371!	**-H**	**Y,O,B**; S<I	12	0.0684
					**+H**	**Y,O,B**; I	3	0.0075
**44723**	K1RBX7_CRAGI	2.9e-75	37.7%	Uncharacterized; signal peptide	**-H**	**Y,O,B**; I	8	0.0019
					**+H**	**Y,O,B**; S>I	16	0.0156
**44780**	R4G5H1_RH0PR	1.9e-101	76.8%	Arp2/3; Lotgi1|220774 (9.7e-168)	**-H**	**-**	-	-
					**+H**	**Y**; I	2	0.0002
**450**	68CYM6_PHYAT	9.9e-67	74.2%	G-type lysozyme;signal peptide; Lotgi1|110722 (6.9e-55)	**-H**	**Y,O,B**; S<I	5	0.2134
					**+H**	**Y,O,B**; S<I	5	0.3560
**45203**	E9GD19_DAPPU (aa1-482)	1.0e-107	58.2%	Coronin; Lotgi1|114901 (0e0)	**-H**	**Y,O,B**; I	2	0.0004
					**+H**	**Y,O**; I	4	0.0021
**45530**	B8YJJ3_LYMST	1.6e-109	83.8%	Retinal dehydrogenase; Lotgi1|192810 (1.1e-133)	**-H**	**Y**; I	1	0.0003
					**+H**	**Y**; I	8	0.0057
**4572**	GELS2_LUMTE (aa180-366)	5.0e-26	46.0%	Gelsolin-like protein 2; Lotgi1|192582 (2.4e-34)	**-H**	**Y,O,B**; S<I	4	0.0272
					**+H**	**Y,O,B**; I	5	0.0009
**45754**	K1R7T2_CRAGI	1.6e-152	82.2%	Isocitrate dehydrogenase [NADP]; Lotgi1|208961 (0e0)	**-H**	**-**	-	-
					**+H**	**Y,O,B**; I	2	0.0003
**459**				Uncharacterized; signal peptide; 14% Q, 11% M, 10% P	**-H**	**Y,O,B**; S<I	10	0.0135
					**+H**	**Y,O,B**; S<I	8	0.0076
**45900**	L7LZH1_9ACAR (aa6-375)	1.2e-46	41.6%	Uncharacterized; domains: PDZ; Lotgi1|237252 (5.5e-99)	**-H**	**Y,O,B**; I	3	0.0011
					**+H**	**Y**; I	7	0.0034
**4597**	T1DMA1_CROHD (aa344-715)	1.6e-88	57.1%	NADH-ubiquinone oxidoreductase 75 kDa subunit, mitochondrial-like protein; Lotgi1|206956 (5.6e-129)	**-H**	**-**	-	-
					**+H**	**Y**; I	2	0.0003

**46005**	K1Q014_CRAGI	3.3e-57	33.6%	Similar to Solute carrier family 22 member 21; Lotgi1|168961 (6.2e-74)	**-H**	**-**	-	-
					**+H**	**Y**; I	2	0.0014
**461**	K1QRL6_CRAGI	2.1e-55	46.7%	Similar to Methenyltetrahydrofolate synthetase domain-containing protein; Lotgi1|224471 (9.7e-87)	**-H**	**-**	-	-
					**+H**	**Y,B**; I	2	0.0003
**46246**	Q8IU44_LOLBL (aa41-279)	8.2e-27	64.2%	Myosinase-III; Lotgi1|209446 (7.1e-37)	**-H**	**Y,O**; S	1	0.0011
					**+H**	**Y,O**; S	1	0.0009
**46337**	O44343_BIOGL (aa5-232/389-521)	2.2e-78	65.0%	HSP70; Lotgi1|177837 (0e0); shares peptide with contig_695	**-H**	**Y,O,B**; S<I	3	0.0130
					**+H**	**Y,O,B**; S<I	4	0.0146
**4702**	G9K384_HALDV (aa96-403)	6.9e-53	44.5%	Similar to putative procollagen type VI α4, domain: VWA; Lotgi1|233111 (4.4e-46)	**-H**	**Y,B**; I	3	0.0005
					**+H**	**B**; I	3	0.0005
**47032**	K1Q531_CRAGI	1.5e-35	61.6%	Neural proliferation differentiation and control protein 1; domains: NPDC1, transmembrane; Lotgi1|232872 (8.4e-49)	**-H**	**Y,B**; S	2	0.0007
					**+H**	**Y,O**; S	1	0.0003
**47053**	R7VGB5_9ANNE (aa1-466)	1.1e-115	54.3%	Uncharacterized; domains: transglutaminase/γ-glutamyltransferase; Lotgi1|231723 (0e0)	**-H**	**Y,O,B**; I	2	0.0010
					**+H**	**Y,O**; I	9	0.0084
**47191**	K1PX68_CRAGI (aa211-671)	1.7e-22	29.8%	Similar to tyrosine-protein phosphatase non-receptor type 6; domains: signal peptide, PBP; Lotgi1|173822 (3.0e-27)	**-H**	**Y,O,B**; I	1	0.0007
					**+H**	**Y,O**; S<I	2	0.0027
**47261**	K1R091_CRAGI (aa89-695)	6.5e-111	47.5%	Similar to plectin-1; domain: SH3; Lotgi1|232953 (1.2e-176)	**-H**	**-**	-	-
					**+H**	**Y,O**; I	3	0.0002
**47483**	K1R973_CRAGI	4.7e-43	77.4%	Chloride intracellular channel exc-4; domain: GST_C; Lotgi1|189089 (5.2e-89)	**-H**	**Y,O**; I	4	0.0018
					**+H**	**Y,O**; S<I	8	0.0189
**4755**				Uncharacterized, domains: EGF_like, WAP; Lotgi1|153970 (4.8e-20)	**-H**	**Y,O,B**; S<I	14	0.0403
					**+H**	**Y,O,B**; S<I	16	0.0778
**47828**				Uncharacterized	**-H**	**Y,O,B**; I	4	0.0079
					**+H**	**Y,O,B**; S<I	4	0.0226

**47888**	B3MFN3_DROAN	1.5e-92	58.2%	Uncharacterized/V-type proton ATPase subunit C; Lotgi1|206255 (9.5e-141)	**-H**	**Y,O**; I	4	0.0007
					**+H**	**Y,O,B**; S<I	12	0.0140
**48400**	E9G2W4_DAPPU (aa1-848)	0.e0	59.7%	Uncharacterized/filamin; domains: filamin, CH; Lotgi1|158607 (0e0)	**-H**	**Y,O,B**; I	4	0.0008
					**+H**	**Y,O**; I	11	0.0023
**4865**	K1R1B6_CRAGI	1.7e-128	47.8%	D-glucuronyl C5-epimerase; Lotgi1|178848 (0e0)	**-H**	-	-	-
					**+H**	**Y,O**; I	2	0.0004
**4900**	C3ZEP6_BRAFL (aa335-985)	1.8e-57	30.5%	Uncharacterized/similar to calcium-activated chloride channel regulator 2; domains: VWA, DUF1973; Lotgi1|238844 (2.9e-135)	**-H**	**Y,O**; I	3	0.0020
					**+H**	**Y,O,B**; S<I	5	0.0067
**4963**				Uncharacterized; domains: EGF_like, signal peptide	**-H**	**Y,O**; I	7	0.0021
					**+H**	**Y,O**; S<I	14	0.0136
**49657**	K1R8I8_CRAGI (aa365-484)	8.5e-40	75.8%	Uncharacterized; domain: Chloramphenicol acetyltransferase-like; Lotgi1|137484 (2.7e-59)	**-H**	**Y,O,B**; I	1	0.0005
					**+H**	**Y,B**; I	2	0.0015
**49666**				Uncharacterized; 26% T; Lotgi1|169040 (1.1e-12)	**-H**	**Y,O,B**; S	4	0.0095
					**+H**	**Y,O**; S<I	2	0.0053
**49914**	R7V680_CRAGI	5.0e-60	30.6%	Uncharacterized; domains: TSP_1, ADAM, spacer1, PLAC, signal peptide; Lotgi1|237754 (5.2e-163)	**-H**	**Y**; I	1	0.0005
					**+H**	**Y,O**; S<I	9	0.0046
**50**	J9JJV2_ACYPI (aa8-210)	9.7e-28	37.9%	Uncharacterized; domains: Chit_bind_3, signal peptide; Lotgi1|96362 (2.0e-29)	**-H**	**Y,O,B**; S<I	5	0.0141
					**+H**	**Y,O,B**; S,I	6	0.0537
**50027**	Q8ITH0_BIOGL (aa1-164)	1.8e-71	99.4%	α-actinin; Lotgi1|189716 (3.0e-89)	**-H**	**Y,O,B**; I	2	0.0018
					**+H**	**Y,O,B**; S<I	6	0.0124
**50065**				Uncharacterized; domains: signal peptide, RNASE_T2_1; Lotgi1|76871 (1.5e-289)	**-H**	**Y**; I	1	0.0004
					**+H**	**Y,O**; S>I	1	0.0017
**5087**				Uncharacterized; transmembrane; Lotgi1|79004 (2.5e-8)	**-H**	**Y,O,B**; S<I	33	0.5426
					**+H**	**Y,O,B**; S<I	39	1.5962

**5102**	C3YGQ2_BRAFL (aa322-501)	2.5e-14	32.3%	Uncharacterized; domain: ZP_2	**-H**	**Y,B**; I	3	0.0172
					**+H**	**Y,O,B**; I	5	0.0221
**5116**<br>**45664**	I7EDT1_ANTYA<br>R7U3Y2_9ANNE	8.7e-66<br>1.4e-51	93.8%<br>87.1%	ADP-ribosylation factor; Lotgi1|236204 (2.8e-108/2.9e-85); very similar	**-H**	**O**; S	2	0.0007
					**+H**	**B,Y**; I	3	0.0020
**5153**	K1QEW1_CRAGI	2.9e-30	51.7%	Glutathione peroxidase; Lotgi1|86188 (5.0e-45)	**-H**	**Y,O**; I	2	0.0013
					**+H**	**Y,O**; S<I	2	0.0036
**51587**	K1R005_CRAGI (aa34-401)	7.0e-92	55.7%	Filamin-C fragment; Lotgi1|231195 (4.0e-169)	**-H**	**-**	-	-
					**+H**	**Y,O,B**; I	2	0.0001
**51657**				Uncharacterized; signal peptide	**-H**	**Y,O,B**; I	1	0.0468
					**+H**	**Y,O,B**; I	1	0.0152
**5168**	E9GA59_DAPPU	1.9e-123	80.6%	Uncharacterized/eukaryotic initiation factor 4A; domains: (signal peptide,) DNA/RNA helicase; Lotgi1|207101 (0e0)	**-H**	**Y,O,B**; S<I	4	0.0017
					**+H**	**Y,O,B**; I	6	0.0039
**51882**	Q3S3F8_MYTED (aa647-784)	8.8e-20	39.2%	Similar to phospholipase A2; Lotgi1|222658 (4.3e-35)	**-H**	**Y,O,B**; S	1	0.0081
					**+H**	**Y,O,B**; S	1	0.0162
**5201**<br>**5202**	K1PWR0_CRAGI	5.1e-59	78.2%	SET nuclear oncogene; domain: NAP; Lotgi1|143784 86.3e-110)	**-H**	**Y,O,B**; I	3	0.0116
					**+H**	**Y,O,B**; I	1	0.0030
 52099<br>**95524**	K1Q1S3_CRAGI<br>(aa301-952)<br>(aa1-299)	 4.9e-147<br>3.4e-104	 71.9%<br>80.9%	Myosin-VI; Lotgi1|133465 (0e0/9.1e-178)	**-H**	**-**	-	-
					**+H**	**Y**; I<br>**Y,O**; I	7<br>2	0.0016<br>0.0015
**521**	B2YI43_9BILA	1.7e-35	74.2%	Ribosomal protein L24; Lotgi1|106244 81.0e-45)	**-H**	**O,B**; I	1	0.0012
					**+H**	**Y,O,B**; I	1	0.0017
**5270**	K1QVU9_CRAGI (aa58-202)	2.7e-7	37.0%	Uncharacterized; signal peptide	**-H**	**Y,O,B**; S,I	6	0.0319
					**+H**	**Y,O,B**; S,I	6	0.0457
**5306**	RL26_LITLI	1.5e-46	84.6%	60S ribosomal protein L26; Lotgi1|189002 (5.4e-57)	**-H**	**B,Y**; S,I	1	0.0019
					**+H**	**B**; S,I	1	0.0005

**53616**	K1PUR2_CRAGI	8.1e-52	50.6%	Nucleobindin-2; domains: signal peptide, EFh; Lotgi1|233369 (9.5e-98); also see contig_7760!	**-H**	**Y,O**; I	2	0.0007
					**+H**	**Y,O**; S<I	6	0.0015
**5379**	K1PJE5_CRAGI (aa1-261)	8.8e-49	47.7%	Pancreatic triacylglycerol lipase (Fragment); domains: signal peptide, lipase; Lotgi1|216931 (4.5e-80)	**-H**	**Y**; I	3	0.0026
					**+H**	**Y,O**; I	5	0.0093
**5380**	K1PJE5_CRAGI (aa1-272)	4.2e-49	48.6%	Pancreatic triacylglycerol lipase (Fragment); domains: signal peptide, lipase; Lotgi1|216931 (3.6e-78)	**-H**	**Y,O**; I	3	0.0022
					**+H**	**Y,O**; S<I	5	0.0088
**5381**	F0V4D4_MYTGA	5.0e-20	30.3%	Similar to C1q domain containing protein MgC1q97; domains: complement_C1q/TNF_like; Lotg1|138166 (4.8e-14)	**-H**	**Y,O,B**; I	3	0.0007
					**+H**	**O,B**; I	3	0.0048
**53924**	D2A034_TRICA	6.8e-99	45.7%	Uncharacterized; domains: signal peptide, sulfatase/alkaline_phosphatase; Lotgi1|124040 (0e0)	**-H**	-	-	-
					**+H**	**Y,O**; I	2	<0.0001
**5404**	B8XY08_OCHTA	1.1e-13	57.6%	ATP synthase-coupling factor 6, mitochondrial; Lotgi1|236637 (7.5e-20)	**-H**	**Y,O,B**; I	3	0.0031
					**+H**	-	-	-
**5474**	Q6WMS9_BRABE	1.3e-59	53.9%	Interferon gamma-inducible protein 30; domains: signal peptide, saposin_A, GILT; Lotgi1|235566 (1.0e-91)	**-H**	**Y,O**; S	1	0.0021
					**+H**	**Y,O,B**; S	2	0.0024
**54938**	R7U8T0_9ANNE (aa1585-2150)	2.2e-38	26.4%	Uncharacterized/similar to cadherin-23 (K1PXB6_CRAGI; aa1252-1831); Lotgi1|229248 81.8e-34)	**-H**	**Y,O**; I	1	0.0003
					**+H**	**Y,O**; I	6	0.0038
**551**				Uncharacterized; fragment of 67aa containing 18% G, 22% M, 12% P	**-H**	**Y,O,B**; S>I	3	1.0562
					**+H**	**Y,O,B**; S>I	3	0.8513
**55282**	K1RCC7_CRAGI (aa22-234)	4.9e-21	33.5%	Similar to delta-like protein; domains: signal peptide, EGF, DSL; Lotgi1|169085 (2.0e-19)	**-H**	**Y,B**; I	2	0.0005
					**+H**	**Y,O**; S,I	3	0.0056
**553**	A5GZU9_APLCA (aa54-172)	5.0e-40	83.2%	Ferritin; Lotgi1|183389 (2.8e-57)	**-H**	**Y,O**; I	3	0.0014
					**+H**	**Y,O**; S,I	3	0.0026
**55330**				Uncharacterized; 11% R	**-H**	**Y,O,B**; S>I	1	0.0064
					**+H**	**Y,O,B**; S>I	2	0.0118

**5682**				Uncharacterized	**-H**	**Y,O,B**; S<I	3	0.0256
					**+H**	**Y,O**; I	1	0.0026
**57**	TPM_HELLAS	1.1e-55	95.1%	Tropomyosin; shares peptides with contig_6620; Lotgi1|162585 (8.7e-106)	**-H**	**Y,O,B**; I	8	0.0055
					**+H**	**Y**; I	1	0.0018
**5701**	H9GFP3_ANOCA (aa244-360)	3.9e-37	71.8%	Uncharacterized/V-type proton ATPase subunit d 1; Lotgi1|108853 (3.7e-61)	**-H**	**Y,O**; I	5	0.0100
					**+H**	**Y,O**; S<I	8	0.0638
**57047**	G4TBF6_PIRID	4.5e-71	49.6%	Similar to actin; Lotgi1|232973 (2.7e-99)	**-H**	**Y,O,B**; I	2	0.0016
					**+H**	**Y,O,B**; S<I	13	0.0129
**57198**	C3ZGK4_BRAFL (aa8-442)	1.1e-116	63.8%	Uncharacterized; domain: ALDH_like; Lotgi1|207312 80e0)	**-H**	**Y,O,B**; I	5	0.0021
					**+H**	**Y,O**; S<I	12	0.0158
**572**	IFEA_HELAS	1.7e-118	96.9%	Non-neuronal cytoplasmic intermediate filament protein; Lotgi1|109284 86.9e-119)	**-H**	**Y,O,B**; S>I	27	0.1623
					**+H**	**Y,O,B**; S<I	32	0.1862
**57844**	E9GB01_DAPPU	5.3e-65	55.4%	aa97-572 similar to adenylyl cyclase-associated protein; Lotgi1|92365 (1.2e-130)	**-H**	**-**	-	-
					**+H**	**Y**; I	2	0.0003
**58150**	K1QZ49_CRAGI (aa85-348)	1.2e-40	35.1%	Similar to adipocyte plasma membrane-associated protein; domains: signal peptide, TolB_like/ strictosidine synthase; Lotgi1|115637 (5.6e-40); see also contig_221 and contig_16710	**-H**	**Y,O,B**; S>I	15	0.7376
					**+H**	**Y,O,B**; S<I	16	1.9756
**5882**	K1RAL0_CRAGI	4.6e-105	58.0%	Aspartate aminotransferase, cytoplasmic; Lotgi1|231665 (5.4e-171)	**-H**	**Y,O**; I	2	0.0007
					**+H**	**Y,O**; S<I	11	0.0056
**5888**	C3Z7R8_BRAFL	2.6e-55	36.8%	Uncharacterized; domain: peptidase_M16; Lotgi1|156673 (1.4e-49)	**-H**	**Y,O,B**; I	7	0.0035
					**+H**	**Y,O,B**; I	9	0.0053
**59326**	F4WIU3_ACREC	2.4e-50	67.3%	Peptidyl-prolyl cis-trans isomerase; Lotgi1|212754 (3.2e-74)	**-H**	**Y,O,B**; I	2	0.0049
					**+H**	**Y,O,B**; S<I	4	0.0155
**5939**	ASSY_DANRE	1.0e-113	69.0%	Argininosuccinate synthase; Lotgi1|231283 (0e0)	**-H**	**-**	-	-
					**+H**	**Y**; I	2	0.0005

**5979**	K1RZJ5_CRAGI (aa234-712)	3.3e-28	24.8%	Similar to Alba (D-arabinono-1,4-lactone oxidase)-like protein C9orf23-like protein; domains: signal peptide, FAD_binding, ALO; Lotgi1|159383 (0e0)	**-H**	**Y,O,B**; I	9	0.0045
					**+H**	**Y,O,B**; I	3	0.0018
**5989**	K1PPZL2_CRAGI (aa923-1649)	1.2e-204	57.0%	Maltase-glucoamylase, intestinal; domains: glycoside_hydrolase_31, transmembrane; Lotgi1|102400 (0e0)	**-H**	**Y,O**; S<I	5	0.0011
					**+H**	**Y,O,B**; S<I	5	0.0032
**60305**	B7Q2C4_IXOSC	6.8e-14	37.5%	Similar to alpha-B-crystallin; see also contig_6923; Lotgi1|104895 (1.6e-19)	**-H**	**Y,O,B**; I	1	0.0008
					**+H**	**Y,B**; I	2	0.0019
**60675**				Uncharacterized; domain: CLECT; Lotgi1|164559 (2.9e-60)	**-H**	**Y,O**; I	2	0.0007
					**+H**	**Y,O**; S,I	3	0.0031
**60916**	R7VDB2_9ANNE (aa53-252)	3.6e-12	32.2%	Uncharacterized; domains: CMB_14/ CHIT_BIND_II; Lotgi1|157323 (6.0e-38)	**-H**	**Y,O**; S,I	2	0.0067
					**+H**	**Y,O**; S,I	4	0.0116
**6176**				Uncharacterized; 11% A, 11% G, 12% M	**-H**	**Y,O,B**; S>I	6	0.4285
					**+H**	**Y,O,B**; S>I	7	0.4872
**61949**	D2JPI4_HALDH	7.4-162	66.5%	Selenium-binding protein; Lotgi1|203681 (0e0)	**-H**	**Y,O**; I	1	0.0001
					**+H**	**Y,O**; I	7	0.0040
**6246**	K1R2H1_CRAGI (aa341-659)	1.1e-21	37.8%	Putative tyrosinase-like protein tyr-3; Lotgi1|166196 (1.2e-71)	**-H**	**Y,B**; I	1	0.0078
					**+H**	**Y,O,B**; S<I	3	0.0106
**62484**	L7M2C1_9ACAR	1.4e-79	77.0%	Similar to prohibitin; Lotgi1|237446 (7.5e-122)	**-H**	**-**	-	-
					**+H**	**O**; I	2	0.0009
	D7R6D6_BIOGL			Thioester-containing protein 1.4; domains: A2M; Lotgi1|162872 (6.4e-87/1.3e-45)				
**6275**	(aa728-1100)	6.2e-71	49.7%		**-H**	**Y,O,B**; S<I	7	0.0357
**660**	(aa1191-1445)	3.5e-59	51.2%			**Y,O,B**; S<I	9	0.0181
					**+H**	**Y,O,B**; S<I	6	0.0172
						**Y,O**; S<I	5	0.0063
**6295**				Uncharacterized	**-H**	**Y,O,B**; I	4	0.0072
					**+H**	**B**; I	1	0.0010

**63304**	K1Q365_CRAGI (aa22-658)	9.6e-55	29.8%	aa127-818 similar to lactadherin; domains: CBM_14/CHIT_BIND_II; Lotgi1|231869 (2.7e-110)	**-H**	**Y,O,B**; S<I	17	0.0373
					**+H**	**Y,O,B**; S<I	26	0.2100
**63401**	K1QNZ0_CRAGI (aa2-167)	7.2e-19	34.9%	Uncharacterized; signal peptide; Lotgi1|160424 (4.4e-31)	**-H**	**-**	-	-
					**+H**	**Y,O**; S<I	4	0.0042
**63801**	D6X1V1_TRICA (aa43-1636)	0e0	60.6%	Uncharacterized/similar to chromodomain-helicase-DNA-binding protein Mi-2-like protein; Lotgi1|165279 (0e0)	**-H**	**Y,O**; S,I	2	<0.0001
					**+H**	-	-	
**64075**	H3IE31_STRPU (aa101-280)	1.6e-29	42.0%	Deoxyribonuclease; Lotgi|168246 (1.0e-36)	**-H**	**Y,O**; I	3	0.0049
					**+H**	**Y,O**; S<I	5	0.0247
**6453**	A5HJT4_BIOGL (aa142-289)	1.2e-22	45.8%	Tandem-repeat galectin; Lotgi1|129554 (4.6e-22)	**-H**	**Y,O,B**; S<I	3	0.0089
					**+H**	**Y,O,B**; S<I	4	0.0125
**6457**	PGAM_DICDI (aa91-249)	3.7e-47	68.6%	Phosphoglycerate mutase; Lotgi1|187919 (1.5e-69)	**-H**	**-**	-	-
					**+H**	**Y,O**; I	3	0.0054
**64786**	A7RQD5_NEMVE (aa109-331)	9.0e-17	30.1%	Uncharacterized	**-H**	**O,B**; I	1	0.0014
					**+H**	**Y,O**; I	3	0.0046
**6530**	K1QYG7_CRAGI	1.9e-193	68.8%	Glucosamine-fructose-6-phosphate aminotransferase [isomerizing] 1; Lotgi1|235105 (0e0)	**-H**	**Y,O**; I	2	0.0002
					**+H**	**Y,O**; I	12	0.0034
**6553**	C1BEZ2_ONCMY	1.1e-52	100%	Histone H3; Lotgi1|150994 (2.0e-86)	**-H**	**Y,O,B**; S<I	2	0.0318
					**+H**	**Y,O,B**; I	1	0.0206
**65969**	L8IY93_BOSMU (aa64-1518)	4.9e-49	25.5%	Similar to SCO-spondin; domains: signal peptide, EGF, VWD, LDLR, Trypsin_inhibitor_like; Lotgi1|174711 (1.2e-78)	**-H**	**Y,O,B**; S<I	16	0.0083
					**+H**	-	-	-
**6609**				Uncharacterized	**-H**	**-**	-	-
					**+H**	**Y**; I	3	0.0023
**6620**	TPM1_BIOGL	3.6e-47	78.4%	Tropomyosin-1; Lotgi1|233095 (4.6e-100); shares peptides with contig_57	**-H**	**Y,O**; I	4	0.0034
					**+H**	**Y,O,B**; I	3	0.0013

**66355**	B6DVV3_APLCA	2.4e-98	75.2%	Src tyrosine kinase 1; Lotgi1|224844 (0e0)	**-H**	-	-	-
					**+H**	**Y**; I	3	0.0005
**6654**	C7FPZ1_9TELE	1.6e-52	51.7%	Carbonic anhydrase; domain: α-CA; Lotgi1|205401 81.9e-93)	**-H**	**Y,B**; I	2	0.0008
					**+H**	**Y,O**; I	4	0.0058
**6677**<br>**14914**	Q75R52_LYMST<br>(aa428-539)<br>(aa117-421)	1.0e-12<br>2.2e-18	40.2%<br>31.9%	Similar to DEC-1; domain: VWA; Lotgi1|234764 (7.9e11)	**-H**	**Y,B**; I	3	0.0021
					**+H**	**Y,O,B**; I	4	0.0068
						**O**; I	1	0.0019
**66822**				Uncharacterized; 13% P, 25% T	**-H**	**Y,O,B**; S<I	3	0.0117
					**+H**	**Y,O,B**; S,I	3	0.0210
**6695**	M4A0J3_XIPMA	8.5e-153	85.3%	Uncharacterized/Arp3; Lotgi1|193923 (0e0)	**-H**	-	-	-
					**+H**	**Y,O**; I	4	0.0011
**6697**	K1QZ49_CRAGI<br>(aa176-409)	1.3e-29	37.9%	Similar to adipocyte plasma membrane-associated protein; shares peptide with 8812; domains: signal peptide, TolB_like/strictosidine_synthase_related; See also contig_16710 and 221; Lotgi1|115667 (1.3e-37)	**-H**	**Y,O**; I	4	0.0028
					**+H**	**Y,O,B**; S<I	8	0.0303
**67**	G3FPE5_HELLU<br>(aa2976-3299)	1.0e-100	66.7%	Hemocyanin (β?); shares peptide with contig_297; Lotgi1|234481 (7.1e-7)	**-H**	**Y,O,B**; S<I	5	0.0252
					**+H**	**Y,O,B**; I	7	0.0164
**671**	E0D8P7_GRYBI<br>(aa1-214)	5.5e-49	88.4%	Elongation factor 1-alpha; Lotgi1|239271 (4.6e-121)	**-H**	**Y,O,B**; S<I	5	0.0433
					**+H**	**Y,O,B**; S<I	4	0.0627
**67057**	L7M2D5_9ACAR	9.2e-12	45.2%	Similar to Vacuolar ATP synthase subunit G; Lotgi1|220690 (1.6e-27)	**-H**	-	-	-
					**+H**	**Y**; S<I	2	0.0010
**67514**<br>**33100**	K1QE94-CRAGI<br>(aa272-397)<br>(aa4-209)	4.3e-20<br>6.5e-64	46.8%<br>66.2%	α-N-acetylgalactosaminidase; Lotgi1|178842 (9.0e-25)/209340 (7.2e-100)	**-H**	**Y,O**; I	2	0.0052
					**+H**	**Y,O**; S<I	2	0.0205
						**Y,O**; I	3	0.0007

**6756**	R7V680_9ANNE (166-607)	9.4e-51	38.8%	Aa1041-1494 similar to uncharacterized; domains: signal peptide, TSP_2, ADAM_spacer1, PLAC, peptidase_M12B; Lotgi1|51613 (2.2e-146)	**-H**	-	-	-
					**+H**	**O**; S,I	2	0.0004
**6809**	Q8ITA1_ARGIR	7.4e-52	76.4%	Ribosomal protein L12; Lotgi1|217320 (1.6e-86)	**-H**	-	-	-
					**+H**	**Y,B**; I	2	0.0021
**6813**				Uncharacterized; 16% S, 17% T	**-H**	**Y,O,B**; S>I	7	0.0292
					**+H**	**Y,O,B**; S>I	6	0.0141
**6854**	E3WTS1_ANODA	3.0e-94	68.5%	Malate dehydrogenase; Lotgi1|188534 (7.4e-132)	**-H**	**Y,O**; I	3	0.0014
					**+H**	**Y,O**; I	4	0.0129
**68877**	K1R4D4_CRAGI	1.3e-84	70.5%	40S ribosomal protein SA; Lotgi1|203487 (7.0e-111)	**-H**	**Y,O,B**; S<I	3	0.0053
					**+H**	**Y,O,B**; I	2	0.0045
**68904**	R4ZGP6_9ACAR (aa282-601)	3.9e-29	28.4%	Similar to acetylcholinesterase; Lotgi1|113809 (1.3e-26)	**-H**	**Y,O,B**; I	6	0.0101
					**+H**	-	-	-
**69084**	D2A249_TRICA	9.3e-132	53.8%	Uncharacterized; domains: ricin_B_lectin, glyco_tranf_2, glycol_transf_7C; Lotgi1|194702 (0e0)	**-H**	**Y**; I	2	<0.0001
					**+H**	**Y,O**; I	8	0.0019
**691**	A5Z1D6_9EUPU	1.4e-89	43.3%	Similar to epiphragmin; domains: signal peptide, Fibr_C; Lotgi1|83908 (1.7e-27)	**-H**	**Y,O,B**; S<I	32	0.3074
					**+H**	**Y,O,B**; S<I	31	0.2048
**69179**	K1R1Q8_CRAGI	1.4e-64	88.3%	Ras-related protein Rab-5C; Lotgi1|222012 (4.9e-119)	**-H**	-	-	-
					**+H**	**Y,B**; I	4	0.0016
**6923**	B7Q2C4_IXOSC	1.8e-14	41.3%	Similar to α-B-crystallin; see also contig_60305; Lotgi1|104895 (8.9e-21)	**-H**	**Y**; I	2	0.0011
					**+H**	**Y**; I	2	0.0020
**695**	C1KC83_HALDV	0e0	89.7%	Heat shock cognate protein 70; Lotgi1|177837 (0e0)	**-H**	**Y,O,B**; S<I	17	0.0213
					**+H**	**Y,O,B**; S<I	21	0.0463
**6969**	K1RW85_CRAGI	5.8e-159	83.0%	Adenosylhomocysteinase; Lotgi1|184532 (0e0)	**-H**	**Y,O,B**; S<I	1	0.0016
					**+H**	**Y,O,B**; I	2	0.0041

**70388**	Q45R39_APLCA (aa387-586)	1.1e-35	71.0%	Ezrin/radixin/moesin; Lotgi1|59617 (9.3e-109)/106937 (0e0)	**-H**	**Y,O**; I	2	0.0006
**873**	(aa1-586)	4.2e-108	78.7%	Very similar sequences sharing many peptides!		**Y,O,B**; I	23	0.0146
**16517**	(aa1-393)	6.2e-113	92.6%			**-**	-	-
					**+H**	**Y,O**; I	3	0.0050
						**Y,O,B**; S<I	32	0.0645
						**Y,O,B**; I	7	0.0047
**7072**	Q2VU37_PINFU (aa23-375)	5.1e-32	32.1%	Similar to astacin-like protein; domains: signal peptide, peptidase_M12A; Lotgi1|109273 (2.9e-37)	**-H**	**Y,O**; I	4	0.0010
					**+H**	**Y,O**; S<I	10	0.0051
**7075**	R7V800_9ANNE	9.9e-42	37.7%	Uncharacterized/similar to lachesin; domains: transmembrane, IG; Lotgi1|234163 (5.5e-88)	**-H**	**Y,O**; I	2	0.0006
					**+H**	**Y,O**; S<I	5	0.0037
**71**	C3ZKR4_BRAFL (aa193-354)	5.8e-15	38.3%	aa6-167 similar to adipocyte plasma membrane-associated protein (K1QZ49_CRAGI); domain: TolB_like/strictosidine_synthase_related; Lotgi1|115667 (3.1e-26)	**-H**	**Y,O,B**; S<I	8	0.0310
					**+H**	**Y,O,B**; S<I	9	0.0494
**7158**	Q45R40_APLCA (aa181-1225)	0e0	84.3%	Nonmuscle myosin II (Fragment); domains: IQ_motif, myosin_head, myosin_tail_1; Lotgi1|205893 (0e0)	**-H**	**Y,O,B**; S<I	27	0.0029
					**+H**	**Y,O,B**; S<I	48	0.0121
**71616**	R7UUZ8_9ANNE	0e0	61.9%	aa121-979 similar to Uncharacterized/puromycin-sensitive aminopeptidase; domain: peptidase_M1; Lotgi1|204438 (0e0)	**-H**	**Y,O**; I	4	0.0001
					**+H**	**Y,O**; I	13	0.0016
**72045**	K1QDH9_CRAGI (aa1-216)	1.2e-77	83.9%	Myosin-11; Lotgi1|177615 (8.6e-122)	**-H**	**O**; S,I	3	0.0006
					**+H**	**Y,O,B**; I	6	0.0060
**72620**	F8RP10_CRAGI (aa12-419)	5.5e-39	30.0%	Similar to Bactericidal permeability increasing protein; domains: BPI, signal peptide; Lotgi231708 (8.7e-52)	**-H**	**Y,O,B**; I	1	0.0021
					**+H**	**Y,O**; S<I	2	0.0043
**7323**	A7RQD5_NEMVE	6.2e-41	32.2%	Uncharacterized; signal peptide	**-H**	**Y,O,B**; S<I	16	0.0768
					**+H**	**Y,O,B**; S<I	25	0.1902
**7335**	F6VEH7_XENTR (aa475-4265)	0e0	46.0%	Uncharacterized/similar to Low-density lipoprotein receptor-related protein; domains: LDLR, EGF, TolB_like; Lotgi1|170222 (0e0)	**-H**	**Y,O,B**; I	41	0.0044
					**+H**	**Y,O,B**; S<I	88	0.0246

**7341**				Uncharacterized; very G-rich N-terminus (aa1-80 61% G)	**-H**	**Y,O,B**; S<I	4	0.0269
					**+H**	**Y,O,B**; S<I	4	0.0240
**7386**	K1Q7T5_CRAGI	3.5e-115	58.2%	Protein disulfide-isomerase A3; signal peptide; Lotgi1|190816 (3.1e-170)	**-H**	**Y,O,B**; I	6	0.0022
					**+H**	**Y,O,B**; I	8	0.0026
**74050**	E9HK57_DAPPU	4.1e-67	58.4%	Uncharacterized/calumenin-B; domains: signal peptide, EFh; Lotgi1|103470 (2.3e-125)	**-H**	**-**	-	-
					**+H**	**Y**; I	2	0.0005
**74063**				Uncharacterized; signal peptide; 10% G, 13% L, 20% P; only 1 peptide possible!	**-H**	**Y,O,B**; S>I	1	0.2061
					**+H**	**Y,O,B**; S,I	1	0.2185
**7416**	B0L0Y3_PINFU (aa13-540)	5.5e-116	66.4%	RNA-binding protein; Lotgi1|93013 (0e0)	**-H**	**-**	-	-
					**+H**	**Y**; I	2	0.0002
**74191**	K1PLV6_CRAGI	5.2e-91	71.0%	F-actin-capping protein subunit alpha; Lotgi1|196809 (7.2e-149)	**-H**	**-**	-	-
					**+H**	**Y**; I	6	0.0031
**7508**				Uncharacterized; signal peptide; Lotgi1|234386 (1.4e-31)	**-H**	**Y,O,B**; S<I	13	2.7736
					**+H**	**Y,O,B**; S<I	15	6.4446
**7563**				Uncharacterized	**-H**	**Y,O,B**; S<I	6	0.1193
					**+H**	**Y,O,B**; S,I	6	0.5070
**75801**	H9K6W2_APIME (aa1146-1454)	7.9e-21	34.9%	Uncharacterized; domains: cadherin; Lotgi1|167053 (1.44e-21); also see contig_20360	**-H**	**Y,O,B**; I	6	0.0222
					**+H**	**Y,O,B**; S,I	7	0.0732
**7594**	D8V7I9_BIOGL (aa233-352)	6.6e-46	91.7%	Arginine kinase; Lotgi1|183052 (9.4e-65)	**-H**	**Y,B**; I	4	0.0066
					**+H**	-	-	
**76025**	K1QMK1_CRAGI	1.2e-39	48.6%	Uncharacterized; domain: aerolysin_like_toxin; Lotgi1|152519 (3.1e-51)	**-H**	**Y,O,B**; I	1	0.0005
					**+H**	**Y,O,B**; I	1	0.0010
**763**	Q95PK2_BIOGL (aa467-667)	3.6e-35	43.7%	BgMFREP7 (Fragment)/fibrinogen-related protein 7; domain: fibrinogen_C; Lotgi1|83908 (1.7e-37)	**-H**	**O,B**; I	3	0.0007
					**+H**	**O,B**; S<I	7	0.0501

**76308**	K1Q329_CRAGI	7.6e-117	76.8%	Pyruvate dehydrogenase E1 component subunit beta, mitochondrial; Lotgi1|226825 (3.8e-180)	**-H**	**-**	-	-
					**+H**	**Y**; I	3	0.0005
**7641**	E9BZF3_CAPO3	1.3e-54	61.9%	Tyrosine 3-monooxygenase/tryptophan 5-monooxygenase activation protein; domain: 14-3-3; Lotgi1|222442 (6.2e-84)	**-H**	**-**	-	-
					**+H**	**Y,O,B**; S<I	5	0.0020
**76888**	J7HZQ3_POMCA (aa323-528)	4.6e-55	65.0%	Apoptosis-inducing factor 3-like protein; Lotgi1|214061 (5.1e-87)	**-H**	**-**	-	-
					**+H**	**Y**; I	2	0.0021
**77520**	K1QWM6_CRAGI (aa106-326)	5.0e-12	29.5%	Similar to Neural-cadherin; domains: cadherin; Lotgi1|167053 (1.9e-16)	**-H**	**Y,O**; S<I	2	0.0142
					**+H**	**Y,O,B**; S<I	5	0.0867
**7760**	K1PUR2_CRAGI (aa6-387)	1.3e-39	48.2%	Nucleobindin-2; domains: transmembrane, EFh; Lotgi1|163284 (2.9e-81); see also contig_53616!	**-H**	**Y,O**; I	2	0.0006
					**+H**	**Y,O**; S<I	9	0.0050
**77827**	A7RKK8_NEMVE (aa31-190)	2.2e-16	37.6%	Uncharacterized/similar to chitinase; domain: polysaccharide deacetylase/chitinase; Lotgi1|153749 (1.9e-19)	**-H**	**Y,O,B**; I	4	0.0060
					**+H**	**Y,O,B**; S<I	4	0.0091
**7807**				Uncharacterized; domains: signal peptide, WAP; 12% A, 11% Q; Lotgi1|201804 (2.2e-11)	**-H**	**Y,O,B**; S<I	12	0.0471
					**+H**	**Y,O,B**; S<I	15	0.1202
**7809**				Uncharacterized; 21% D, 16% L	**-H**	**Y,O,B**; S<I	16	0.3191
					**+H**	**Y,O,B**; S<I	17	0.8317
**784**				Uncharacterized; signal peptide	**-H**	**Y,O,B**; S>I	1	0.0534
					**+H**	**Y,O,B**; S>I	1	0.0832
**7857**	K1RNX0_CRAGI	5.4e-34	89.0%	Small nuclear ribonucleoprotein E; Lotgi1|207212 (3.8e-51)	**-H**	**Y,O,B**; I	2	0.0028
					**+H**	-	-	-
**78579**	A7RQD5_NEMVE	4.0e-44	31.3%	Uncharacterized	**-H**	**Y,O,B**; I	8	0.0033
					**+H**	**Y,O,B**; I	15	0.0085
**7877**				Uncharacterized; 17% P, 22% S	**-H**	**Y,O,B**; I	3	0.0043
					**+H**	-	-	-

**79063**	L7M8Q4_9ACAR (aa215-442)	2.4e-52	54.5%	Putative carbon-nitrogen hydrolase; domains: HIT, CN_hydrolase; Lotgi1|116186 (1.0e-44)	**-H**	-	-	-
					**+H**	**Y**; I	3	0.0010
**79562**	H2ZK53_CIOSA (aa250-455)	3.8e-5	28.3%	Uncharacterized; domains: CUB; Lotgi1|173347 (2.3e-7)	**-H**	**Y,O,B**; S<I	3	0.0321
					**+H**	**Y,O,B**; S<I	3	0.0157
**79695**	K1QSK4_CRAGI (730-1035)	6.9e-100	78.8%	Calcium-binding mitochondrial carrier protein; Lotgi1|232539 (1.8e-145)	**-H**	-	-	-
					**+H**	**Y**; I	3	0.0003
**7977**	G2QTA6_THITE	2.6e-44	69.4%	Nucleoside diphosphate kinase; Lotgi1|205662 (3.2e-64)	**-H**	**Y,O,B**; I	4	0.0110
					**+H**	**Y,O,B**; S<I	7	0.0504
**80634**	L7N2K8_XENTR	6.0e-110	53.2%	Uncharacterized/prolylcarboxypeptidase (Angiotensinase C); domains: signal peptide, peptidase_S28; Lotgi1|207842 (2.4e-165)	**-H**	-	-	-
					**+H**	**Y,O**; I	3	0.0022
**80985**	L7M2D2_9ACAR	2.6e-43	54.7%	Rho GDP dissociation inhibitor; Lotgi1|183614 (5.8e-60)	**-H**	-	-	-
					**+H**	**Y,B**; I	2	0.0010
**81046**	T1DEH2_9DIPT	4.7e-103	73.5%	Uncharacterized; domains: armadillo/Mo25_like; Lotgi1|238430 (2.0e-162)	**-H**	-	-	-
					**+H**	**Y,O**; I	2	0.0007
**81408**	K1RGK4_CRAGI	0e0	61.1%	Villin-1; Lotgi1|221805 (0e0)	**-H**	-	-	-
					**+H**	**Y,O**; I	8	0.0039
**81515**	E9GBJ8_DAPPU	1.9e-119	77.6%	Uncharacterized/alcohol dehydrogenase; domains:ADH_ZINC, gelsolin; Lotgi1|228995 (1.0e-121)	**-H**	-	-	-
					**+H**	**Y**; I	2	0.0009
**821**	J7Q5J6_PATVU (aa213-366)	8.7e-11	29.9%	Similar to BSMP; domain: CBM_14/ CHIT_BIND_II; Lotgi1|236718 (9.6e-15)	**-H**	**Y,O,B**; S<I	11	1.1640
					**+H**	**Y,O,B**; S<I	15	2.4533
**8216**	E9H8M4_DAPPU	9.9e-86	80.4%	Uncharacterized; domain: 14-3-3; shares peptides with several other 13-3-3 proteins; Lotgi1|207549 (1.9e-115)	**-H**	**Y,O,B**; S<I	11	0.0377
					**+H**	**Y,O,B**; S<I	15	0.0896
**824**	Q5M8U4_XENTR (aa104-281)	9.9e-51	65.7%	Uncharacterized/S-formylglutathione hydrolase; Lotgi1|120210 (3.6e-84)	**-H**	-	-	-
					**+H**	**Y**; I	2	0.0005

**8282**	K1PHW4_CRAGI	2.7e-19	31.1%	Ganglioside GM2 activator; signal peptide; Lotgi1|154381 (1.4e-19)	**-H**	**Y,O,B**; S<I	3	0.0100
					**+H**	**Y,O,B**; S<I	3	0.0179
**83512**	Q4RR82_TETNG	3.0e-16	52.8%	SH3 domain-binding glutamic acid-rich-like protein; 11% D in 92aa; Lotgi1|236598 (4.6e-11)	**-H**	**Y,O,B**; S<I	3	0.0274
					**+H**	**Y,O,B**; S<I	2	0.0336
**8371**	K1QXR1_CRAGI (aa223-422)	7.2e-29	40.9%	Putative tyrosinase-like protein tyr-3; Lotgi1|166196 (7.7e-44); see also contig_4409!	**-H**	**Y,B**; S<I	1	0.0184
					**+H**	**Y,O,B**; S,I	1	0.0216
**84589**	K1QIK2_CRAGI (aa1112-1793)	5.4e-41	29.2%	Uncharacterized; domains: CBM_14/ CHIT_BIND_II; Lotgi1|231869 (1.1e-106); also see contig_101824!	**-H**	**Y,O,B**; S<I	19	0.0627
					**+H**	**Y,O,B**; S<I	29	0.3022
**85080**	S4SBL5_CRAGI (aa1-286)	8.3e-71	55.7%	Acid ceramidase; domains: signal peptide, choloylglycine _hydrolase; Lotgi1|142206 (8.8e-112)	**-H**	**-**	-	-
					**+H**	**Y,O**; I	4	0.0017
**85288**	D2WPC5_HELAM (aa220-344)	6.1e-13	32.3%	Similar to Chitin deacetylase 5a; Lotgi1|116769 (5.0e-10)	**-H**	**Y,O,B**; S<I	3	0.0082
					**+H**	**Y,O,B**; I	2	0.0080
**85737**				Uncharacterized; 19% R, 21% E	**-H**	**-**	-	-
					**+H**	**Y,O**; I	2	0.0022
**86340**				Uncharacterized; signal peptide; 13% R, 18% Q	**-H**	**Y,O,B**; S<I	8	0.0931
					**+H**	**Y,O,B**; S<I	9	0.0784
**86405**	K1RAG0_CRAGI	2.7e-63	51.2%	Glyoxylate reductase/hydroxypyruvate reductase; Lotgi1234402 (7.1e-114)	**-H**	**-**	-	-
					**+H**	**Y**; I	2	0.0004
**8812**	K1QZ49_CRAGI (aa67-398)	7.3e-22	34.3%	Similar to adipocyte plasma membrane-associated protein; domains: signal peptide, TolB_like/ strictosidine synthase; Lotgi1|115637 (1.5e-52); see also contig_221/contig_58150/contig_16710!	**-H**	**Y,O,B**; S<I	24	0.0188
					**+H**	**Y,O,B**; S<I	33	0.0508
**8897**	K1QN14_CRAGI (aa164-315)	5.7e-10	27.4%	Uncharacterized; Lotgi1|228701 (2.0e-23)	**-H**	**Y,O,B**; S<I	6	1.3649
					**+H**	**Y,O,B**; S>I	6	0.0415

**9053**	K1QMD1_CRAGI (aa657-810)	4.7e-16	34.0%	Similar to Putative tyrosinase-like protein tyr-3; domain: CBM_14; Lotgi1|234880 (1.2e-13)	**-H**	**Y,O,B**; S<I	1	0.0106
					**+H**	**Y,O,B**; I	1	0.0021
**9056**	K1QMD1_CRAGI (aa657-818)	9.2e-16	33.7%	Similar to Putative tyrosinase-like protein tyr-3; domain: CBM_14; Lotgi1|234880 (2.1e-19)	**-H**	**-**	-	-
					**+H**	**O**; I	2	0.0046
**91173**	K1QT97_CRAGI	9.6e-47	40.7%	Similar to N(G),N(G)-dimethylarginine dimethylaminohydrolase 1; domain: amidinotransferase; Lotgi1|170725 (3.7e-85)	**-H**	**Y,O,B**; I	2	0.0009
					**+H**	**Y,O,B**; I	3	0.0012
**91443**	K1Q8Q1_CRAGI (aa100-716)	1.1e-133	47.8%	Similar to Laccase-2; domains: signal peptide, cupredoxin; Lotgi1|218365 (7.6e-170)	**-H**	**-**	-	-
					**+H**	**Y,O**; I	2	0.0004
**9158**				Uncharacterized; 14% A, 16% E, 17% G, 17% P; only 1 peptide originating from K/P cleavage; a second one (not found) would be possible with two K/P cleavages (less likely). Almost identical to 9159!	**-H**	**Y,O,B**; S>I	1	0.1360
					**+H**	**Y,O,B**; S<I	1	0.0311
**9159**				Uncharacterized; 13% A, 15% E, 17% G, 16% P, 10% S; only 1 peptide originating from K/P cleavage; a second one (not found) would be possible with two K/P cleavages (less likely). Almost identical to 9158!	**-H**	**Y,O**; S	1	0.0368
					**+H**	**Y,O**; S,I	1	0.0401
**92424**	H3DSC4_PRIPA (aa16-562)	2.0e-19	25.3%	Uncharacterized/similar to zonadhesin; domains: EGF, VWD, TIL, CLECT, mucin2_WxxW; Lotgi1|189815 (4.4e-18)	**-H**	**Y,O,B**; I	16	0.0029
					**+H**	**O**; I	6	0.0004
**9283**	Q38RA2_APLKU	1.7e-20	44.2%	Cofilin	**-H**	**-**	-	-
					**+H**	**Y,O**; S<I	4	0.0071
**93705**	K1QRH0_CRAGI (aa306-545)	4.8e-72	63.1%	Lactadherin; Lotgi1|152889 (1.3e-119)	**-H**	**-**	-	-
					**+H**	**Y,O**; I	3	0.0024
**93725**	K1RJA2_CRAGI	1.1e-32	35.0%	Uncharacterized; domains: transmembrane, tetraspanin; Lotgi1|157852 (2.6e-23)	**-H**	**-**	-	-
					**+H**	**Y,O**; I	3	0.0028
**9415**	K1QQS0_CRAGI (aa152-382)	8.5e-13	26.9%	Uncharacterized protein; shares peptide with 7323	**-H**	**Y,O**; S	4	0.0020
					**+H**	**Y,O**; S<I	7	0.0241

**943**	G0ZGZ8_9ANNE (aa98-189)	5.2e-38	97.8%	Actin; shares peptides with CON; only Lottia-specific peptides were considered; Lotgi1|227913 (1.2e-56)	**-H**	**Y,O,B**; S<I	3	0.0621
					**+H**	**Y,O,B**; S<I	3	0.1637
**94717**	K1P7W2_CRAGI (aa256-648)	4.7e-25	33.3%	Similar to Protocadherin-like wing polarity protein stan; domains: cadherin; Lotgi1154324 (4.0e-41); aa2-237 similar to cadherin-89D/protocadherin Fat 4	**-H**	**Y,O,B**; I	3	0.0017
					**+H**	**Y,O,B**; I	3	0.0030
**9513**	E7EZG1_DANRE (aa1219-2186)	6.6e-28	24.5%	Aa285-1251 similar to Uncharacterized; domains: signal peptide, CLECT (aa49-133); rest T- and S-rich; Lotgi1|148957 (6.2e-24)	**-H**	**Y,O,B**; S>I	2	0.0015
					**+H**	**Y,O,B**; S>I	3	0.0011
**96261**	A7S6G6_NEMVE	2.1e-111	57.8%	Uncharacterized/plasma glutamate carboxypeptidase; domains: signal peptide, peptidase_M28; Lotgi1|227502 (1.9e-170)	**-H**	**-**	-	-
					**+H**	**O**; I	6	0.0012
**96767**	S4RPI9_PETMA	5.0e-64	69.9%	Uncharacterized/V-type proton ATPase subunit D; Lotgi1|233963 (4.5e-91)	**-H**	**Y,O**; I	3	0.0019
					**+H**	**Y,O**; S<I	7	0.0238
**97036**	R7U2H6_9ANNE (aa162-519)	2.1e-93	59.2%	Uncharacterized/similar to galactocerebrosidase; domains: Glyco_hydro_59; Lotgi1|218637 (2.0e-149)	**-H**	**-**	-	-
					**+H**	**Y,O**; I	2	0.0029
**98**	K1PV35_CRAGI (aa1-689)	2.9e-178	64.3%	Kyphoscoliosis peptidase; domains: transglutaminase_like/nebulin/Cys_proteinases; Lotgi1218011 (0e0)	**-H**	**-**	-	-
					**+H**	**Y**; I	5	0.0003
**9860**	G7YU18_CLOSI (aa97-716)	2.6e-80	36.6%	Similar to calpain-B; domains: peptidase_C2, EFh; Lotgi1|184301 (7.0e-77)	**-H**	**-**	-	-
					**+H**	**Y**; I	4	0.0011
**9876**	E3WUN5_ANODA	3.5e-38	65.3%	Uncharacterized/mitochondrial cytochrome c oxidase subunit Va; Lotgi1|157909 (5.3e-25)	**-H**	**Y,O**; I	1	0.0011
					**+H**	**Y**; I	1	0.0009
**9926**	R7VB66_9ANNE (57-324)	1.2e-57	49.6%	Uncharacterized; domain: metridin_like_Shk_toxin; Lotgi1|235549 (1.1e-6); also see contig_65431	**-H**	**-**	-	-
					**+H**	**Y,O**; S<I	2	0.0015
**99566**	K1RA95_CRAGI (aa320-837)	8.2e-144	63.5%	aa136-667 similar to filamin-A; domains: filamin/IG; Lotgi1|231196 (0e0)	**-H**	**-**	-	-
					**+H**	**Y**; I	3	0.0005
**99847**	HSP74_MOUSE (aa1-670)	4.6e-123	56.5%	Similar to Heat shock 70 kDa protein 4; domains: HSP70; Lotgi1|111562 (0e0)	**-H**	**-**	-	-
					**+H**	**Y**; I	11	0.0017

**99885**	Q28X57_DROPS (aa131-396)	4.0e-45	41.9%	aa20-287similar to β1,4-N-acetylglucosaminyltransferase; Lotgi1|229370 (4.6e-50)	**-H**	**-**	-	-
					**+H**	**Y,O**; S,I	2	0.0012

Y, yellow shells; **O**, orange shells; **B**, brown (on yellow ground). **S**, acid-soluble matrix; **I**, acid-insoluble matrix. **H**, hypochlorite treatment. The table contains identifications with $\geq$ 2 sequence-unique peptides identified at least three times in total and identifications with only one sequence-unique peptide occurring in at least 3 different replicates and in +H and –H samples. All identified proteins including the ones not accepted are given in Additional files nn.
