# Supplementary figures and images for: Characterization of the pigmented shell-forming proteome of the common grove snail Cepaea nemoralis
Source: BMC Genomics. 2014 Mar 31;15:249. doi: 10.1186/1471-2164-15-249 (PMC4023409; doi:10.1186/1471-2164-15-249)

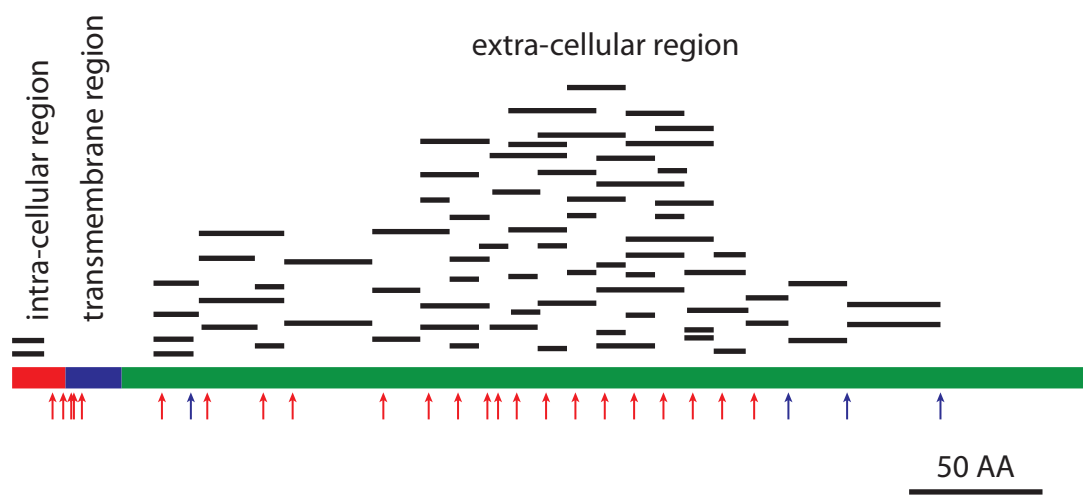

Supplement: Additional file 6 — A schematic representation of a C. nemoralis putative trans-membrane protein (derived from isotig_5087), onto which the spatial distribution of the 72 LC-MS peptides are mapped. [file 1471-2164-15-249-S6.pdf]

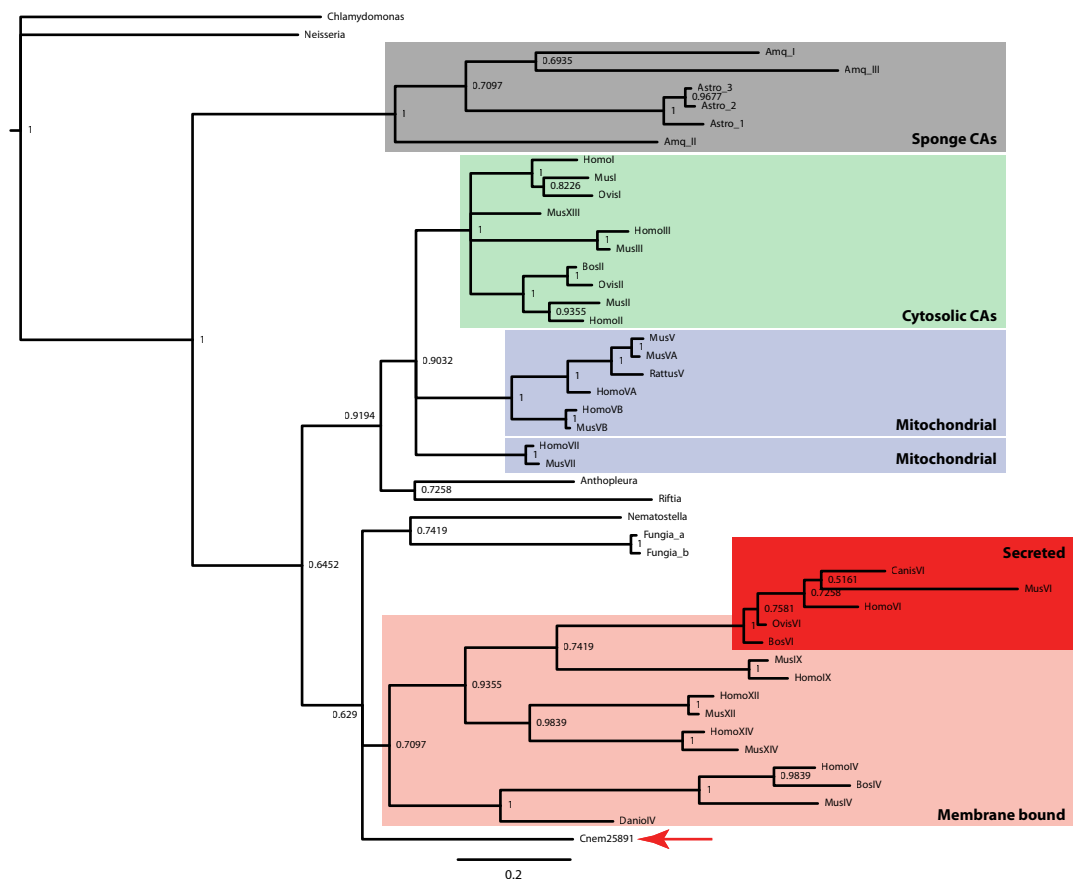

Supplement: Additional file 8 — A 50% majority rule consensus tree generated by Bayesian methods representing the phylogenetic relationships of metazoan CAs. [file 1471-2164-15-249-S8.pdf]

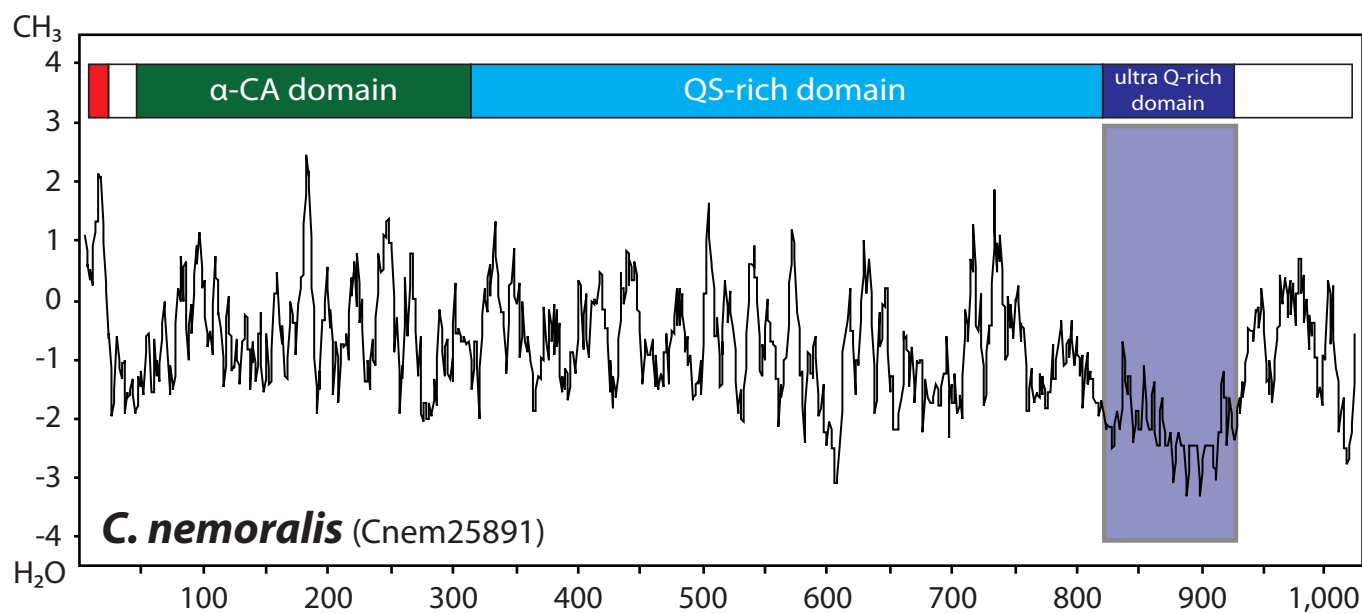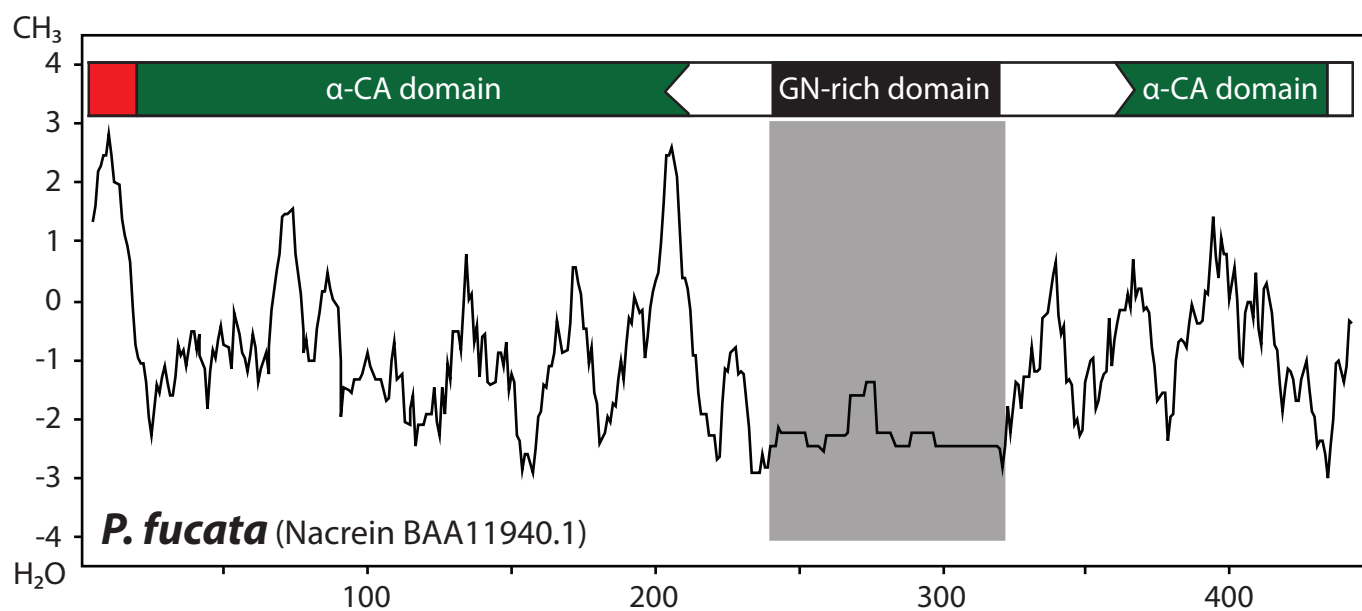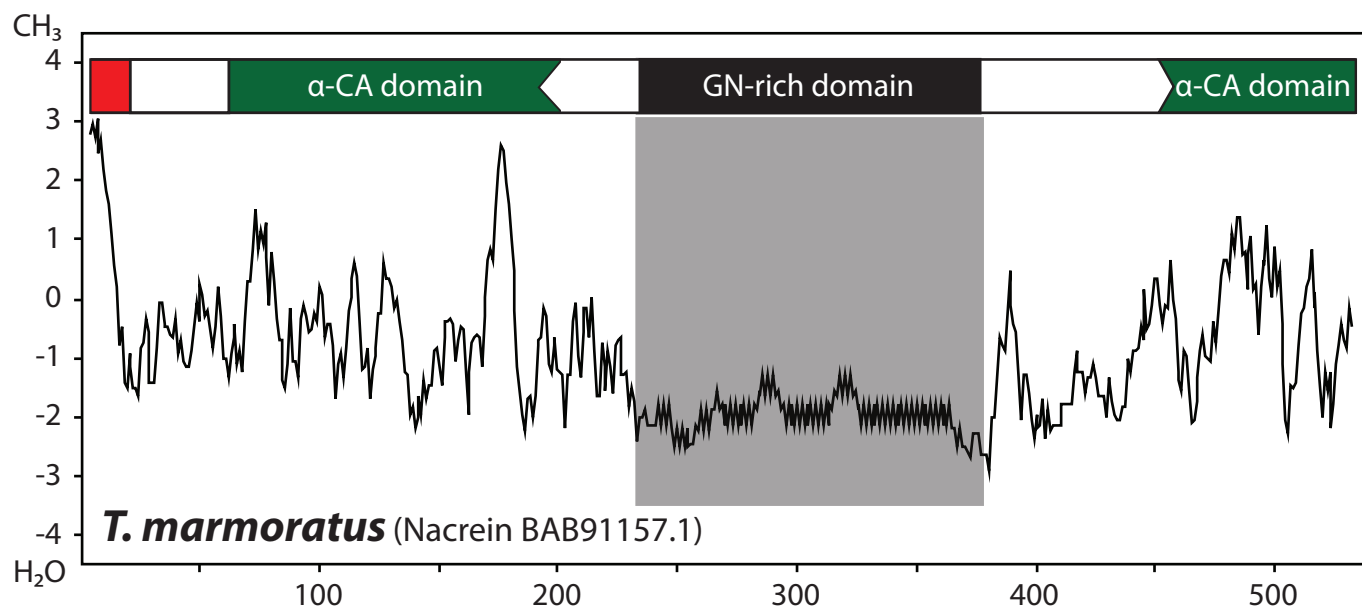

Supplement: Additional file 10 — Hydropathy profiles of Cnem25891 and two previously reported molluscan shell-forming proteins which also posses CA domains. [file 1471-2164-15-249-S10.pdf]
