# Supplementary material for: Characterization of the pigmented shell-forming proteome of the common grove snail Cepaea nemoralis
Source: BMC Genomics. 2014 Mar 31;15:249. doi: 10.1186/1471-2164-15-249 (PMC4023409; doi:10.1186/1471-2164-15-249)
Supplement: Additional file 7 — Top BLASTp hits returned against C. nemoralis queries from five molluscan shell proteomes and Swissprot. [file 1471-2164-15-249-S7.docx]

**Additional file 7. Top BLASTp hits returned against *C. nemoralis* queries from five molluscan shell proteomes and Swissprot.**

Rows are grouped by shading according to the number of different shell-proteomes each query has similarity to.

| **Query** | ***L. gigantea* ^1^**  **(e-value)** | ***C. gigas***  **(e-value)** | ***P. margaritifera***  **(e-value)** | ***P. maxima***  **(e-value)** | ***H. asinina***  **(e-value)** | **SwissProt**  **(e-value): accession** |
| --- | --- | --- | --- | --- | --- | --- |
| **Cnem821** | 232022 (1e-08) | EKC35875.1 (8e-20) | PUSP15^2^ (2e-15) | EZ420121 (2e-10) | - | - |
| **Cnem248122** | 232880 (9e-06) | EKC38805.1 (1e-18) | HE610381 (3e-09) | EZ420284 (2e-07) | - | Chitotriosidase-1 (4e-22): Q13231 |
| **Cnem63304** | 231869 (2e-90) | EKC38530.1 (7e-20) | PUSP16^2^ (6e-24) | - | - | - |
| **Cnem14003** | 231869 (3e-49) | EKC38530.1 (1e-15) | PUSP16^2^ (6e-16) | - | - | - |
| **Cnem101824** | 231869 (3e-67) | EKC38530.1 (7e-14) | PUSP16^2^ (6e-11) | - | - | - |
| **Cnem2668** | 156525 (3e-6) | EKC35875.1 (7e-08) | PUSP12^2^ (6e-9) | - | - | Selectin-like osteoblast-derived protein (2e-08):Q4LDE5 |
| **Cnem25891** | 66515 (6e-74) | EKC19847.1 (1e-27) | - | - | - | Carbonic anhydrase 2 (2e-58): P00920 |
| **Cnem3883** | 201878 (3e-27) | EKC39411.1 (4e-25) | - | - | - | V-ATPase subunit A (0.0): P38606 |
| **Cnem1188** | 190352 (4e-49) | EKC42376.1 (1e-46) | - | - | - | MSP130 (1e-10):P08472 |
| **Cnem20360** | 229248 (1e-23) | EKC39436.1 (1e-33) | - | - | - | dN-cadherin (3e-47):O15943 |
| **Cnem104312** | 205401 (2e-07) | EKC41746.1 (5e-11) | - | - | - | Carbonic anhydrase-related protein 10 (6e-07): A0JN41 |
| **Cnem1237** | 201878 (2e-24) | EKC39411.1 (5e-24) | - | - | - | V-ATPase subunit B (0.0): P31401 |
| **Cnem4282** | 190352 (2e-29) | EKC42376.1 (2e-13) | - | - | - | Gigasin-3a (8e-21): P86786 |
| **Cnem7809** | 235120 (4e-08) | - | - | - | - | - |
| **Cnem691** | - | EKC37598.1 (2e-33) | - | - | - | Ficolin-3 (5e-35): O75636 |
| **Cnem1647** | 156525 (1e-06) | - | - | - | - | - |
| **Cnem6176** | 231010 (1e-06) | - | - | - | - | - |
| **Cnem1323** | 181237 (9e-30) | - | - | - | - | - |
| **Cnem31170** | 181237 (2e-41) | - | - | - | - | - |
| **Cnem5087** | 163637 (3e-14) ^3^ | - | - | - | - | Late embryogenesis abundant protein 76 (3e-08): P13934^3^ |
| **Cnem450** | B3A0P5.1 (2e-58) | - | - | - | - | Lysozyme (1e-36): P00717 |
| **Cnem12941** | 235120 (1e-07) | - | - | - | - | - |
| **Cnem7508** | 234386 (4e-37) | - | - | - | - | - |
| **Cnem4164** | - | - | - | - | GT272916 (1e-07) | - |

^1^ *L. gigantea* protein IDs taken from http://genome.jgi-psf.org/Lotgi1/Lotgi1.home.html.

^2^ See [30] for sequence details.

^3^ This similarity is most likely due to the presence of a high proportion of a single amino acid rather than evolutionary homology.
