# Supplementary material for: Characterization of the pigmented shell-forming proteome of the common grove snail Cepaea nemoralis
Source: BMC Genomics. 2014 Mar 31;15:249. doi: 10.1186/1471-2164-15-249 (PMC4023409; doi:10.1186/1471-2164-15-249)
Supplement: Additional file 9 — The Nucleotide and derived protein sequence of Cnem25891. [file 1471-2164-15-249-S9.pdf]

```
   1 - CAGCAACTTCCTGCAGTTTTCATCGTGGATTCACTGTGCGCGCATTCTCATCTGCCGGCT - 60
  61 - TTTGATATACCTGAGAGCTGACGCTCCAGAAAAGGTAACTGGTGTTTGCAACGCAAGGAC - 120
 121 - TTAGTCTATTAAGCGCAGTCAGTCTGTAGTTGTCTTTAGTTGAGGTGGACCAGTTAGGGA - 180
 181 - AGTTGTGGACAGGTGTTTGGGACTGAGTGAGATTGTAAAACGTGTGCAGTTGCAGGATTA - 240
 241 - GCAGGACCAGGAGAATATAAAGGAATAGGAGCGTTAGGTTATGGTTTGTGGACAAAGCGG - 300
 301 - CAAGTATTCCAGATTTCCATTGCGCTTTTTGCAAGAAGCTGGTATTCTGCCGTGCGAGTT - 360
 361 - GACATCTGGCTTTGATGTTTCAGATACACTCGCTCACAGTCATCGCTTAGAATTTTGCCT - 420
 421 - GCTATCTACGACAGCCGTTCTGTGAGTGCTCTAGGCAACCCTTCTCTGAGTACTGTAAGC - 480
 481 - GTGCAACGCTCTATCCAGCACGAAAAACAGCAGCAGAGGAGGCAGCTATATAACAGTGTC - 540
 541 - ACTGTACGAGGCGGCATCAACAACTAGACCAGCAATTGTAAGGGATACATACATCAACAG - 600
 601 - ACGACAAAATGGCGCCAGTAGCGGCAGCTAATGCCATGACGAAGATTACGCTTTCTTTCT - 660
   1 -          M  A  P  V  A  A  A  N  A  M  T  K  I  T  L  S  F  L   - 18
 661 - TGTTGTCTTTGCTTGGTGATAAAAACTTCCAAGGGCCCGAGGCTCCAGTCAATAGCAAAT - 720
  19 -   L  S  L  L  G  D  K  N  F  Q  G  P  E  A  P  V  N  S  K  Y   - 38
 721 - ATCACTGGGCATATAAGGGACCAGAAGGTCCAGACTCTTGGCATGTGCACTACAAATACT - 780
  39 -   H  W  A  Y  K  G  P  E  G  P  D  S  W  H  V  H  Y  K  Y  C   - 58
 781 - GTGCTGGCGAGCGTCAGTCTCCTATCAACATCAAGACCTCCAGTTGTGAGTACGATCCAA - 840
  59 -   A  G  E  R  Q  S  P  I  N  I  K  T  S  S  C  E  Y  D  P  R   - 78
 841 - GGTTAGAGCCTATCATTCTAGAAAATTTTGACGCCACGAGTGGATCAGCAGGCCCTTGTA - 900
  79 -   L  E  P  I  I  L  E  N  F  D  A  T  S  G  S  A  G  P  C  I   - 98
 901 - TTCTTAATGTCACTAACAACGGTCATGCTGCCAGTGTCCGAGTGCTCAACCAGGACATGC - 960
  99 -   L  N  V  T  N  N  G  H  A  A  S  V  R  V  L  N  Q  D  M  H   - 118
 961 - ACGTGAGAGGTGGAGGGTTGGGCCAGGATTATAAAACGGTCGAGTTTCACTTCCACTGGG - 1020
 119 -   V  R  G  G  G  L  G  Q  D  Y  K  T  V  E  F  H  F  H  W  G   - 138
1021 - GCAGCACTGATGATGTTGGCAGTGAGCATGCAGTCAATGGCAAGAAATATCCTTTAGAGA - 1080
 139 -   S  T  D  D  V  G  S  E  H  A  V  N  G  K  K  Y  P  L  E  M   - 158
1081 - TGCATGTAGTAAACTATGCAGAGAAATATGGCAACGTGAAAGCAGCGATGACACAGCCCG - 1140
 159 -   H  V  V  N  Y  A  E  K  Y  G  N  V  K  A  A  M  T  Q  P  D   - 178
1141 - ACGGACTGGCAGTTCTCGGTGTCTTTTTTGAGATTACGGAGAAGGATAATCCTTACTTCG - 1200
 179 -   G  L  A  V  L  G  V  F  F  E  I  T  E  K  D  N  P  Y  F  A   - 198
1201 - CGGCTGTAGACGAATCTCTGCGACATATTCACAAAGCAGGCGACCACGCGACCATCAACC - 1260
 199 -   A  V  D  E  S  L  R  H  I  H  K  A  G  D  H  A  T  I  N  Q   - 218
1261 - AACTACGCCTGATGTCGCTCCTACCTGATGACCTCTCCAAGTTCTGGAGGTACAACGGCT - 1320
 219 -   L  R  L  M  S  L  L  P  D  D  L  S  K  F  W  R  Y  N  G  S   - 238
1321 - CGCTGACCACACCCTTCTGCTTCGAATCCGTTATATGGACTGTATTTACGGAACCGCAGA - 1380
 239 -   L  T  T  P  F  C  F  E  S  V  I  W  T  V  F  T  E  P  Q  K   - 258
1381 - AGATTTCGAAATCCCAGCTGGAAATACTACGAACACTCCTACACGAAGAAGGTCACGATA - 1440
 259 -   I  S  K  S  Q  L  E  I  L  R  T  L  L  H  E  E  G  H  D  I   - 278
1441 - TAAGCGATCGAGGGGAGCACCACTCCTTCACACCATCCAGGCTTGTCGATAACTGGCGCC - 1500
 279 -   S  D  R  G  E  H  H  S  F  T  P  S  R  L  V  D  N  W  R  P   - 298
1501 - CACTCCAACCACTCAACGGCCGGGTGATCAAGCAGAGTTTTCGCGGGGATTTTATACACA - 1560
 299 -   L  Q  P  L  N  G  R  V  I  K  Q  S  F  R  G  D  F  I  H  N   - 318
1561 - ACAAGCCTCCCCTGGACACAAAGATCGCCTCATTTGAAGCGTCCGCGCCGACCTTGGCAA - 1620
 319 -   K  P  P  L  D  T  K  I  A  S  F  E  A  S  A  P  T  L  A  I   - 338
1621 - TAGCCAATAAACCCGATAGTTTAGGAGGAACTGTAATGTACAACATAAACCCTACGTTTC - 1680
 339 -   A  N  K  P  D  S  L  G  G  T  V  M  Y  N  I  N  P  T  F  Q   - 358
1681 - AACCAATGGCACCACAAGAGATGACAAAAGATAAAGCAATGCCACAGGCAAGCAGTAAAA - 1740
 359 -   P  M  A  P  Q  E  M  T  K  D  K  A  M  P  Q  A  S  S  K  I   - 378
1741 - TACAGGCTCGAATCACGGAAATTAATAATCTTAAATCACAGGAAAGTTTTGGAAGAGGTC - 1800
 379 -   Q  A  R  I  T  E  I  N  N  L  K  S  Q  E  S  F  G  R  G  P   - 398
1801 - CGGTGGAAGAGAAGCTGTTGATATACAACCAGGGTCCCTCTGTAGAAATGGGAGTGAATT - 1860
 399 -   V  E  E  K  L  L  I  Y  N  Q  G  P  S  V  E  M  G  V  N  S   - 418
1861 - CTTCACCTATCACATTAAGCTCGGAGAAGTCTCAGCAAGTTGAGGTGAACAGAGGCATAG - 1920
 419 -   S  P  I  T  L  S  S  E  K  S  Q  Q  V  E  V  N  R  G  I  G   - 438
1921 - GCCTGGCGGATGCCCCCGCGACAACTCTGTTTGGACAGATCCAGCAATCGAACCAAGCTC - 1980
 439 -   L  A  D  A  P  A  T  T  L  F  G  Q  I  Q  Q  S  N  Q  A  L   - 458
1981 - TGACCGCTAACAATGGACCTGAGTCCAGCGTACATCACCCAGGGGAGGCAAGTGCTCAGA - 2040
 459 -   T  A  N  N  G  P  E  S  S  V  H  H  P  G  E  A  S  A  Q  M   - 478
2041 - TGTCCTCGAGTTCCGCATTAAAAGCTGATCTAAAAAAGGATGTGGGTAGACTAGAGCAAA - 2100
 479 -   S  S  S  S  A  L  K  A  D  L  K  K  D  V  G  R  L  E  Q  N   - 498
2101 - ACAACCCAATAACGTCTCCAGTTGTTAATATCGTTTCAAGACCAAGACATGTGTCTTCTT - 2160
 499 -   N  P  I  T  S  P  V  V  N  I  V  S  R  P  R  H  V  S  S  S   - 518
2161 - CTGGAGCTAACGGCGTTTTAAGAAATGTTCAAAGCCAGCAAAGTTCAAATTCTGGAAAAG - 2220
 519 -   G  A  N  G  V  L  R  N  V  Q  S  Q  Q  S  S  N  S  G  K  V   - 538
2221 - TGACAATGGTGATAAATACTAGTACTACAACTCCGTCTTCTCGTGTTCATGTTTCATTAC - 2280
 539 -   T  M  V  I  N  T  S  T  T  T  P  S  S  R  V  H  V  S  L  R   - 558
2281 - GTCCTAATCGTCTACAAAGTTCTGAGATGCAGCCATCGGTTCAAGTGTTACCAATTGCCC - 2340
 559 -   P  N  R  L  Q  S  S  E  M  Q  P  S  V  Q  V  L  P  I  A  Q   - 578
2341 - AACAGCAAAAAGCTCAAAGCTCAGAAGCAGCTGGGCGTTCTCAACCTCTACCACCGAAAC - 2400
 579 -   Q  Q  K  A  Q  S  S  E  A  A  G  R  S  Q  P  L  P  P  K  Q   - 598
2401 - AACAAACTCAAATGCAGCCAAACACAAACCGCCAGCAGCAACAATCAGCGTCTGCTGTGT - 2460
 599 -   Q  T  Q  M  Q  P  N  T  N  R  Q  Q  Q  Q  S  A  S  A  V  S   - 618
2461 - CCCAGTCAAATCTCCGTTCTGGCCAAATAGATAGTCGCATAGCCGCTCTAGCTAACAACA - 2520
 619 -   Q  S  N  L  R  S  G  Q  I  D  S  R  I  A  A  L  A  N  N  I   - 638
2521 - TCAGGTCTCATCGCCACCTAGCAATAACACAAATCCAACCAAACACACAGCCAGCAAACA - 2580
 639 -   R  S  H  R  H  L  A  I  T  Q  I  Q  P  N  T  Q  P  A  N  N   - 658
2581 - ACAATCCCCAAATGCCTCCAGTGATTCGACACAATAAACAGATTCCCCCAGCGATGCAGA - 2640
 659 -   N  P  Q  M  P  P  V  I  R  H  N  K  Q  I  P  P  A  M  Q  N   - 678
2641 - ACCAACAACGTGACTTGATATCGCAAAGACAACAGCAATATTTGCTACAGCAACCTCCAC - 2700
```

```
 679 -   Q  Q  R  D  L  I  S  Q  R  Q  Q  Q  Y  L  L  Q  Q  P  P  Q   - 698
2701 - AACAAATTCAACGCATGAGCAGACCGCCAACTCCGCAACAGCTACGAGCAATATTCGAGA - 2760
 699 -   Q  I  Q  R  M  S  R  P  P  T  P  Q  Q  L  R  A  I  F  E  K   - 718
2761 - AGTTGGCTGCATTCAGGCGCGCAAACGCACGGTCTAGAATAATGACATCAGGAGCAGCGA - 2820
 719 -   L  A  A  F  R  R  A  N  A  R  S  R  I  M  T  S  G  A  A  I   - 738
2821 - TAATCCCCCGACTCAAACTACAACAAGTGAATTCAGTAAGTGGTACGCGATTCCTTAGCA - 2880
 739 -   I  P  R  L  K  L  Q  Q  V  N  S  V  S  G  T  R  F  L  S  N   - 758
2881 - ATCCTGCTTCTTCTAGGCATCACACAGGTGCGTCAAATGGAAACCAGCAATTTAACATTC - 2940
 759 -   P  A  S  S  R  H  H  T  G  A  S  N  G  N  Q  Q  F  N  I  P   - 778
2941 - CTCGAGACAATTCCCAGTTCCTTTCTCCTGAACCGTCCTCATCCTTGCAGTCATCGTGGA - 3000
 779 -   R  D  N  S  Q  F  L  S  P  E  P  S  S  S  L  Q  S  S  W  T   - 798
3001 - CAAACATAAACACAAACCTGGACTACAACGGTCAGGCTGGCTCCTCGTATCAACCACCTC - 3060
 799 -   N  I  N  T  N  L  D  Y  N  G  Q  A  G  S  S  Y  Q  P  P  Q   - 818
3061 - AATCATATTGGTCACAGCCCTCTCAGCAACAAACATACTCACAGCGGACTGGATACCAAC - 3120
 819 -   S  Y  W  S  Q  P  S  Q  Q  Q  T  Y  S  Q  R  T  G  Y  Q  Q   - 838
3121 - AGAGCATGCCACTACAACAAAACACACAGCAACAGCAGGTGTACCCCCAACAACAGCAGG - 3180
 839 -   S  M  P  L  Q  Q  N  T  Q  Q  Q  Q  V  Y  P  Q  Q  Q  Q  V   - 858
3181 - TATACCCACAGCAACAGCAGGCATACCCACAGCAACAGCAGGCATACCCCCAACAACAAC - 3240
 859 -   Y  P  Q  Q  Q  Q  A  Y  P  Q  Q  Q  Q  A  Y  P  Q  Q  Q  Q   - 878
3241 - AACCGCAACAGCAGGCATACCCACAACAACAGCAGCAGCAACAGCAGGCATACCCACAAC - 3300
 879 -   P  Q  Q  Q  A  Y  P  Q  Q  Q  Q  Q  Q  Q  Q  A  Y  P  Q  Q   - 898
3301 - AACAGCAGCAGCAACAGCAGGCATACCCCCAACAACAACAGCAGGATTACCCGCAACTAC - 3360
 899 -   Q  Q  Q  Q  Q  Q  A  Y  P  Q  Q  Q  Q  Q  D  Y  P  Q  L  Q   - 918
3361 - AGCAGGCATACCCCCAACAACAGCAGGCGTACACGCAGCAACAGCCAACCCCAACTCTCC - 3420
 919 -   Q  A  Y  P  Q  Q  Q  Q  A  Y  T  Q  Q  Q  P  T  P  T  L  H   - 938
3421 - ATTACACCCCAGCATCCCCCACTGCCATGTACTCCTCCCCCATGGCCAGATCAGACAACA - 3480
 939 -   Y  T  P  A  S  P  T  A  M  Y  S  S  P  M  A  R  S  D  N  T   - 958
3481 - CAAACCTCTACTACACAGCAGGCCCTGGCGTGTCGGGCGCCAGTTCAAACGCTATGTCTC - 3540
 959 -   N  L  Y  Y  T  A  G  P  G  V  S  G  A  S  S  N  A  M  S  L   - 978
3541 - TACCTCAAGTGTCGGCCATGGACGCGAATGCTCACCAAGTCTATCTGAACAACCAAAAAC - 3600
 979 -   P  Q  V  S  A  M  D  A  N  A  H  Q  V  Y  L  N  N  Q  K  Q   - 998
3601 - AGCCAGTGCGGATATTACGTCCAGTTTATGAGCAAGTCAACAGCAGGCAACAAGCACAGT - 3660
 999 -   P  V  R  I  L  R  P  V  Y  E  Q  V  N  S  R  Q  Q  A  Q  Y   - 1018
3661 - ACCAGAACCAGCAGTACCCGTTCTTCGTTTACTGAGTGCGCAACATCTGTCTTATGCTCA - 3720
1019 -   Q  N  Q  Q  Y  P  F  F  V  Y  *                                - 1038
3721 - CTGCTGATCTGTTTTATCATCACCATCATAACCACCGATCTGTCTTACCTTGATTTGTCT - 3780
3781 - TGTCATCACTTGTCTGTCTTATCATCATCACCGATCTGTCTTATTATCACTGATATGTCT - 3840
3841 - TATCATTACTGGACTGTCT                                          - 3859
```
