# Supplementary material for: Characterization of the pigmented shell-forming proteome of the common grove snail Cepaea nemoralis
Source: BMC Genomics. 2014 Mar 31;15:249. doi: 10.1186/1471-2164-15-249 (PMC4023409; doi:10.1186/1471-2164-15-249)
Supplement: Additional file 12 — The Circoletto generated “*.blasted” file used to generate the Circos figure (Figure 4). [file 1471-2164-15-249-S12.pdf]

```
# BLASTP 2.2.26 [Sep-21-2011]
# Query: Cnem25891
# Database: X
# Fields: Query id, Subject id, % identity, alignment length, mismatches, gap op
Cnem25891   Lgig66515   50.92   218 104 1   47  264 2   216 6e-74    239
Cnem25891   Lgig205401  36.36   275 155 7   40  312 4   260 8e-46    161
Cnem25891   CgigEKC19847.1  29.14   278 162 9   40  308 61  312 1e-27    116
Cnem25891   CgigEKC41746.1  30.50   141 74  4   159 282 100 233 5e-12   63.2
Cnem25891   CgigEKC34226.1  27.47   233 111 13  1   193 1   215 8e-10   58.2
Cnem25891   LgigB3A0P2.1    39.56   91  40  4   224 312 290 367 8e-08   51.2
Cnem25891   LgigB3A0P2.1    32.35   68  45  1   121 187 126 193 7e-06   45.1
Cnem25891   Lgig238082  39.56   91  40  4   224 312 290 367 8e-08   51.2
Cnem25891   Lgig238082  32.35   68  45  1   121 187 126 193 7e-06   45.1
# BLASTP 2.2.26 [Sep-21-2011]
# Query: Cnem84589
# Database: /Users/danieljackson/Documents/Data/Manuscripts/2013/Cepaea/Figures/
# Fields: Query id, Subject id, % identity, alignment length, mismatches, gap op
Cnem84589   Lgig231869  39.02   451 258 7   383 819 11  458 3e-93    313
Cnem84589   Lgig231869  31.36   456 275 10  246 679 15  454 6e-59    214
Cnem84589   Lgig231869  30.04   466 279 16  389 823 611 1060    4e-51    190
Cnem84589   Lgig231869  28.51   456 258 16  261 677 624 1050    6e-40    155
Cnem84589   Lgig231869  26.84   395 242 10  535 913 612 975 1e-30    125
Cnem84589   Lgig231869  26.70   397 235 13  533 908 16  377 3e-27    114
Cnem84589   Lgig231869  26.15   348 209 14  205 526 725 1050    3e-19   89.0
Cnem84589   Lgig231869  36.90   84  51  2   831 912 596 679 5e-09   55.5
Cnem84589   Lgig160173  31.97   244 156 4   147 385 4   242 2e-32    122
Cnem84589   Lgig160173  27.54   167 106 4   373 532 86  244 8e-12   62.4
Cnem84589   Lgig160173  29.55   176 103 7   513 681 81  242 6e-11   59.3
Cnem84589   CgigEKC38530.1  38.26   149 76  5   394 530 101 245 7e-23   97.8
Cnem84589   CgigEKC38530.1  28.98   176 116 3   530 699 92  264 4e-16   77.4
Cnem84589   CgigEKC38530.1  38.04   92  50  2   690 774 101 192 3e-10   58.9
Cnem84589   Lgig234405  29.07   258 148 10  356 595 57  297 2e-22   94.7
Cnem84589   Lgig234405  30.21   192 110 5   499 675 43  225 2e-20   89.4
Cnem84589   PmargPUSP16 36.00   150 78  5   402 537 141 286 2e-21   94.0
Cnem84589   PmargPUSP16 34.06   138 83  3   531 664 122 255 1e-15   75.9
Cnem84589   PmargPUSP16 34.55   110 63  3   697 797 140 249 2e-10   59.3
Cnem84589   PmargPUSP16 30.18   169 82  9   253 392 133 294 1e-06   47.8
# BLASTP 2.2.26 [Sep-21-2011]
# Query: Cnem63304
# Database: /Users/danieljackson/Documents/Data/Manuscripts/2013/Cepaea/Figures/
# Fields: Query id, Subject id, % identity, alignment length, mismatches, gap op
Cnem63304   Lgig231869  36.62   497 284 7   293 761 15  508 2e-90    305
Cnem63304   Lgig231869  32.34   436 260 10  152 565 15  437 6e-61    220
Cnem63304   Lgig231869  30.15   461 275 17  295 724 611 1055    1e-48    182
Cnem63304   Lgig231869  28.03   471 280 18  149 583 603 1050    4e-41    159
Cnem63304   Lgig231869  26.47   476 294 13  441 894 612 1053    1e-35    141
Cnem63304   Lgig231869  25.71   455 285 13  439 873 16  437 2e-31    128
Cnem63304   Lgig231869  31.41   156 103 3   752 904 611 765 2e-17   82.8
Cnem63304   Lgig231869  22.29   341 203 11  591 904 17  322 2e-13   70.5
Cnem63304   Lgig160173  32.92   243 149 6   60  292 5   243 4e-35    130
Cnem63304   Lgig160173  29.21   178 105 5   419 589 81  244 8e-11   59.3
```

```
Cnem63304   Lgig160173  26.67   195 125 6   248 432 52  238 1e-09   55.8
Cnem63304   Lgig160173  25.87   143 87  5   756 892 110 239 1e-07   49.7
Cnem63304   PmargPUSP16 34.13   167 96  4   293 448 126 289 6e-24    101
Cnem63304   PmargPUSP16 33.33   138 82  3   452 583 140 273 2e-15   75.1
Cnem63304   PmargPUSP16 32.14   112 67  3   603 705 140 251 2e-09   56.2
Cnem63304   PmargPUSP16 23.96   192 122 5   684 853 40  229 9e-07   47.8
Cnem63304   CgigEKC38530.1  37.32   142 76  4   281 412 78  216 7e-20   88.6
Cnem63304   CgigEKC38530.1  28.95   190 122 4   426 605 78  264 2e-17   80.9
Cnem63304   CgigEKC38530.1  32.50   120 70  4   596 705 101 219 6e-09   54.7
Cnem63304   CgigEKC38530.1  25.79   190 119 5   684 853 10  197 3e-06   45.8
Cnem63304   Lgig234405  35.04   117 74  1   312 426 105 221 7e-17   78.2
Cnem63304   Lgig234405  26.32   190 120 5   405 581 43  225 2e-15   73.6
Cnem63304   Lgig234405  24.84   310 179 14  76  351 6   295 2e-08   52.4
# BLASTP 2.2.26 [Sep-21-2011]
# Query: Cnem691
# Database: /Users/danieljackson/Documents/Data/Manuscripts/2013/Cepaea/Figures/
# Fields: Query id, Subject id, % identity, alignment length, mismatches, gap op
Cnem691 CgigEKC37598.1  36.02   186 105 5   582 759 19  198 2e-33    123
Cnem691 CgigEKC37595.1  37.10   186 103 5   582 759 374 553 2e-29    119
Cnem691 CgigEKC37595.1  42.31   130 70  3   602 730 163 288 2e-25    107
# BLASTP 2.2.26 [Sep-21-2011]
# Query: Cnem3883
# Database: /Users/danieljackson/Documents/Data/Manuscripts/2013/Cepaea/Figures/
# Fields: Query id, Subject id, % identity, alignment length, mismatches, gap op
Cnem3883    Lgig201878  28.22   365 224 11  210 567 126 459 3e-27    111
Cnem3883    CgigEKC39411.1  28.57   322 203 10  253 567 51  352 4e-25    103
Cnem3883    Lgig206617  26.59   252 153 9   265 501 183 417 6e-15   73.2
Cnem3883    CgigEKC39329.1  25.40   252 156 8   265 501 183 417 4e-14   70.9
# BLASTP 2.2.26 [Sep-21-2011]
# Query: Cnem20308
# Database: /Users/danieljackson/Documents/Data/Manuscripts/2013/Cepaea/Figures/
# Fields: Query id, Subject id, % identity, alignment length, mismatches, gap op
# BLASTP 2.2.26 [Sep-21-2011]
# Query: Cnem7323
# Database: /Users/danieljackson/Documents/Data/Manuscripts/2013/Cepaea/Figures/
# Fields: Query id, Subject id, % identity, alignment length, mismatches, gap op
# BLASTP 2.2.26 [Sep-21-2011]
# Query: Cnem2357
# Database: /Users/danieljackson/Documents/Data/Manuscripts/2013/Cepaea/Figures/
# Fields: Query id, Subject id, % identity, alignment length, mismatches, gap op
# BLASTP 2.2.26 [Sep-21-2011]
# Query: Cnem1850
# Database: /Users/danieljackson/Documents/Data/Manuscripts/2013/Cepaea/Figures/
# Fields: Query id, Subject id, % identity, alignment length, mismatches, gap op
# BLASTP 2.2.26 [Sep-21-2011]
# Query: Cnem1237
# Database: /Users/danieljackson/Documents/Data/Manuscripts/2013/Cepaea/Figures/
# Fields: Query id, Subject id, % identity, alignment length, mismatches, gap op
Cnem1237    Lgig201878  24.60   435 278 11  77  498 89  486 2e-24    102
Cnem1237    CgigEKC39411.1  26.26   377 244 9   90  457 1   352 5e-24    100
Cnem1237    CgigEKC39329.1  26.05   311 202 8   106 404 135 429 8e-23   97.4
```

```
Cnem1237    Lgig206617  25.33   304 209 6   106 402 135 427 5e-21   92.0
# BLASTP 2.2.26 [Sep-21-2011]
# Query: Cnem2668
# Database: /Users/danieljackson/Documents/Data/Manuscripts/2013/Cepaea/Figures/
# Fields: Query id, Subject id, % identity, alignment length, mismatches, gap op
Cnem2668    PmargPUSP12 31.69   142 83  7   69  209 31  159 6e-09   52.0
Cnem2668    CgigEKC35875.1  32.20   118 74  5   93  209 769 881 7e-08   50.8
Cnem2668    CgigEKC35875.1  26.67   135 89  7   89  221 881 1007    2e-06   46.2
Cnem2668    CgigEKC35875.1  23.98   196 138 7   93  282 1742    1932    8e-06
Cnem2668    CgigEKC35875.1  30.19   106 67  5   106 209 1297    1397    9e-06
Cnem2668    CgigEKC32319.1  33.33   108 59  5   110 208 580 683 1e-06   46.6
Cnem2668    Lgig156525  25.21   242 148 10  65  298 601 817 3e-06   45.1
# BLASTP 2.2.26 [Sep-21-2011]
# Query: Cnem31170
# Database: /Users/danieljackson/Documents/Data/Manuscripts/2013/Cepaea/Figures/
# Fields: Query id, Subject id, % identity, alignment length, mismatches, gap op
Cnem31170   Lgig181237  30.53   321 200 13  149 464 27  329 2e-41    146
# BLASTP 2.2.26 [Sep-21-2011]
# Query: Cnem7807
# Database: /Users/danieljackson/Documents/Data/Manuscripts/2013/Cepaea/Figures/
# Fields: Query id, Subject id, % identity, alignment length, mismatches, gap op
# BLASTP 2.2.26 [Sep-21-2011]
# Query: Cnem572
# Database: /Users/danieljackson/Documents/Data/Manuscripts/2013/Cepaea/Figures/
# Fields: Query id, Subject id, % identity, alignment length, mismatches, gap op
Cnem572 Lgig204921  48.10   447 230 2   3   448 2   447 4e-143   414
Cnem572 Lgig109284  47.93   434 225 1   18  450 22  455 4e-140   405
# BLASTP 2.2.26 [Sep-21-2011]
# Query: Cnem101824
# Database: /Users/danieljackson/Documents/Data/Manuscripts/2013/Cepaea/Figures/
# Fields: Query id, Subject id, % identity, alignment length, mismatches, gap op
Cnem101824  Lgig231869  32.70   419 240 10  2   414 676 1058    3e-67    226
Cnem101824  Lgig231869  31.09   357 198 11  40  388 121 437 2e-43    158
Cnem101824  Lgig231869  29.05   327 197 10  86  409 18  312 1e-27    111
Cnem101824  Lgig231869  30.12   332 171 17  96  414 622 905 3e-26    107
Cnem101824  Lgig231869  26.72   247 150 8   1   244 234 452 4e-21   92.0
Cnem101824  Lgig231869  26.54   162 98  7   245 399 4   151 2e-06   45.8
Cnem101824  CgigEKC38530.1  29.70   165 86  5   96  252 107 249 7e-14   68.2
Cnem101824  CgigEKC38530.1  30.89   123 77  4   271 393 109 223 4e-09   53.5
Cnem101824  PmargPUSP16 29.94   157 80  5   96  244 139 273 6e-11   59.3
Cnem101824  PmargPUSP16 33.06   121 73  4   273 393 143 255 3e-09   53.9
Cnem101824  Lgig234405  24.09   328 188 14  20  324 15  304 3e-10   56.6
Cnem101824  Lgig160173  25.00   248 121 8   28  244 25  238 5e-10   55.5
Cnem101824  Lgig160173  26.58   237 128 12  197 409 27  241 6e-07   46.2
# BLASTP 2.2.26 [Sep-21-2011]
# Query: Cnem5087
# Database: /Users/danieljackson/Documents/Data/Manuscripts/2013/Cepaea/Figures/
# Fields: Query id, Subject id, % identity, alignment length, mismatches, gap op
Cnem5087    Lgig163637  24.08   353 228 7   76  394 83  429 3e-14   70.1
Cnem5087    Lgig163637  26.15   218 151 4   88  296 249 465 2e-12   64.3
Cnem5087    Lgig163637  25.43   232 149 5   106 317 40  267 2e-11   60.8
```

```
Cnem5087    Lgig163637  26.52   230 148 6   101 319 75  294 4e-11   60.5
# BLASTP 2.2.26 [Sep-21-2011]
# Query: Cnem227
# Database: /Users/danieljackson/Documents/Data/Manuscripts/2013/Cepaea/Figures/
# Fields: Query id, Subject id, % identity, alignment length, mismatches, gap op
# BLASTP 2.2.26 [Sep-21-2011]
# Query: Cnem20360
# Database: /Users/danieljackson/Documents/Data/Manuscripts/2013/Cepaea/Figures/
# Fields: Query id, Subject id, % identity, alignment length, mismatches, gap op
Cnem20360   CgigEKC39436.1  34.72   337 189 15  1   326 510 826 1e-33    127
Cnem20360   CgigEKC39436.1  30.77   195 117 6   1   181 721 911 1e-13   67.4
Cnem20360   CgigEKC39436.1  25.26   285 182 8   4   277 1060    1324    3e-11
Cnem20360   CgigEKC39436.1  26.54   162 100 6   151 304 766 916 5e-07   47.0
Cnem20360   Lgig229248  28.26   368 207 14  1   331 521 868 1e-23   98.2
Cnem20360   Lgig229248  26.75   329 198 14  1   298 762 1078    2e-16   75.9
Cnem20360   Lgig229248  25.53   380 226 16  1   333 154 523 9e-16   74.3
Cnem20360   Lgig229248  25.70   323 190 9   52  332 338 652 8e-13   65.1
Cnem20360   Lgig229248  28.35   261 161 12  99  341 1097    1349    4e-12   63.2
Cnem20360   Lgig229248  26.17   149 92  4   7   141 1227    1371    5e-06   43.9
Cnem20360   Lgig229248  26.90   342 198 16  14  326 1120    1438    1e-05   43.1
Cnem20360   Lgig229249  25.07   355 227 11  1   335 702 1037    9e-19   83.6
Cnem20360   Lgig229249  26.36   349 234 11  1   338 1033    1369    1e-18   83.2
Cnem20360   Lgig229249  29.19   346 204 15  1   326 481 805 1e-16   77.0
Cnem20360   Lgig229249  27.09   299 173 14  1   283 932 1201    4e-15   72.0
Cnem20360   Lgig229249  28.41   271 162 7   83  332 339 598 3e-12   63.5
Cnem20360   Lgig229249  22.91   371 212 12  4   335 35  370 1e-09   55.1
Cnem20360   Lgig229249  25.39   319 211 14  2   299 1253    1565    9e-09   52.8
Cnem20360   Lgig229249  24.71   340 217 14  16  333 612 934 3e-08   50.8
Cnem20360   Lgig106548  29.30   273 164 11  62  327 93  343 1e-10   58.2
# BLASTP 2.2.26 [Sep-21-2011]
# Query: Cnem14003
# Database: /Users/danieljackson/Documents/Data/Manuscripts/2013/Cepaea/Figures/
# Fields: Query id, Subject id, % identity, alignment length, mismatches, gap op
Cnem14003   Lgig231869  30.21   341 189 9   2   332 760 1061    3e-49    172
Cnem14003   Lgig231869  29.35   310 174 10  1   305 168 437 8e-31    119
Cnem14003   Lgig231869  29.97   327 186 12  7   326 22  312 2e-30    117
Cnem14003   Lgig231869  28.66   321 171 15  13  323 622 894 4e-23   96.3
Cnem14003   Lgig231869  30.13   156 96  5   171 323 607 752 5e-11   59.7
Cnem14003   Lgig231869  26.35   148 98  6   171 316 13  151 7e-06   43.1
Cnem14003   PmargPUSP16 32.14   168 84  5   2   161 128 273 6e-16   73.9
Cnem14003   CgigEKC38530.1  30.38   158 80  5   17  166 111 246 1e-15   72.8
Cnem14003   CgigEKC38530.1  28.46   130 84  4   155 284 78  198 3e-07   47.4
Cnem14003   Lgig234405  25.00   160 84  7   13  161 99  233 6e-06   42.7
# BLASTP 2.2.26 [Sep-21-2011]
# Query: Cnem35852
# Database: /Users/danieljackson/Documents/Data/Manuscripts/2013/Cepaea/Figures/
# Fields: Query id, Subject id, % identity, alignment length, mismatches, gap op
# BLASTP 2.2.26 [Sep-21-2011]
# Query: Cnem201
# Database: /Users/danieljackson/Documents/Data/Manuscripts/2013/Cepaea/Figures/
# Fields: Query id, Subject id, % identity, alignment length, mismatches, gap op
```

```
# BLASTP 2.2.26 [Sep-21-2011]
# Query: Cnem2858
# Database: /Users/danieljackson/Documents/Data/Manuscripts/2013/Cepaea/Figures/
# Fields: Query id, Subject id, % identity, alignment length, mismatches, gap op
# BLASTP 2.2.26 [Sep-21-2011]
# Query: Cnem32297
# Database: /Users/danieljackson/Documents/Data/Manuscripts/2013/Cepaea/Figures/
# Fields: Query id, Subject id, % identity, alignment length, mismatches, gap op
# BLASTP 2.2.26 [Sep-21-2011]
# Query: Cnem7809
# Database: /Users/danieljackson/Documents/Data/Manuscripts/2013/Cepaea/Figures/
# Fields: Query id, Subject id, % identity, alignment length, mismatches, gap op
Cnem7809    Lgig235120  24.48   290 195 3   16  303 158 425 4e-08   50.1
# BLASTP 2.2.26 [Sep-21-2011]
# Query: Cnem132
# Database: /Users/danieljackson/Documents/Data/Manuscripts/2013/Cepaea/Figures/
# Fields: Query id, Subject id, % identity, alignment length, mismatches, gap op
# BLASTP 2.2.26 [Sep-21-2011]
# Query: Cnem1647
# Database: /Users/danieljackson/Documents/Data/Manuscripts/2013/Cepaea/Figures/
# Fields: Query id, Subject id, % identity, alignment length, mismatches, gap op
Cnem1647    Lgig156525  33.33   108 59  5   3   105 658 757 1e-06   45.1
Cnem1647    Lgig156525  32.26   93  45  7   1   80  591 678 5e-06   43.1
# BLASTP 2.2.26 [Sep-21-2011]
# Query: Cnem269
# Database: /Users/danieljackson/Documents/Data/Manuscripts/2013/Cepaea/Figures/
# Fields: Query id, Subject id, % identity, alignment length, mismatches, gap op
# BLASTP 2.2.26 [Sep-21-2011]
# Query: Cnem10584
# Database: /Users/danieljackson/Documents/Data/Manuscripts/2013/Cepaea/Figures/
# Fields: Query id, Subject id, % identity, alignment length, mismatches, gap op
# BLASTP 2.2.26 [Sep-21-2011]
# Query: Cnem1604
# Database: /Users/danieljackson/Documents/Data/Manuscripts/2013/Cepaea/Figures/
# Fields: Query id, Subject id, % identity, alignment length, mismatches, gap op
# BLASTP 2.2.26 [Sep-21-2011]
# Query: Cnem58150
# Database: /Users/danieljackson/Documents/Data/Manuscripts/2013/Cepaea/Figures/
# Fields: Query id, Subject id, % identity, alignment length, mismatches, gap op
# BLASTP 2.2.26 [Sep-21-2011]
# Query: Cnem7508
# Database: /Users/danieljackson/Documents/Data/Manuscripts/2013/Cepaea/Figures/
# Fields: Query id, Subject id, % identity, alignment length, mismatches, gap op
Cnem7508    Lgig234386  35.00   220 133 4   28  240 88  304 4e-37    128
Cnem7508    Lgig234387  32.89   228 142 6   17  238 26  248 2e-29    107
# BLASTP 2.2.26 [Sep-21-2011]
# Query: Cnem248122
# Database: /Users/danieljackson/Documents/Data/Manuscripts/2013/Cepaea/Figures/
# Fields: Query id, Subject id, % identity, alignment length, mismatches, gap op
Cnem248122  CgigEKC38805.1  32.92   161 99  4   3   160 352 506 1e-18   80.1
Cnem248122  CgigEKC38805.1  34.78   46  30  0   104 149 642 687 8e-06   42.0
```

```
Cnem248122  PmargHE610381   41.82   55  30  1   1   55  323 375 3e-09   52.4
Cnem248122  PmaxEZ420284    41.82   55  30  2   1   55  322 374 2e-07   46.6
Cnem248122  PmargHE610382   41.82   55  30  2   1   55  322 374 2e-07   46.6
Cnem248122  CgigEKC38799.1  32.20   59  39  1   1   59  319 376 8e-07   44.7
Cnem248122  Lgig232880  35.94   64  35  1   166 229 2130    2187    9e-06   42.0
# BLASTP 2.2.26 [Sep-21-2011]
# Query: Cnem6176
# Database: /Users/danieljackson/Documents/Data/Manuscripts/2013/Cepaea/Figures/
# Fields: Query id, Subject id, % identity, alignment length, mismatches, gap op
Cnem6176    Lgig231010  23.28   189 111 4   48  234 342 498 1e-06   44.3
# BLASTP 2.2.26 [Sep-21-2011]
# Query: Cnem7563
# Database: /Users/danieljackson/Documents/Data/Manuscripts/2013/Cepaea/Figures/
# Fields: Query id, Subject id, % identity, alignment length, mismatches, gap op
# BLASTP 2.2.26 [Sep-21-2011]
# Query: Cnem14344
# Database: /Users/danieljackson/Documents/Data/Manuscripts/2013/Cepaea/Figures/
# Fields: Query id, Subject id, % identity, alignment length, mismatches, gap op
# BLASTP 2.2.26 [Sep-21-2011]
# Query: Cnem821
# Database: /Users/danieljackson/Documents/Data/Manuscripts/2013/Cepaea/Figures/
# Fields: Query id, Subject id, % identity, alignment length, mismatches, gap op
Cnem821 CgigEKC35875.1  31.09   193 117 8   34  213 2326    2515    8e-20   83.2
Cnem821 PmargPUSP15 26.37   182 121 3   36  205 21  201 2e-15   69.7
Cnem821 CgigEKC42215.1  25.81   217 135 7   8   203 739 950 4e-13   63.5
Cnem821 Lgig228264  30.51   177 105 9   42  206 252 422 1e-12   61.6
Cnem821 Lgig239574  27.07   181 114 6   42  205 386 565 2e-12   61.2
Cnem821 PmaxEZ420121    34.31   102 59  3   6   102 456 554 2e-10   55.5
Cnem821 PmargNUSP1  34.31   102 59  3   6   102 806 904 8e-10   53.9
Cnem821 Lgig232022  35.21   71  39  2   42  112 256 319 1e-08   50.1
Cnem821 CgigEKC21333.1  23.68   152 91  5   12  148 185 326 1e-07   47.4
Cnem821 Lgig173138  40.62   64  37  1   39  102 18  80  2e-07   45.8
# BLASTP 2.2.26 [Sep-21-2011]
# Query: Cnem12941
# Database: /Users/danieljackson/Documents/Data/Manuscripts/2013/Cepaea/Figures/
# Fields: Query id, Subject id, % identity, alignment length, mismatches, gap op
Cnem12941   Lgig235120  31.08   148 99  2   45  190 458 604 1e-07   47.4
# BLASTP 2.2.26 [Sep-21-2011]
# Query: Cnem1323
# Database: /Users/danieljackson/Documents/Data/Manuscripts/2013/Cepaea/Figures/
# Fields: Query id, Subject id, % identity, alignment length, mismatches, gap op
Cnem1323    Lgig181237  33.66   205 121 8   5   202 133 329 9e-30    108
# BLASTP 2.2.26 [Sep-21-2011]
# Query: Cnem450
# Database: /Users/danieljackson/Documents/Data/Manuscripts/2013/Cepaea/Figures/
# Fields: Query id, Subject id, % identity, alignment length, mismatches, gap op
Cnem450 LgigB3A0P5.1    50.25   201 87  4   10  197 219 419 2e-58    185
Cnem450 Lgig114561  49.74   189 78  4   20  197 2   184 5e-57    174
# BLASTP 2.2.26 [Sep-21-2011]
# Query: Cnem16878
# Database: /Users/danieljackson/Documents/Data/Manuscripts/2013/Cepaea/Figures/
```

```
# Fields: Query id, Subject id, % identity, alignment length, mismatches, gap op
# BLASTP 2.2.26 [Sep-21-2011]
# Query: Cnem196388
# Database: /Users/danieljackson/Documents/Data/Manuscripts/2013/Cepaea/Figures/
# Fields: Query id, Subject id, % identity, alignment length, mismatches, gap op
# BLASTP 2.2.26 [Sep-21-2011]
# Query: Cnem1265
# Database: /Users/danieljackson/Documents/Data/Manuscripts/2013/Cepaea/Figures/
# Fields: Query id, Subject id, % identity, alignment length, mismatches, gap op
# BLASTP 2.2.26 [Sep-21-2011]
# Query: Cnem123
# Database: /Users/danieljackson/Documents/Data/Manuscripts/2013/Cepaea/Figures/
# Fields: Query id, Subject id, % identity, alignment length, mismatches, gap op
# BLASTP 2.2.26 [Sep-21-2011]
# Query: Cnem169764
# Database: /Users/danieljackson/Documents/Data/Manuscripts/2013/Cepaea/Figures/
# Fields: Query id, Subject id, % identity, alignment length, mismatches, gap op
# BLASTP 2.2.26 [Sep-21-2011]
# Query: Cnem1188
# Database: /Users/danieljackson/Documents/Data/Manuscripts/2013/Cepaea/Figures/
# Fields: Query id, Subject id, % identity, alignment length, mismatches, gap op
Cnem1188    Lgig190352  44.75   181 96  3   1   179 75  253 4e-49    162
Cnem1188    CgigEKC42376.1  46.24   173 88  4   10  179 136 306 1e-46    157
# BLASTP 2.2.26 [Sep-21-2011]
# Query: Cnem21112
# Database: /Users/danieljackson/Documents/Data/Manuscripts/2013/Cepaea/Figures/
# Fields: Query id, Subject id, % identity, alignment length, mismatches, gap op
# BLASTP 2.2.26 [Sep-21-2011]
# Query: Cnem28994
# Database: /Users/danieljackson/Documents/Data/Manuscripts/2013/Cepaea/Figures/
# Fields: Query id, Subject id, % identity, alignment length, mismatches, gap op
Cnem28994   Lgig163303  26.81   138 96  3   20  155 4   138 4e-06   40.0
# BLASTP 2.2.26 [Sep-21-2011]
# Query: Cnem4164
# Database: /Users/danieljackson/Documents/Data/Manuscripts/2013/Cepaea/Figures/
# Fields: Query id, Subject id, % identity, alignment length, mismatches, gap op
Cnem4164    HasiGT272916    28.15   135 74  7   1   130 54  170 1e-07   44.3
# BLASTP 2.2.26 [Sep-21-2011]
# Query: Cnem2108
# Database: /Users/danieljackson/Documents/Data/Manuscripts/2013/Cepaea/Figures/
# Fields: Query id, Subject id, % identity, alignment length, mismatches, gap op
# BLASTP 2.2.26 [Sep-21-2011]
# Query: Cnem2744
# Database: /Users/danieljackson/Documents/Data/Manuscripts/2013/Cepaea/Figures/
# Fields: Query id, Subject id, % identity, alignment length, mismatches, gap op
# BLASTP 2.2.26 [Sep-21-2011]
# Query: Cnem263
# Database: /Users/danieljackson/Documents/Data/Manuscripts/2013/Cepaea/Figures/
# Fields: Query id, Subject id, % identity, alignment length, mismatches, gap op
# BLASTP 2.2.26 [Sep-21-2011]
# Query: Cnem4282
```

```
# Database: /Users/danieljackson/Documents/Data/Manuscripts/2013/Cepaea/Figures/
# Fields: Query id, Subject id, % identity, alignment length, mismatches, gap op
Cnem4282    Lgig190352  46.30   108 57  1   1   108 390 496 2e-29    106
Cnem4282    CgigEKC42376.1  44.30   79  43  1   1   79  452 529 2e-13   60.5
# BLASTP 2.2.26 [Sep-21-2011]
# Query: Cnem3938
# Database: /Users/danieljackson/Documents/Data/Manuscripts/2013/Cepaea/Figures/
# Fields: Query id, Subject id, % identity, alignment length, mismatches, gap op
# BLASTP 2.2.26 [Sep-21-2011]
# Query: Cnem74063
# Database: /Users/danieljackson/Documents/Data/Manuscripts/2013/Cepaea/Figures/
# Fields: Query id, Subject id, % identity, alignment length, mismatches, gap op
# BLASTP 2.2.26 [Sep-21-2011]
# Query: Cnem943
# Database: /Users/danieljackson/Documents/Data/Manuscripts/2013/Cepaea/Figures/
# Fields: Query id, Subject id, % identity, alignment length, mismatches, gap op
Cnem943 Lgig193218  94.57   92  5   0   1   92  285 376 7e-62    188
Cnem943 Lgig205506  94.57   92  5   0   1   92  284 375 2e-61    187
Cnem943 Lgig202971  94.57   92  5   0   1   92  284 375 2e-61    187
Cnem943 Lgig215510  92.39   92  7   0   1   92  284 375 7e-61    185
# BLASTP 2.2.26 [Sep-21-2011]
# Query: Cnem1152
# Database: /Users/danieljackson/Documents/Data/Manuscripts/2013/Cepaea/Figures/
# Fields: Query id, Subject id, % identity, alignment length, mismatches, gap op
# BLASTP 2.2.26 [Sep-21-2011]
# Query: Cnem1504
# Database: /Users/danieljackson/Documents/Data/Manuscripts/2013/Cepaea/Figures/
# Fields: Query id, Subject id, % identity, alignment length, mismatches, gap op
# BLASTP 2.2.26 [Sep-21-2011]
# Query: Cnem104312
# Database: /Users/danieljackson/Documents/Data/Manuscripts/2013/Cepaea/Figures/
# Fields: Query id, Subject id, % identity, alignment length, mismatches, gap op
Cnem104312  CgigEKC41746.1  40.38   52  31  0   1   52  190 241 5e-11   52.0
Cnem104312  Lgig205401  50.00   32  16  0   1   32  199 230 2e-07   42.0
# BLASTP 2.2.26 [Sep-21-2011]
# Query: Cnem551
# Database: /Users/danieljackson/Documents/Data/Manuscripts/2013/Cepaea/Figures/
# Fields: Query id, Subject id, % identity, alignment length, mismatches, gap op
```
